# A GMCSF and IL7 fusion cytokine leads to functional thymic-dependent T-cell regeneration in age-associated immune deficiency

**DOI:** 10.1038/cti.2015.8

**Published:** 2015-05-08

**Authors:** Jeremy Hsieh, Spencer Ng, Steve Bosinger, Jian Hui Wu, Gregory K Tharp, Anapatricia Garcia, Mohammad S Hossain, Shala Yuan, Edmund K Waller, Jacques Galipeau

**Affiliations:** 1 School of Medicine, Emory University, Atlanta, GA, USA; 2 Department of Hematology and Medical Oncology, The Winship Cancer Institute, Emory University, Atlanta, GA, USA; 3 Yerkes National Primate Research Center, Atlanta, GA, USA; 4 The Montreal Center for Experimental Therapeutics in Cancer, Lady Davis Institute, McGill University, Montreal, QC, Canada; 5 Department of Hematology and Medical Oncology, Emory University, Atlanta, GA, USA; 6 Department of Pediatrics, Emory University, Atlanta, GA, USA

## Abstract

The competence of cellular immunity depends on a diverse T-cell receptor (TCR) repertoire arising from thymic output. Normal thymopoiesis arises from marrow-derived CD3^−^CD4^−^CD8^−^ triple-negative T-cell progenitors (TN), which develop into mature single-positive (SP) CD4 or CD8 T cells after expressing both CD4 and CD8 (double-positive, DP) transiently, leading to *de novo* T-cell production. Interleukin-7 (IL7) is a singularly important common γ-chain IL involved in normal thymic development. Our previous work has demonstrated that γ_c_ cytokines fused with granulocyte-macrophage colony stimulating factor (GMCSF) at the N-terminus acquire unheralded biological properties. Therefore, to enhance thymopoiesis, we developed a novel biopharmaceutical based on the fusion of GMCSF and IL7, hereafter GIFT7. Systemic administration of GIFT7 leads to cortical thymic hyperplasia including the specific expansion of CD44^int^CD25^−^ double-negative 1 (DN1) thymic progenitors. During murine cytomegalovirus (mCMV) infection of aged animals, GIFT7-mediated neo-thymopoiesis led to increased absolute numbers of viral-specific CD8^+^ T cell. Our work demonstrated that thymic precursors can be therapeutically repopulated and its reconstitution leads to meaningful central and peripheral T-cell neogenesis, correcting immune dysfunction arising from age-associated thymic atrophy.

Immune insufficiency secondary to aging predisposes the host to detrimental infections.^1^ Immune senescence manifested by thymic involution correlates to the progressive decline in T-cell receptor (TCR) repertoire and numerous defects in cellular immunity with severe clinical complications such as chronic inflammation, autoimmune diseases and low vaccination efficacy.^2, 3^ Normal thymopoiesis arises from marrow-derived CD4^−^CD8^−^ double-negative (DN) T-cell progenitors. Thereafter, DN develops into mature single-positive (SP) CD4 or CD8 T cells after expressing both CD4 and CD8 (double-positive, DP) transiently, leading to *de novo* T-cell production.^4^ Age-related progressive decline in thymic activity starts as early as the first year of postnatal life and is due to a combination of reduced thymic stroma and intrathymic proliferation of lymphoid precursors.^4^

Interleukin-7 (IL7) is a γ_c_-cytokine that has a key role in T-cell development and homeostasis by signaling through its cognate receptor complex IL7 receptor (IL7R)α/γ_c_.^5, 6^ Though the correlation between age-related hypotonic thymic activity and IL7 availability in the stromal niche is not clearly defined as IL7 is readily detectable in aged thymi,^7^ the use of exogenous IL7 for therapeutic lymphogenesis has been attempted. However, peripheral T-cell compartmentalization—preferential expansion of naïve and central memory CD4^+^ T cell—is a much more pronounced effect secondary to exogenous IL7 administration,^8, 9, 10^ while thymic function remains largely unaltered.^11^ Moreover, intrathymic implantation of genetically engineered IL7-producing stromal cells fails to overcome age-associated atrophy or to maintain peripheral T-cell pool despite a transient increase in intrathymic proliferation.^12^ Part of the reason for its modest therapeutic effect has to do with the homeostatic nature of IL7/IL7Rα interaction. The expression of IL7Rα is tightly regulated: it is upregulated on hematolymphoid cells destined to persist; it is downregulated on quiescent or exhausted cells, and it is internalized upon ligand binding.^13, 14^ Thus, the *in vivo* use of IL7 for regenerative immunotherapy is limited by the requirement to achieve supraphysiological concentrations to overcome immune checkpoints to its activating effects.^15^

Knowing that IL7 signaling is fundamental to T-cell fate,^16^ we attempt to overcome its limitation as a replacement therapy by utilizing an entirely synthetic cytokine signaling system. The notion therefore arises that creating a fusion cytokine borne of the physical linkage of two unrelated cytokines—a ‘fusokine'—will not only possess pharmaceutical properties ascribable to each parental domain, but may also acquire unheralded additive immune features. We have previously demonstrated the novel gain-of-function properties of granulocyte-macrophage colony stimulating factor (GMCSF)-based common γ_c_ chain fusokines,^17, 18^ including: GIFT2, GIFT15,^19, 20, 21^ GIFT21,^22, 23^ GIFT9^24^ and GIFT4.^25^ In this study, we show that engineered fusion GIFT7 delivers hyperagonistic signaling to IL7Rα/γ_c_; the unique biological consequence of such is the induction of SPCD8 and CD44^+^CD25^−^DN1 expansion in thymocyte culture. Systemic administration of GIFT7 compared with IL7 in young immune competent mice leads to a transient increase in the number of DN1 (5.48±1.01 vs 2.74±1.07 × 10^6^, *P*=0.046) by day 7. Total thymic cellularity significantly increases in GIFT7-treated group by day 14 (237±51.3 vs 123±25.6 × 10^6^, *P*=0.026). The GIFT7-mediated regenerative effect is significantly more pronounced in 9- to 15-month-old retired breeder (RB) mice where thymic involution is evident: 111±21.8, 67.8±13.9, and 60.3±4.6 × 10^6^ for seven doses of (5 μg kg^−1^) GIFT7, IL7 or phosphate-buffered saline (PBS) treatment, respectively. Interestingly, the greatest fold increase is difference observed in the CD44^int^ DN1 subset. Histologically, GIFT7-treated aged thymi demonstrate significant cortical hyperplasia. GIFT7-mediated thymopoiesis results in an enhanced viral-specific response, as evidenced by increased CD8^+^ cellularity and IL2 secretion. Importantly, GIFT7-mediated augmentation in the anti-viral response was found to be persistent after effector immunity had waned in control groups. Thus, we demonstrated that the exploitation of GIFT7-derived hypersignalling to IL7Rα/γ_c_ under conditions of thymic insufficiency led to competent repopulation of T lineage cells, highly amenable for the treatment of secondary lymphoid deficiency arising from viral, chemical and malignant insults.

## Results

### Biochemical characterization of GIFT7

**Figure 1:**
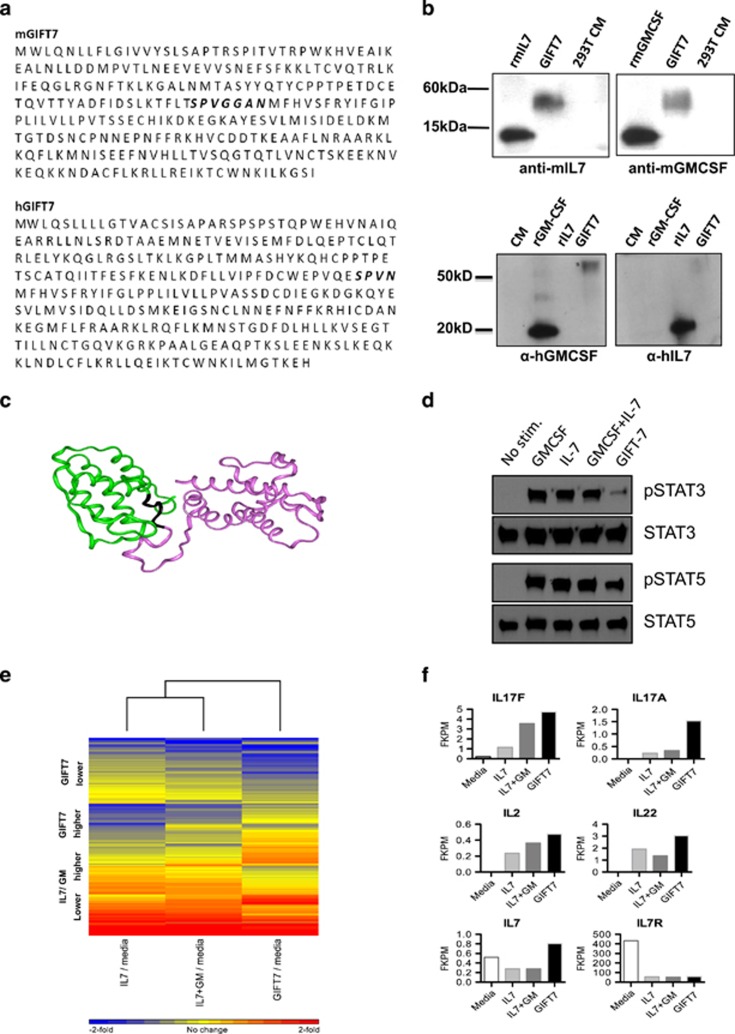
Biochemical characterization of GIFT7. (**a**) Amino-acid sequence of the fusion transgene mouse GIFT7 and its human ortholog. Schematic representation of the fusion GIFT7 showing GMCSF at the N-terminus linked to C-terminal domain IL7 by an inter-cytokine bridge consisting of *SPVGGAN* for mGIFT7 and *SPVN* for hGIFT7. (**b**) Denatured immunoblotting using supernatant derived from GIFT7 or mock-transfected 293T cells. Recombinant murine or human GMCSF or IL7 serve as positive controls. Polyclonal goat anti-mouse GMCSF and anti-IL7 antibodies or polyclonal goat anti-human GMCSF and anti-IL7 antibodies serve to probe the blot, indicating fusion protein secretion. (**c**) The homology structural model of mouse GIFT7. The GMCSF and IL7 portions are in green and pink ribbons, respectively. The link region is in black; (**d**) western blotting for phospho-specific and total STAT3 and STAT5 proteins from cell lysates of 3 × 10^5^ anti-CD3/28 bead-activated T cells previously stimulated with 1 nM GIFT-7 or cytokine controls for 15 min. (**e**) GIFT7 induces differential transcriptional program over IL7. Illumina deep sequencing (RNA-seq) was used to identify gene expression of PBMCs treated with hGIFT7, hIL7 alone, or hIL7+hGMCSF and media controls. 560 genes were identified as maximally different between treatment groups, visualized using hierarchical clustering. (**f**) RNA-seq profiles of genes expression pattern as fold-change difference in the IL7 and IL17 pathway (*n*=1).

We engineered a *G*MCSF–*I*L7 *F*usion *T*ransgene (GIFT7) by cloning mGMCSF (murine GMCSF) cDNA in frame at the N-terminus of full-length murine IL7 cDNA. The resulting sequence of the fusion transgene was confirmed by sequence analysis (Figure 1a). Immunoblotting of the conditioned medium derived from GIFT7-transfected mammalian cell line demonstrated that the GIFT7 fusion protein is translated, secreted, and recognized by both α-GMCSF and α-IL7 antibodies (Figure 1b). Computer-based homology structural model of the resultant GIFT7 protein is shown (Figure 1c). To predict the possible binding mode of GIFT7 with IL7Rα, we *in silico* aligned the IL7 portion of GIFT7 with the crystal structure of IL7 in complex with IL7Rα. The GMCSF portion of GIFT7 was not in steric clash with IL7Rα (Supplementary Figure S1A). To investigate whether GIFT7 imparts distinct signaling property on receptor-expressing cells, the analysis of the activation status of common-gamma chain associated STAT5 and IL-7Rα associated STAT3 signal transduction molecules reveals an asymmetric signal transduction profile on GIFT7 treated, anti-CD3/28 bead-activated T cells. GIFT7 (1nM) induces STAT5 phosphorylation at levels comparable to GMCSF and IL7 but fails to induce a similar phosphorylation of STAT3 on activated T cells, suggesting a partial agonistic effect of GIFT7 on the IL7 receptor complex (Figure 1d). This effect was much more pronounced in activated T cells compared with naïve, primary T cells (Supplementary Figure S1B). While STAT5 phosphorylation is critical for the activation of effector functions in T cells, STAT3 activation acts in a more regulatory manner to maintain homeostasis and to limit proinflammatory T-cell responses. Upon phosphorylation, these STAT molecules translocate to the nucleus where they alter their transcription patterns. The observation that GIFT7 drives an unopposed STAT5 response (that is, in the absence of STAT3 activation) led us to believe that GIFT7 treatment might impart a unique transcriptomic signature on lymphomyeloid cells compared to treatment with GMCSF and IL7.

To further investigate the distinctive signaling property of GIFT7, we examined the transcriptional profile of peripheral blood mononuclear cells (PBMCs) treated with GIFT7, IL7 alone, IL7 in combination with GMCSF, and media controls using RNA-seq (Figure 1e). While all three treatments induced a common set of genes, we identified a set of 560 genes that were uniquely expressed in one or more treatment condition relative to the others (Supplementary Table 1). To organize these differentially expressed into expression patterns, we used unsupervised clustering on both genes and biological groups. Using the algorithm *a priori*, several patterns of gene expression became evident: (i) a cluster of genes induced by GIFT7 but not by the other treatments; (ii) several genes that were downregulated by only GIFT7 but not by other treatments; (iii) a class of genes that were induced by all three treatments but were remarkably higher in GIFT7-treated samples compared with IL7; or (iv) a small number of genes that were uniquely induced by IL7+GMCSF but not by IL7 alone or GIFT7. Overall, the numbers of genes that were uniquely regulated between IL7+GMCSF and IL7 alone were small compared with those uniquely regulated by GIFT7, which is reflected by the shorter distance of these treatments compared with GIFT7 (Figure 1e). We focused our analysis on known genes in the T-cell differentiation and IL7 pathway (Figure 1f). Of note, the expression of the IL7R was observed to be downregulated in all three different treatment groups relative to media—providing an internal biological validation of the assay. Interestingly, IL7 itself had higher expression in GIFT7-treated samples compared with the IL7 and IL7+GMCSF groups, as was IL2, suggesting that GIFT7 signaling may exert its effect by induction of lymphocyte growth factors. Finally, we noted that GIFT7 induced a remarkably higher expression of IL17A, and to a lesser extent, IL17F, but not the non-canonical IL17 members (Figure 1f). IL22, whose expression is tightly linked to IL17,^26^ was also observed to be elevated in the GIFT7-treated groups relative to the IL7 treatments.

### GIFT7-treated DN and SPCD8 resist apoptosis *in vitro*

**Figure 2:**
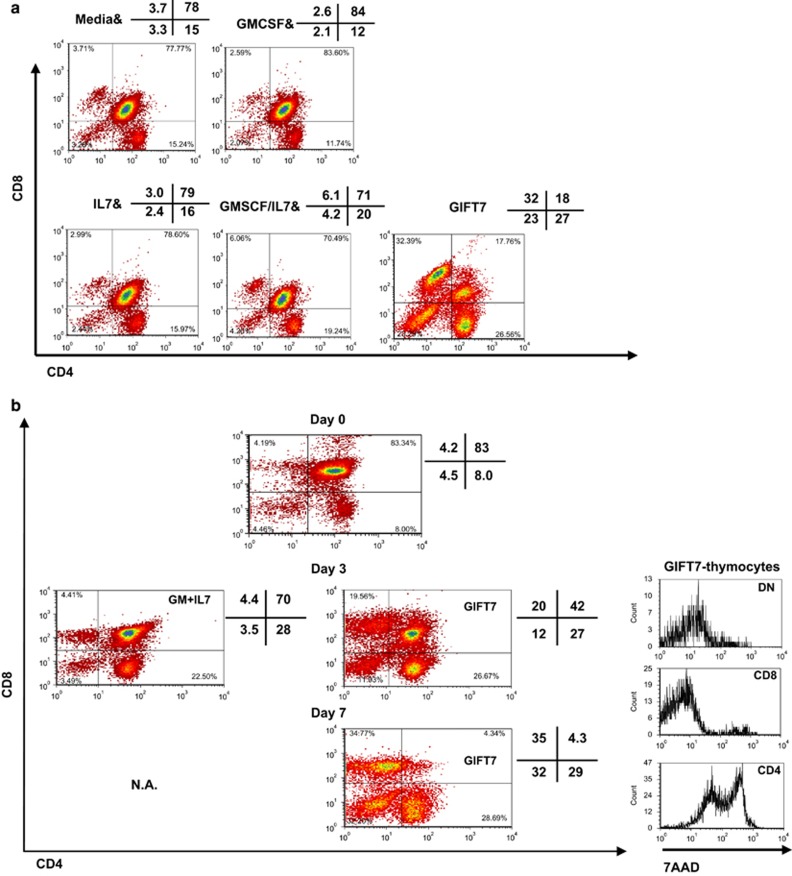
GIFT7 mediates preferential expansion of SPCD8 and DN thymocytes. (**a**) The fusion GIFT7 leads to selective expansion of thymocytes *in vitro.* Dissociated thymi were cultured in the presence of GIFT7 (10 ng  ml^−1^) or cytokine controls (20 ng ml^−1^) for 5 days. Numbers represent the percentage of recovered cells in each quadrant. (**b**) Dot plots show the expression of CD4 and CD8 by thymocytes cultured in the presence of control cytokines (20 ng ml^−1^) or GIFT7 (10 ng ml^−1^) recovered on days 0, 3 or 7. Numbers indicate percentages of cells in each quadrant. Histogram of 7-Amino-actinomycin D (7-AAD) staining shows membrane integrity (cell viability) of three different subsets of GIFT7-treated thymocytes after 7-day culture. The assay was performed three times with representative dot plot shown.

We next sought to investigate the effect of GIFT7 on thymic T-cell precursors. Thymocytes were isolated from 6- to 8-week-old mice and cultured in the presence of GIFT7 (10 ng ml^−1^) or control cytokines. Analysis of cultured thymocytes at day 5 demonstrated that GIFT7 promoted selective persistence of DN and SPCD8, while the ratio of thymic subsets was virtually unchanged with other cytokine treatments compared with media alone (Figure 2a). The expansive effect seemed to be saturated at 10 ng ml^−1^ (Supplementary Figure S2a) as 50 and 100 ng ml^−1^ concentrations appeared to induce similar level of changes. The dose-dependent effect of GIFT7, however, was clearly evident on T cells derived from PBMCs where increasing doses of GIFT7 leads to progressive CFSE dilution (Supplementary Figure S2b). Similarly, DN and SPCD8 subsets increased in percentage over time with DN and SPCD8 representing 32 and 35%, respectively, in the GIFT7-treated thymocyte culture at day 7 (Figure 2b). GIFT7-treated DN and SPCD8 showed significantly less 7AAD inclusion compared with SPCD4 (Figure 2b). CFSE-pulsed sorted DN cells showed significant dilution after GIFT7 treatment in both TCRγδ^−^ and TCRγδ^+^ compartments, demonstrating the mitogenic effect of GIFT7 on DN subset in both αβ^+^ and γδ^+^ lineage (Supplementary Figure S3A).

We pre-sorted DN, SPCD4, SPCD8 and DP thymocytes and subsequently stimulated them with GIFT7 for 9 days to test the hypothesis that GIFT7 modulates early T-cell differentiation. We demonstrated that GIFT7 alone did not significantly alter the expression of CD4 and CD8 on pre-sorted cells (Supplementary Figure S3B). In all, these data suggested that GIFT7 leads to the survival and expansion of DN early thymic progenitors and SPCD8 thymocytes *in vitro* without influencing their differentiation programming.

### GIFT7 leads to transient thymic hyperplasia in immune-competent young mice

We next investigated the systemic effect of GIFT7 administration on thymic tissue in normal 6- to 8-week-old adult mice. In this experiment, we compared the effect of GIFT7 to IL7 on thymic cellularity analyzed at days 7, 14 or 35 post treatment (Supplementary Figure S4A). On day 7, both IL7 and GIFT7-treated mice showed a significant increase in total thymic cellularity, which continued to rise in the GIFT7-treated group but started to decline in the IL7 group by day 14 (237±51.3 vs 123±25.6 × 10^6^, *P*<0.05) (Supplementary Figure S4B). Importantly, GIFT7 increased the percentage and the total number of DN1 cells by day 7 (5.48±1.01 vs 2.74±1.07 × 10^6^, *P*<0.05) (Supplementary Figure S4C). The ratio of thymic subsets normalized between these two groups by day 1, but the increase in total thymocytes and DP with GIFT7 administration remained significant (Supplementary Figure S4D). On day 35 post injection, total thymic cellularity in both GIFT7- and IL7-treated groups declined to a level similar to that of before treatment (Supplementary Figure S4B). Here, we demonstrated that GIFT7 was able to induce transient and reversible thymic hypertrophy. DN1 thymic progenitors appeared to be early GIFT7 responders, with concomitant increase in DP and total thymocytes at later stages.

### GIFT7 corrects age-related thymic atrophy and expands the CD44^int^ DN subset

**Figure 3:**
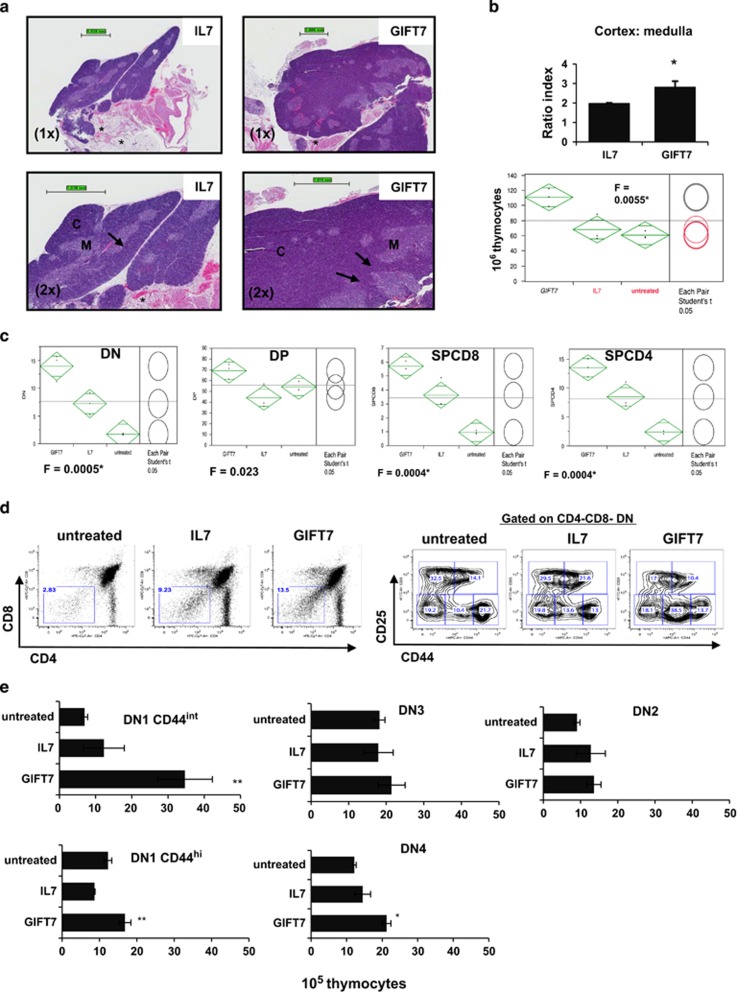
GIFT7 corrects age-related thymic atrophy and leads to CD44^int^ DN expansion. (**a**) Hematoxylin- and eosin-stained paraffin sections of thymus from one representative GIFT7- or IL7-treated aged mice. Higher magnification shows cortical hyperplasia infiltrating the lining of cortical medullary junction (solid arrow) compared with the smooth lining in IL7-treated group (interrupted arrow). Asterisk, adipose tissue deposit; C, cortex; M, medulla. (**b**) The histogram indicates the ratio of cortical/medullary thickness. Data represent the mean value±s.d. (*n*=6); *P<0.05. ANOVA plot shows the number of total thymocytes in each group with the result of pair Student's *t*-test shown on the right. (**c**) Increase in the number of DN, DP, SPCD4 and SPCD8 thymocytes of the GIFT7-treated aged mice. Thymi were dissociated and analyzed by flow cytometry. ANOVA plot represents the mean number of cells ±s.d. with analysis of variance, F and pair Student's *t-*test shown on the right (*n*=6). (**d**) Dissocaited thymi were further characterized using CD4, CD8, CD44 and CD25 surface molecules. Representative flow cytometry plots indicate GIFT7-mediated expansion in the frequency of CD44^int^CD25^−^ DN thymocytes. Number represents the percentage in each gated region. (**e**) Increase in the number of total, DN4 and both CD44^int^ and CD44^hi^ DN1 in GIFT7-treated aged mice. Histogram represents the mean number of cells ±s.d. (*n*=6).

Given the significant expansive effect of GIFT7 on young thymic tissue both *in vitro* and *in vivo*, we next investigated whether GIFT7 could correct age-related thymic involution. Here, we utilized RB mice (9–15 months old) as our sources of atrophic thymi. Mice were treated with 7 doses of IL7 or GIFT7 (5 μg kg^−1^) every other day. On day 28 post treatment, hematoxylin/eosin (H&E) staining on thymic tissues of IL7-treated aged mice showed extensive adipocyte deposit (*), reduced cortical thymic tissue (cortex:medulla ratio 2:1) with intact cortical medullary thymic lining (*interrupted arrow*) whereas GIFT7-treated mice exhibited minimal adipocyte deposits (*), hypercellularity in the cortex (cortex:medulla ratio 2.67:1) and disrupted cortical-medullary lining with thymocytes infiltrating the medulla, indicating foci of proliferation (*solid arrow*) (Figures 3a and b). In accordance with the histology data, GIFT7-treated mice had significantly higher number of total thymocytes (111±22, 68±14, 60±4.6 × 10^6^, for GIFT7-, IL7—treated and untreated—groups, respectively. F=0.0055 by one-way ANOVA). Comparison for each pair showed that thymic cellularity of GIFT7-treated mice is significantly greater than that of untreated (*P*=0.0027) or IL7-treated (*P*=0.0059) groups; whereas analysis of IL7—and untreated—groups yield no significant difference (*P*=0.49) (Figure 3b). Cellular expansion was observed in DN, SPCD8 and SPCD4 (F=0.0005, 0.0004 and 0.0004, respectively). Specifically, GIFT7-DN exhibited the greatest fold difference compared with IL7 or untreated groups (Figure 3c). To assess whether GIFT7 mediated DN expansion enhanced *de novo* T-cell production, we measured the mRNA level of single-joint T-cell Receptor Excision Circle (TREC) per TCRα in total splenic DNA as a surrogate for thymic emigrants in the periphery (Supplementary Figure S5A). Two out of three mice in the GIFT7-treated group showed a significant increase in peripheral sjTREC, suggesting a trend of recent thymic emigrants populating the periphery. To further analyze the DN subsets responsive to GIFT7 stimulation, we showed a significant increase in the percentage of CD44^int^CD25^−^ subset in the DN population (38.5, 13.6 and 10.4% for GIFT7, IL7 and untreated groups, respectively) (Figure 3d). In fact, there was a fourfold increase in the number of CD44^int^ DN compared with thymocytes derived from untreated mice. Furthermore, expansion in the number of CD44^hi^DN1 and DN4 cells was also observed (Figure 3e). We also found significant downregulation of IL7Rα expression on CD44^hi^CD25^−^ population in mice treated with GIFT7, suggesting receptor internalization upon stimulation (Supplementary Figure S5B). To better characterize the proliferative effect of GIFT7 and IL7 on DN subsets, we cultured purified DN cells *in vitro* and analyzed Ki67 after cytokine stimulation. Consistent with our *in vivo* observation, the most significant Ki67 induction was observed in GIFT7-treated CD44^int^DN1 subset, exhibiting 150% increase in mean fluorescence intensity (MFI) over isotype staining (Supplementary Figure S5C). Overall, our data suggest that GIFT7 administration leads to CD44^int^ DN expansion *in vivo*.

### GIFT7-treated aged animals restored murine cytomegalovirus-specific CD8 T-cell response comparable to that of young animals

**Figure 4:**
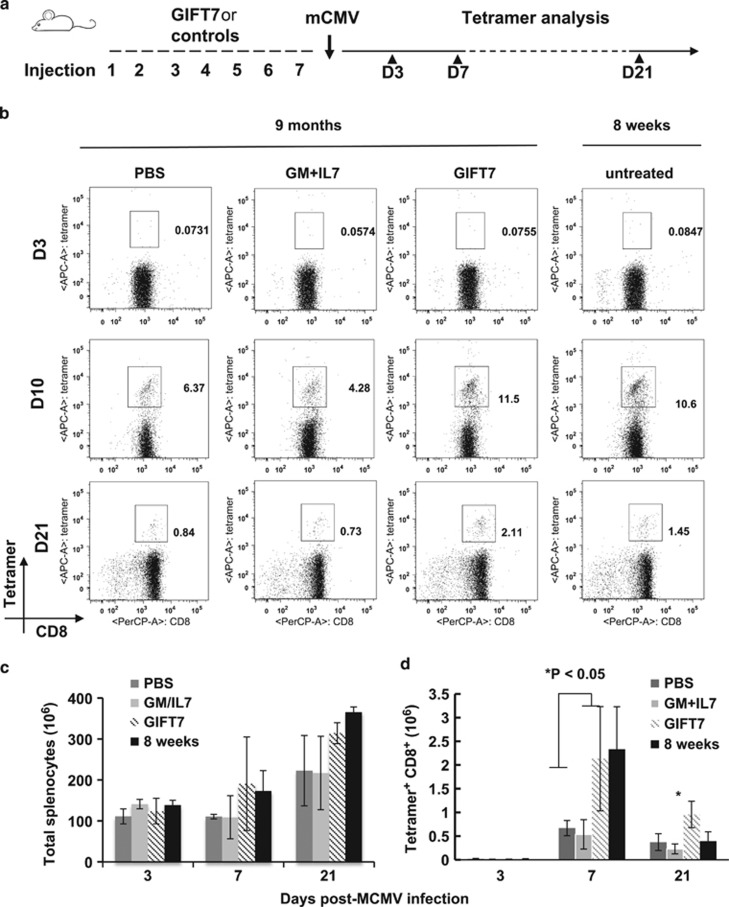
GIFT7 enhances anti-mCMV CD8^+^ T-cell immunity in aged animals. (**a**) Schematic representation indicates seven i.p. injections of GIFT7 or cytokine controls at 5 μg kg^−^^1^ in RB mice. 1 × 10^5^ plaque-forming unit (PFU) of mCMV was injected 7 days after the last cytokine injection. Spleens were analyzed for total or viral-specific cellularity on days 3, 7 or 21 (arrowhead) post viral infection by mMCV peptide MHC tetramer staining. (**b**) Flow analysis shows the percentage of mCMV tetramer^+^CD8^+^ T cells in different age and treatment groups at different time points. Splenocytes in single cell suspension were stained for CD3, CD4, CD8 and mCMV peptide MHC tetramer, and gated on CD3^+^CD4^−^ cells. (**c**) Histogram shows mean total splenic cellularity of nucleated cells from different treatment groups at different time points ±standard deviation (*n*=4). (**d**) Absolute numbers of mCMV tetramer^+^ CD8^+^ T cells were quantified based on flow-cytometry analysis. Histogram shows mean cellularity per spleen ±standard deviation (*n*=4). The experiment was repeated twice with similar results.

Latent cytomegalovirus (CMV) reactivation frequently correlates with immune compromised state. In post bone marrow transplant, viral clearance depends on functional T-cell reconstitution; in the elderly population, sufficient nascent T-cell output maintains viral inactivation.^1, 27, 28^ To investigate whether GIFT7-mediated thymopoiesis in aged mice would lead to an augmentation of acquired anti-viral immunity, we challenged IL7-, IL7+GMCSF-, GIFT7- or PBS-preconditioned mice with a non-lethal dose of murine CMV (mCMV) 7 days post cytokine treatment. Mice were killed at day 10 post viral infection. To measure the response of mCMV-specific T-cell immunity, we enumerated peptide MHC tetramer+ T cells in the spleen (Supplementary Figure S6A). GIFT7 treatment significantly increased the number of mCMV-peptide MHC tetramer^+^ T cells in the spleen (2.26±0.66 vs 1.2±0.51 × 10^6^ for GIFT7 and untreated, respectively, *P*<0.05) (Supplementary Figure S6B). To distinguish central (that is, thymopoiesis) vs peripheral contribution over the observed enhancement in viral CTL response, we repeated the experiment in thymectomized RB mice (Supplementary Figure S6C) and demonstrated that the number of mCMV-specific CD8 T cells in GIFT7-treated mice was not significantly different from other treatment groups (Supplementary Figure S6D). To better understand the kinetics and the magnitude of GIFT7-mediated CTL response and to see how it compares with that of young 8-week-old mice, we repeated the experiment where cytokine-treated and mCMV-challenged RB mice were killed on 3, 7 or 21 days after viral inoculation to enumerate the development of anti-mCMV T-cell immunity in the spleen, as measured by peptide MHC tetramer staining (Supplementary Figure 4A). We included untreated young (8 weeks) animals in the analysis as a comparison to demonstrate the magnitude and kinetics of viral response secondary to cytokine treatment. On day 3, the frequency of tetramer^+^ CD8 T cells was <0.1% in both young and aged animals of all treatment groups. On day 7, among the aged groups, GIFT7-treated animals had significantly higher frequency of mCMV peptide MHC tetramer+ CD8 T cells compared with PBS- or GMCSF+IL7- treated animals. On day 21 post viral challenge, we continued to detect elevated frequency of mCMV-specific CD8 T cells in the spleen derived from GIFT7-treated animals. The magnitude of GIFT7-mediated augmentation of mCMV-specific CD8 T-cell response on day 7 and 21 was comparable to the frequency detected in young animals (Figure 4b). Total live nucleated cells from the spleen were enumerated at different time points. Progressive increase in total splenocytes was detected from day 3 to day 21, but no significant difference was observed between different treatments or age groups at any given time point (Figure 4c). In terms of viral-specific cellularity, GIFT7-treated aged animals have significantly higher number of mCMV-specific CD8 T cells compared with PBS- or GMCSF+IL7-treated groups (*P*<0.05) on day 7 and all other groups including young animals (*P*<0.05) on day 21 (Figure 4d). Our data suggested that GIFT7-mediated thymopoiesis contributed to an increase in peripheral T cells poised to respond to a defined viral pathogen.

### Tetramer^+^CD8^+^ or CD4^+^T cells from GIFT7-treated group have higher frequency of IL2 and CD25 expression, respectively

**Figure 5:**
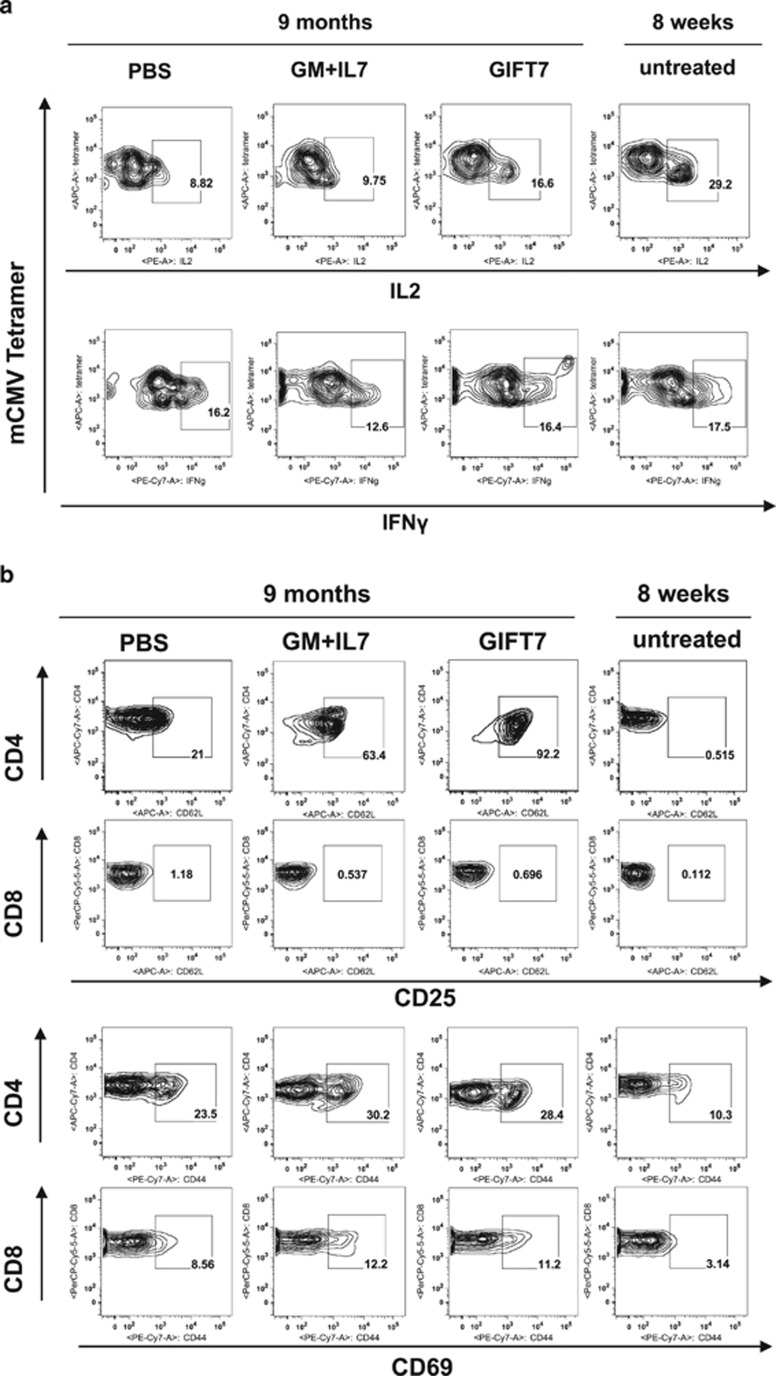
mCMV-specific CD8^+^ T cells and CD4^+^ T cells from GIFT7-treated group express higher level of IL2 and CD25, respectively. (**a**) Flow analysis of mCMV tetramer^+^CD8^+^ T cells or (**b**) total splenocytes in different age and treatment groups at day 7 post infection shows the profile of effector cytokine production or activation surface markers. PMA/ionomycin activated splenocytes were stained for CD3, CD4, CD8, mCMV peptide MHC tetramer and IL2/IFNγ after permeablization. Total splenocytes were stained for CD3, CD4, CD8, CD25 and CD69, and gated on CD3^+^CD4^+^ or CD3^+^CD8^+^ cells.

To further investigate the impact of GIFT7 on the restoration of aged T-cell immunity, we analyzed cell functionality per surface staining and cytokine secretion. We isolated splenocytes derived from animals on day 7 post mCMV infection and stimulated them *in vitro* with PMA/ionomycin. mCMV-specific CD8 T cells from GIFT7-treated group have enhanced propensity to produce IL2, but not IFNγ, compared with PBS or GMCSF+IL7 treated animals. GIFT7-treated aged animals have significantly higher frequency of IL2-producing mCMV tetramer+ CD8 T cells upon restimulation; however, young animals had the highest frequency of IL2^+^mCMV-tetramer^+^CD8^+^ splenocytes. No significant difference was detected in the frequency of IFNγ-producing mCMV tetramer^+^ CD8 T cells between different age and treatment groups (Figure 5a).

Next, we compared the expression levels of activation markers on the cell surface of CD4^+^ and CD8^+^ T cells derived from GIFT7-treated group and others at day 7 post infection. Flow analysis revealed that CD4^+^ T cells from GIFT7-treated group had significantly higher percentage of CD25 expression; whereas, the CD8^+^ T cells among all the groups were almost devoid of CD25 expression. Moderately increased level of CD69 expression was detected in CD4^+^ and CD8^+^ T cells from GMCSF+IL7- and GIFT7-treated groups (Figure 5b). No difference was detected in the level of expression in CD44 or CD62L surface molecules among all treatment groups on day 7 or day 21 (data not shown).

## Discussion

Several regenerative approaches have been explored with the aim of restoring thymopoiesis in animal models of aging.^12, 29, 30, 31, 32, 33^ None to date have been entirely satisfactory. Keratinocyte growth factor,^34, 35^ growth hormone^36^ or ghrelin^29^ reorganize thymic architecture by acting on thymic epithelial cells with only modest effect on lymphoid progenitors. γ_c_ cytokine replacement therapy appears to have more impact on peripheral T-cell pool than *de novo* production.^10, 11, 12^ Here, we describe a novel thymic trophic approach by providing direct mitogenic signals to T-cell precursors via IL7Rα/γ_c_ axis. We demonstrate that systemic administration of GIFT7 into young mice drives the expansion of thymic progenitors (DN1) resulting in a transient and reversible increase in total thymic cellularity by 2 weeks (Supplementary Figure S4B). This is not achievable via physiological signaling of IL7Rα/γ_c_ as previous studies have demonstrated limited impact of IL7 supplementation on thymic ratio under immune competent conditions.^11, 12^ We further explore the utility of GIFT7 by harnessing its non-physiological signaling on RB mice with atrophic thymic tissue where the GIFT7-mediated hypertrophic thymopoiesis appears to be significantly more pronounced. The reduction of survival niche in the RB thymi responds to GIFT7 in a dramatic manner: hypertrophy of cortical tissue at the cortical-medullary junction (Figure 3a), and a fourfold increase in CD44^int^ DN (Figure 3e). The increase in the RNA level of sjTREC per splenic TCRα^+^ T cells in two out of three GIFT7-treated animals suggests a trend, though limited by the experimental numbers, of GIFT7-medated *de novo* T-cell regeneration detected at the periphery (Supplementary Figure S5A). Sustained postnatal T-cell development depends on the continuous supply of bone marrow-derived hematopoietic stem cells as resident thymocytes are deemed to possess limited self-renewing capacity. This is evident in that mature T cells are rapidly replaced by BM sources after transient detection of thymic output in the periphery after thymic transplants.^7^ However, Allman *et al.*^37^ shows that early thymic progenitors and BM common lymphoid progenitors are phenotypically diverse and postulates that thymic T lineage precursors represent a different route than BM common lymphoid progenitors. Matins *et al.*^38^ describes thymic-autonomous T-cell development with normal intrathymic differentiation and TCR diversification in the events of defective receptor tyrosine kinase Kit and γ_c_ signaling localized at the BM compartment. Their studies show that by eliminating BM-derived competition for thymic survival niche, resident thymocytes exhibit full self-renewing capacities.^38^ Peaudecerf *et al.* describes a population of thymic precursors with the capacity to persist in the complete absence of functional bone marrow supply and defined them phenotypically as CD3^−^CD4^−^CD8^−^CD44^+^CD25^low^IL-7R^low^ TN1-TN2 cells.^38, 39^ Interestingly, both studies point to the importance of bone marrow-specific IL7-IL7Rα/γ_c_ axis disruption in restoring intrathymic self-renewal. Thus, the continuous replacement of thymic progenitors by the bone marrow represents the competition for survival signals (that is, IL7) in the thymic niche instead of an intrinsic hierarchy of differentiation potential between bone marrow derived vs resident thymic T-lineage precursors. In line with this finding, our studies demonstrate that providing hypertonic STAT5 signaling *in situ* (that is, GIFT7) also contributes to T-cell neogenesis—especially under age-related immune insufficient state—via a route that is led by intrathymic proliferation and independent of BM modulation. However, the long-term effect of GIFT7-mediated thymopoiesis and reconstituting potential of the CD44^int^ DN1 subsets remain to be assessed.

The reduction in repertoire diversity represents an important cause of weakened host immunity during aging.^33^ Indeed, thymic dysfunction can complicate numerous senescence-related immune pathologies; pharmacological regeneration of host immunity thus has a wide range of clinical applications.^40^ We investigated the utility of GIFT7-mediated T-cell regeneration by demonstrating the repopulation of resident thymic precursors increases the absolute numbers of viral-specific CD8^+^ T cells at day 7 post infection. Impressively, the level of GIFT7-mediated immune restoration in aged animals was comparable to that of young mice (Figure 4d). The effect was found to be persistent at least after 21 days. In fact, we found the frequency and absolute numbers of viral-specific CD8^+^ T cells were significantly higher in the GIFT7-treated group, even compared with young animals, at day 21 post infection when the level of PBS- or GMCSF+IL7-treated groups was barely detectable. In essence, GIFT7 significantly increases the magnitude and the persistence of anti-viral CD8 response, but did not alter the kinetics at which adaptive immunity responds during the early phase of an acute infection. This can be explained at least in part by increased IL2-signaling activity detected in GIFT7-treated animals: we found that mCMV tetramer^+^ CD8^+^ T cells have higher level of IL2 production upon restimulation and the entire CD4^+^ compartment had upregulated IL2Rα. This is consistent with the previous reports where IL2-derived paracrine signaling contributes to the *in vivo* persistence of memory T cell.^41, 42^ Supporting this hypothesis that GIFT7 may act to preserve the fate of memory T cells, we have found that GIFT7 acts preferentially to induce a STAT5 response in activated, memory-precursor T cells (IL7Rα^hi^) compared with naïve T cells (Figure 1d and Supplementary Figure S1B). Akin to our observations, CD44^int^ memory CD8^+^ T cells were shown to be the predominate pro-inflammatory cytokine-secreting cell population upon influenza NP_366-374_ peptide re-stimulation of F5 TCR transgenic mice.^43^ Thus, we propose that GIFT7 treatment corrects age-associated immune deficiency during acute viral infection by (a) expanding the pool of thymic-derived *de novo* T cells available in responding to neo-antigens and (b) converting antigen-experienced effector T cells to a phenotype (IL2^+^tetramer^+^CD8^+^ and IL2Rα^+^CD4^+^) more amendable for longer *in vivo* persistence.

Activation strength is integral to the changes of nuclear gene expression profile.^44, 45^ Augmented or truncated activity often leads to the induction of non-canonical signaling, a familiar narrative in the pathogenesis of malignancy. In the case of GIFT7, we demonstrate that treatment of activated T cells results in the activation of STAT5 alone, without a concomitant activation of STAT3 (Figure 1d). Our observations are in line with previously published reports demonstrating that constitutive activation of STAT5 dramatically enhances T-cell survival via a Bcl-2-dependent mechanism.^46^ This observation is unsurprising given that Bcl-2 is positively regulated by STAT5, while STAT3 activation has been shown to inhibit Bcl-2 transcription.^47^ Furthermore, STAT5 activation has been shown to play a particularly important role in the expansion and preservation of memory T cells in the context of lymphocytic choriomeningitis virus (LCMV) infection; a result we have recapitulated using GIFT7 to augment STAT5 activity in a mCMV model here^46^ GIFT7, while acting biochemically as a partial agonist, ultimately results in a hyperagonistic biological effect by driving an unopposed STAT5 signal. This is presumably due to atypical engagement and clustering of GMCSF and IL7 receptor complexes, an engineered feature of the GIFT fusions well described in our previously published work.^24, 48^ Moreover, our genome-wide RNA sequence analysis of cytokine-treated T cells confirms that GIFT7 induces a differential program of gene expression than IL7. In essence, it shows that genes induced by IL7 and IL7+GM-CSF are overall more similar to each other than to the GIFT7 response. Interestingly, the gene profile of GIFT7-treated T cells also correlate with the upregulation of IL17-related genes, IL17A (2.8-fold change) and IL22 (8-fold change). In contrast to their earlier ascribed roles as short-lived effector cells, the memory characteristics of IL17-producing T cells have been unveiled and extensively studied as they have a propensity to persist *in vivo* while retaining significant cytolytic capacity as demonstrated in a number of tumor models.^49, 50, 51^ Thus, our data strongly suggest that GIFT7 imparts distinct signal transduction, gene transcription and cellular phenotype.

Delayed reconstitution of the adaptive immunity post-HSCT significantly increases the risk of opportunistic fungal and viral infections for the recipients.^4^ This is of grave concern particularly for the elderly population as the normalization of their T-cell compartment derives primarily from extrathymic expansion.^52, 53^ The consequence of such is a further delay in immune recovery without the regeneration of TCR repertoire diversity required for an efficient response against neo-antigens.^54, 55^ As IL7 has been shown to accelerate immune recovery in both murine model of bone marrow transplant^56^ and patients of post-allo-HSCT,^57^ it is worthwhile to test the hypothesis of using GIFT7 as an immune regenerative agent in the setting of allo-HSCT of the elderly. Of note, exogenous IL7 does not seem to promote GvHD in a conclusive manner, presumably because IL7-responsive subsets in the setting of lymphopenic recovery seem to reside in the effector memory/non-alloreactive compartment.^56, 58^ We therefore propose the use of GIFT7 or GIFT7-enhanced T-cell precursors for the treatment of human thymic hypoimmune ailments with unmet clinical needs.

## Methods

### Animals, reagents and cell culture

Six to eight weeks old or RB C57Bl/6 female mice used experimentally were purchased from the Jackson Laboratory (Bar Harbor, ME, USA). We use 10- to 15-month-old RB mice were used as our animals of thymic involution. Thymectomized RB C57Bl/6 female mice were also purchased from Jackson Laboratory. Recombinant murine GM-CSF, IL7 and their antibodies were purchased from R&D systems (Minneapolis, MN, USA). Dulbecco's Modified Eagle's Medium (DMEM), RPMI-1640, fetal bovine serum and Penicillin/Streptomycin were purchased from Wisent Technologies (Rocklin, CA, USA). Human embryonic kidney 293 cell line was cultured in DMEM (Wisent Technologies) supplemented with 10% FBS (Wisent Technologies) and 100 U ml^−1^ of Penicillin/Streptomycin (Wisent Technologies). Primary mouse thymocytes were cultured in RPMI supplemented with 2 mM L-glutamine, 1mM HEPES, 1mM sodium pyruvate, 0.05 mM β-mercaptoethanol with 10% FBS (Complete media). Spleen-derived T cells were cultured in complete media. WT C57B/L6 or RB thymic-derived DN cells were purified via antibody-magnetic beads. Briefly, thymi were dissociated and re-suspended at 1 × 10^8^ cells ml^−1^. Cell suspension was labeled with a cocktail of biotinylated CD3/CD4/CD8 antibodies (1 μg ml^−1^), and subsequently labeled with secondary anti-biotin selection antibody at 100 μl ml^−1^. DN cells were purified using magnetic column after labeled with magnetic nanoparticles (STEMCELL, Vancouver, BC, Canada).

### Fusokine generation and protein modeling

To clone the fusion transgene GIFT7, mouse GMCSF cDNA was cloned in frame to mouse IL7 cDNA (Invivogen, San Diego, CA, USA) to generate GIFT7 fusion transgene. Human embryonic kidney 293 cells were transiently transfected with GIFT7 transgene using PolyFect (Qiagen, Mississauga, ON, Canada); serum-free DMEM supernatant was collected after 48 h and concentrated using Amicon centrifugation columns (Millipore, Cambridge, ON, Canada). Western blot analysis and cytokine ELISA (GMCSF and IL7) were used to confirm the expression and concentration of GIFT7. Concentrated conditioned media were used in all *in vitro* assays. Human embryonic kidney 293 T cells transfected in the same manner in the absence of the plasmid containing the fusion transgene were used as a mock-transfected control.

The three-dimensional structural model of mouse GIFT7 was obtained by homology modeling using MODELLER 9v3 (University of California at San Francisco). Crystal structures of human GM-CSF (PDB entry: 2gmf) and IL7 (PDB entry: 3di2) were used as the templates for homology modeling. Crystal structure of tryptophanyl-tRNA synthetase homolog (PDB entry: 3hzr) was identified as the template for the link region and the N-terminus of IL7. Among the 300 structural models of GIFT7 generated, the one with the lowest objective function was selected for further analysis.

### The generation of human ortholog of GIFT7

We engineered the human ortholog of GIFT7 by cloning human GMCSF (mGMCSF) cDNA in frame at the N-terminus of full-length human IL7 cDNA controlled by the pORF promoter. The resulting sequence of the fusion transgene was confirmed by sequence analysis. Concentrated media conditioned by 293T cells transfected with pORF-hGIFT7 was used to measure the expression and activity of hGIFT7.

### T-cell signaling

Primary T cells were isolated from the spleen 6-month-old C57Bl/6 mice by negative selection following the manufacturer's recommendations (Stem Cell Technologies, Vancouver, CA, USA). T cells were expanded with anti-CD3/28 microbeads according to the manufacturer protocols (Life Technologies, Grand Island, NY, USA) for 3 days before the beads were removed by magnetic separation. The expanded T cells were seeded at 2 × 10^6^ cells ml^−1^ and allowed to rest for 2 h in RPMI (FCS-free) before stimulation with GIFT7 or equimolar cytokine controls (R&D Systems) for 15 min. Cells were immediately lysed and probed by western blot with rabbit phospho-specific and total STAT3 and STAT5 antibodies (Cell Signaling, Danvers, MA, USA).

### RNA-seq analysis

Whole blood sample were obtained from healthy volunteers. PBMC was isolated by Ficoll gradient centrifugation at 1.85 × 10^3^ r.p.m. for 30 min. Isolated PBMC was cultured in RPMI complete media supplemented with α-CD3/CD28-coated beads (Invitrogen). After 48 h, dynabeads were washed off by incubating cell suspension in EasySep Magnet (STEMCELL) for 5 min. PBMC culture was replenished with fresh RPMI complete media supplemented with the human ortholog of murine GIFT7 (20 ng ml^−1^) or equimolar concentration of hIL7 or hIL7+hGMCSF for 3 days. Cells were obtained and live CD3+ cells were sorted by flow cytometry. The RNA from sorted cells was extracted using QIAGEN RNeasy kits according to the manufacturer's protocol. RNA quality was assessed using an Agilent Bioanalyzer (Agilent Technologies, Santa Clara, CA, USA), and RNA with RIN scores >8.0 were using for RNA-seq. RNA-seq libraries were sequenced on an Illumina HiSeq producing 2 × 100 paired end reads. Reads were assessed with FastQC and had a Q20 depth of 9.95–14.7 Million. Reads were mapped to the hg19 assembly using Tophat with mapping percentages greater than 50%. Sample reads were assembled into transcript models using Cufflinks, which were then merged and run through Cuffdiff to produce per sample FPKM expression levels and estimate differential expression between the three treatments compared with media. Treatment responses were estimated as the ratio between the expression level for a treatment condition to the expression level for the media sample. To estimate differential treatment response, we did a pairwise comparison for each transcript as the difference in treatment responses. From this we calculated a maximum difference metric as the differential treatment response with the largest absolute value: genes were ranked by the maximum difference (MaxDiff) between the three treatment responses (hIL7, hGMCSF+IL7 and hGIFT7) and selected the 560 genes where MaxDiff was at least 1 in a log2 scale, which represents at least a twofold differential response. To organize the MaxDiff genes for effective visualization and to segregate into groups based on expression patterns, we performed hierarchical clustering, with Euclidean distance and Pearson dissimilary metrics for genes and samples, respectively. Of note, although each treatment group used a single biological replicate, the observations reported in Figure 1 were validated in a parallel experiment using microarrays (data not shown).

### Flow cytometry and tetramer staining

Cells to be analyzed by fluorescence-activated cell sorter were harvested and resuspended in 10 × 10^6^ cells ml^−1^. Samples were blocked with anti-FcR mAb 2.4G2 for 30 min and subsequently stained with fluorochrome-conjugated monoclonal antibody in Ca^2+^Mg^2+^-free PBS with 2% fetal bovine serum for 30 min. Anti-mCMV-specific T cells were counted using an allophycocyanin-conjugated HGIRNASFI-H-2D^b^ tetramer.^28, 59^ Cells were washed twice with staining buffer and resuspended in 1% PFA before analysis.

For intracellular Ki67 staining, cultured thymocytes were fixed and permeabilized with Cytofix/Cytoperm buffer (BD Bioscience, San Jose, CA, USA) at 4 °C for 20 min. Cells were then incubated in 0.5% Tween-20/1% PFA buffer at room temperature for 30 min. All intracellular cytokine staining was performed with Cytofix/Cytoperm Kit (BD Bioscience) according to the manufacturer's instruction. All fluorescence-activated cell sorter antibodies were purchased from BD Pharmingen (San Jose, CA, USA).

### GIFT7 administration and mCMV infection

In all, 5 μg kg^−1^ of GIFT7 was administered i.p. into aged mice (C57Bl/6 RB) every other day for a total of seven injections. Recombinant mGMCSF and mIL7 (R&D System) were used as cytokine controls. Mice were infected i.p. with 1 × 10^5^ (or 5 × 10^4^ for athymic animals) plaque-forming unit salivary gland passed Smith strain MCMV (a gift from Dr Mohammad S Hossain, Emory University, USA) 7 days after the last GIFT7 (or control cytokine) injection. Spleens were obtained from infected 3, 7 or 21 days post viral inoculation for total and mCMV-specific T-cell analysis.

### TREC analysis

Splenic DNA was isolated using DNA purification kit (Qiagen). Real-time quantitative PCR was performed in triplicates on an ABI 7500 Fast Real-Time PCR system thermal cycler (Life Technologies, Grand Island, NY, USA) with 15 μl volume, containing 100 ng of sample DNA, 500nM of forward and reverse primers, SYBR Green Mastermix (Applied Biosystems, Grand Island, NY, USA). The forward and reverse TREC primers were 5′-CCAAGCTGACGGCAGGTTT-3′ and 3′-AGCATGGCAAGCAGCACC-5′, respectively; the forward and reverse primers for TCRα were 5′-TGACTCCCAAATCAATGTG-3′ and 5′-GCAGGTGAAGCTTGTCTG-3′, respectively. The relative expression of sjTREC over TCRα was expressed as 2^−ΔCT^ with ΔCT being defined as (CT of sjTREC−CT of TCRα). Fold difference is expressed as the relative mRNA expression normalized against the average relative expression of sjTREC over TCRα in the untreated group.

### Histological analysis

Thymi were fixed, embedded and cut for standard H&E staining. Slides were examined and scored in a blind manner. At least, three thymi sections for each group were examined.

### Statistical analysis

*P*-values were calculated by paired Student's *t*-test (Excel) and significance was defined as *P*<0.05. Data are reported as mean±s.d. as indicated.
