# Supplementary figures and images for: A GMCSF and IL7 fusion cytokine leads to functional thymic-dependent T-cell regeneration in age-associated immune deficiency
Source: Clin Transl Immunology. 2015 May 8;4(5):e37–. doi: 10.1038/cti.2015.8 (PMC4478872; doi:10.1038/cti.2015.8)

**A.**

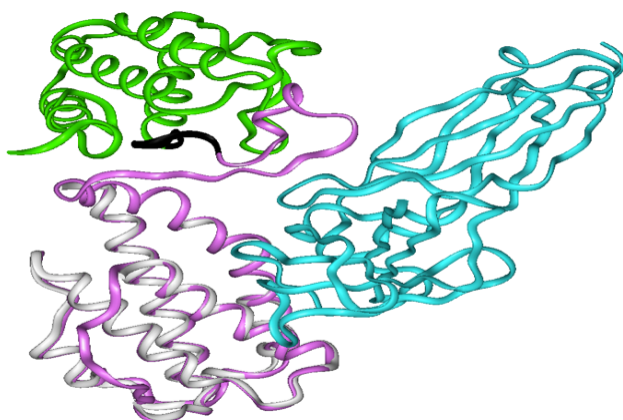

**B.**

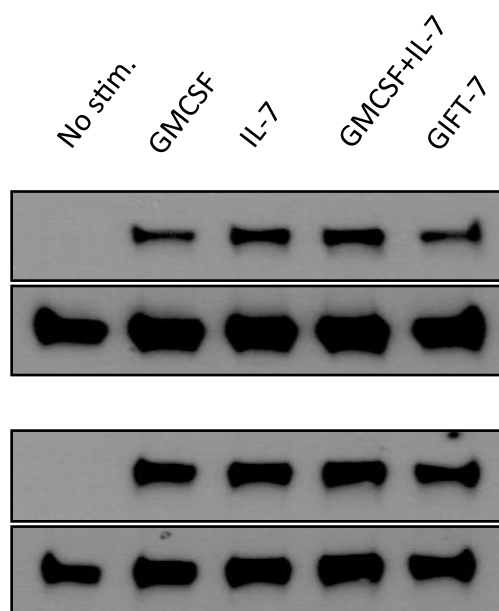

**A.**

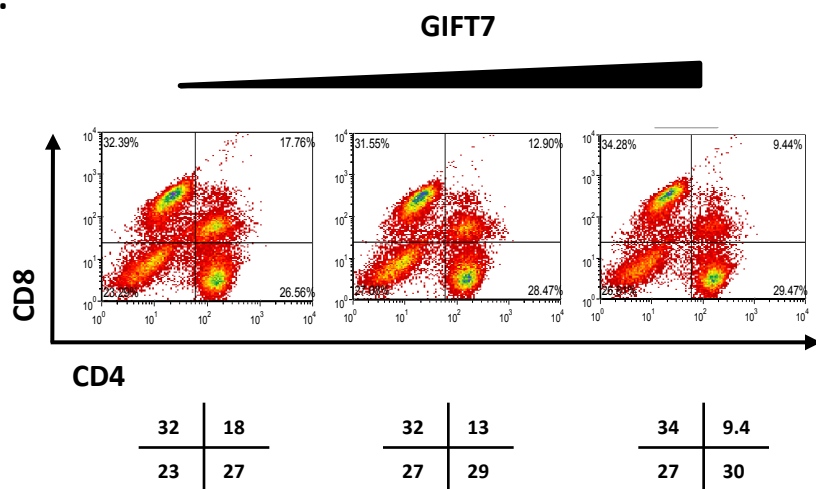

**B.**

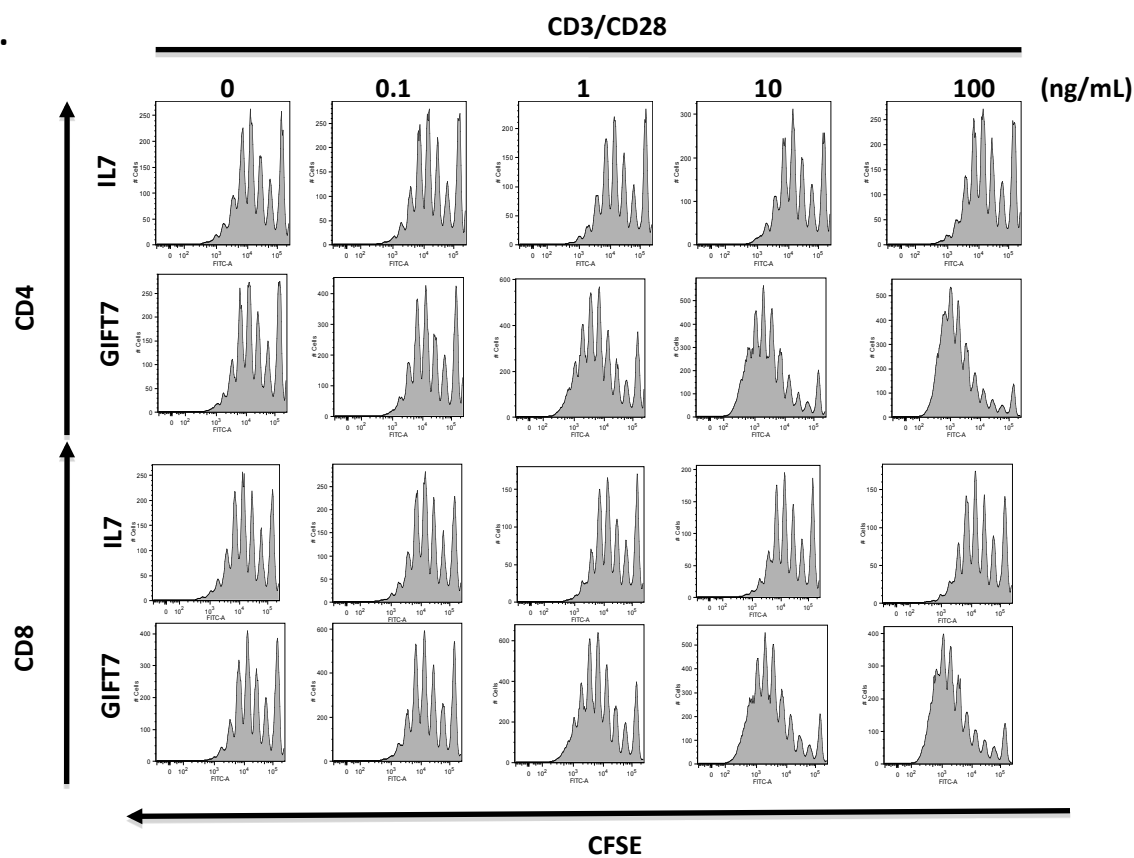

**A.**

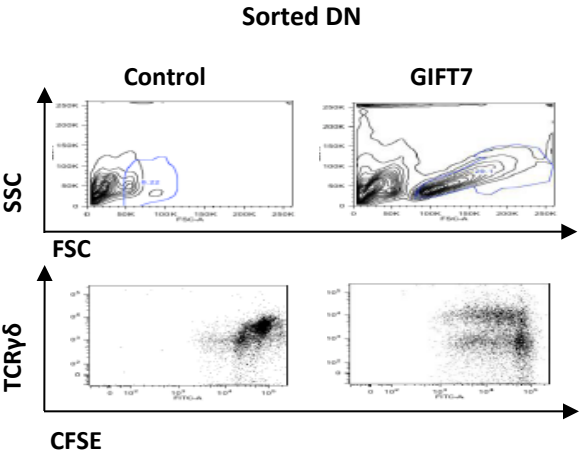

**B.**

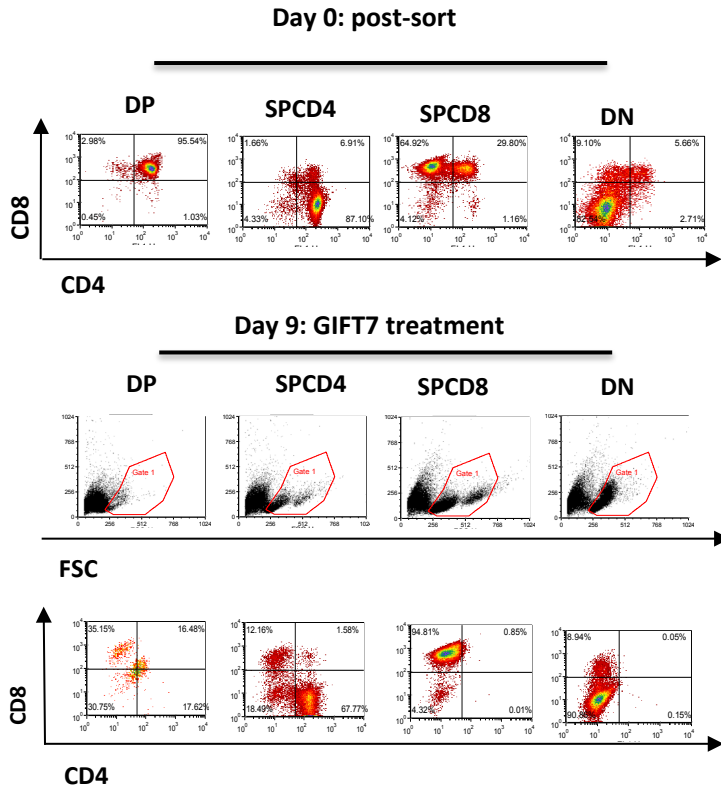

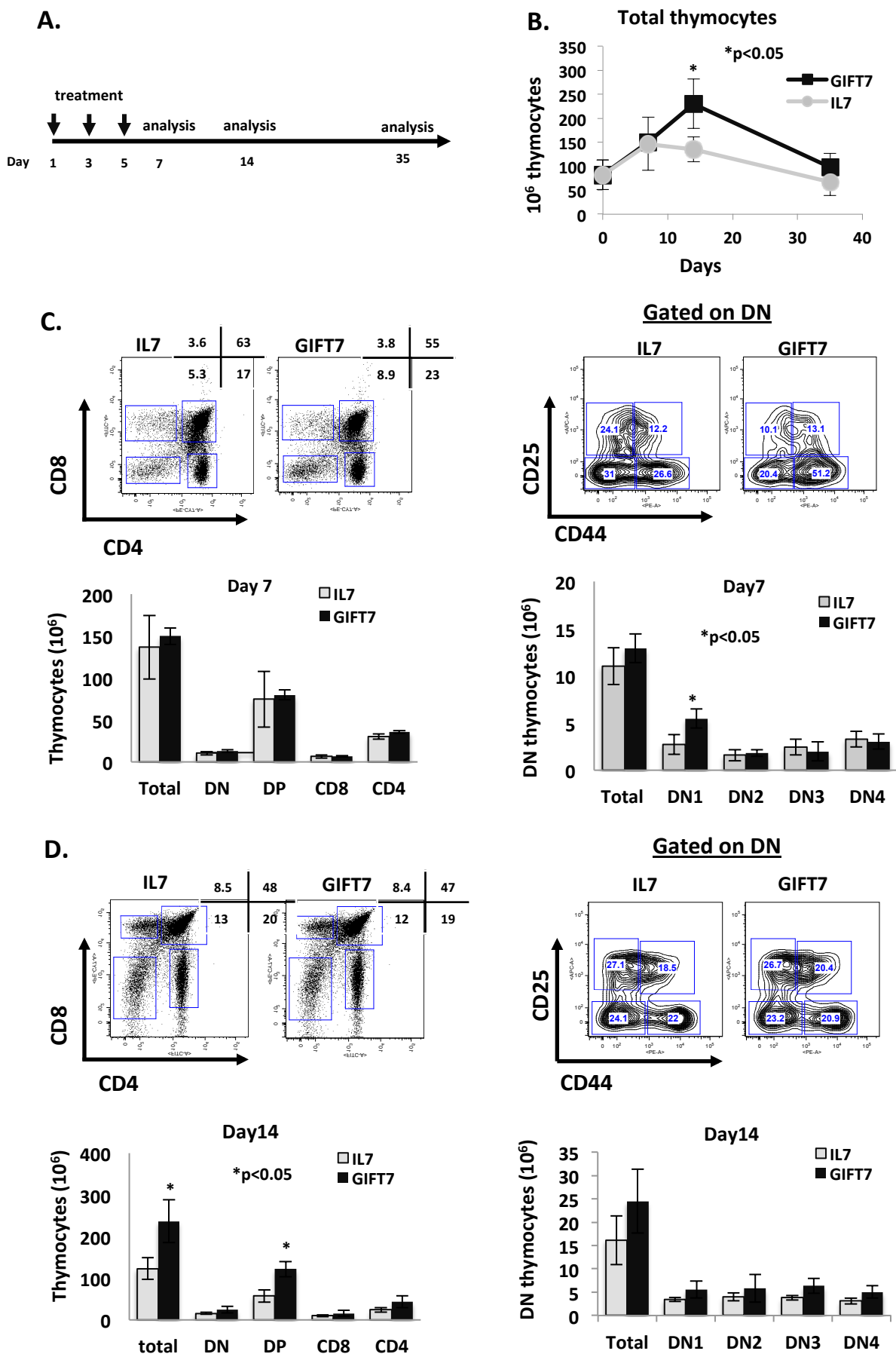

Supplemental figure 4

**A.**

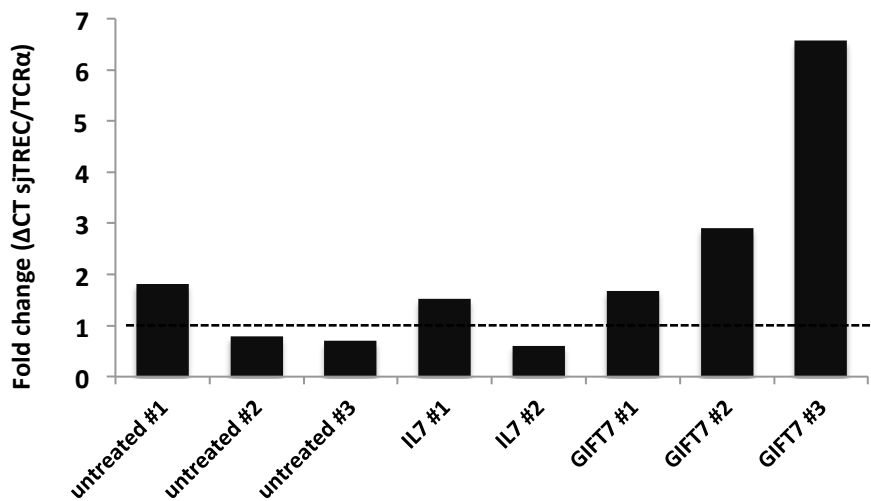

**B.**

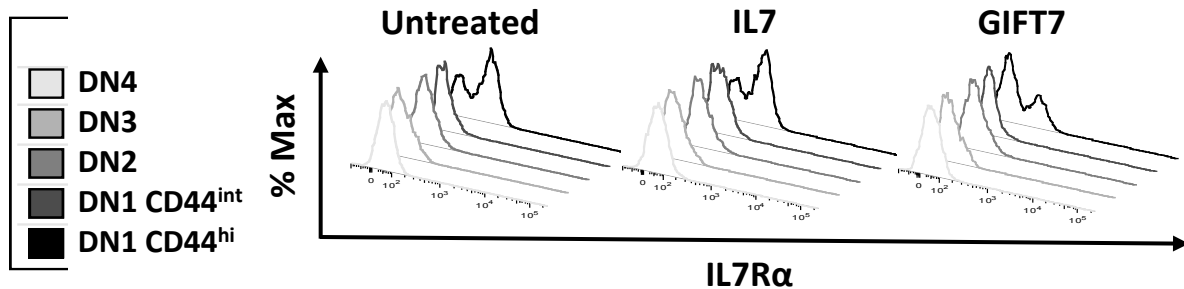

**C.**

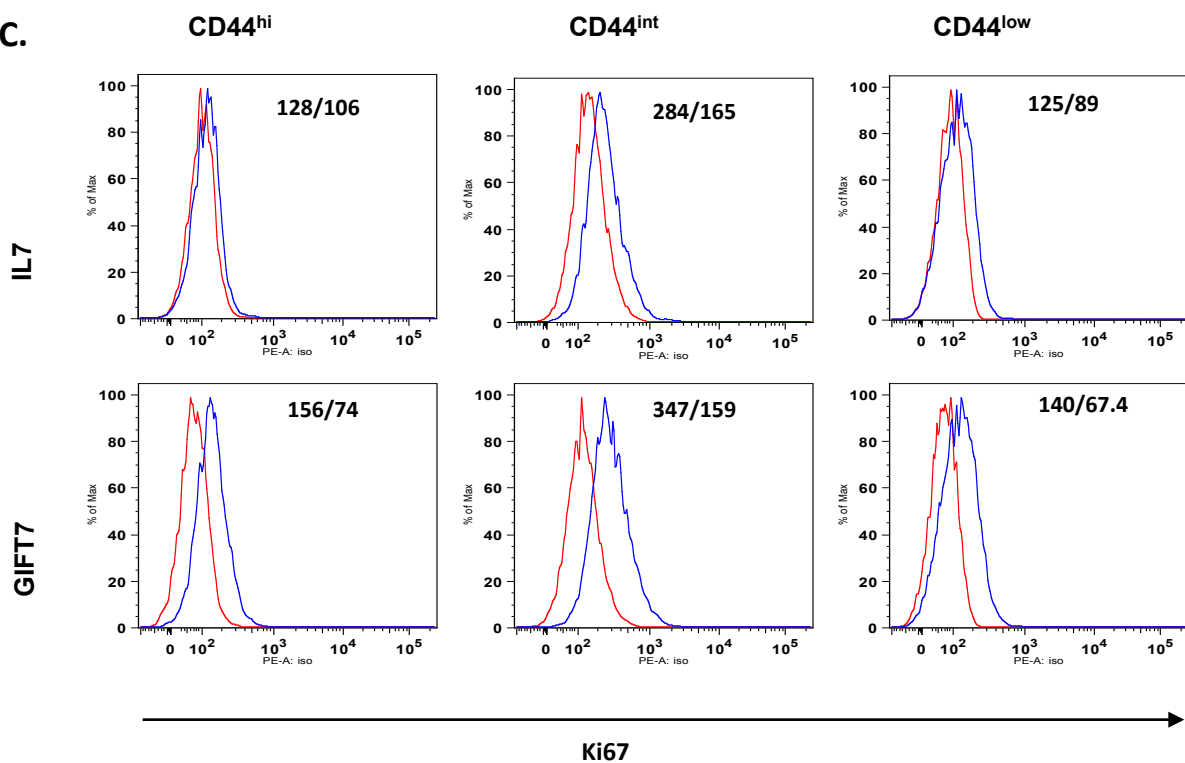

A.

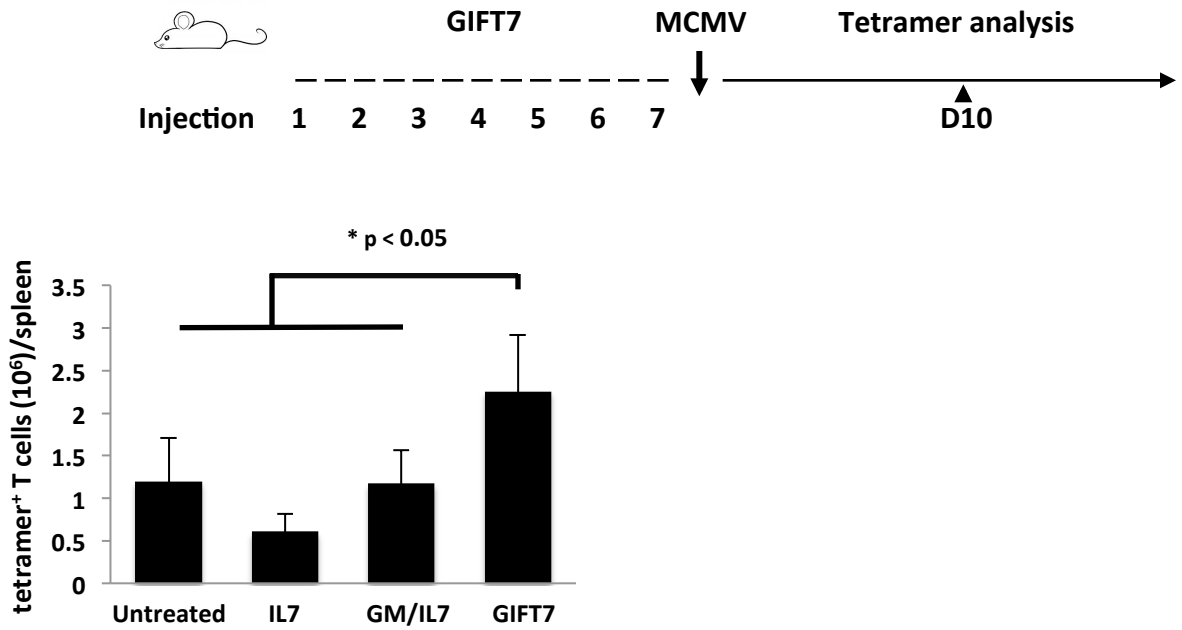

B.

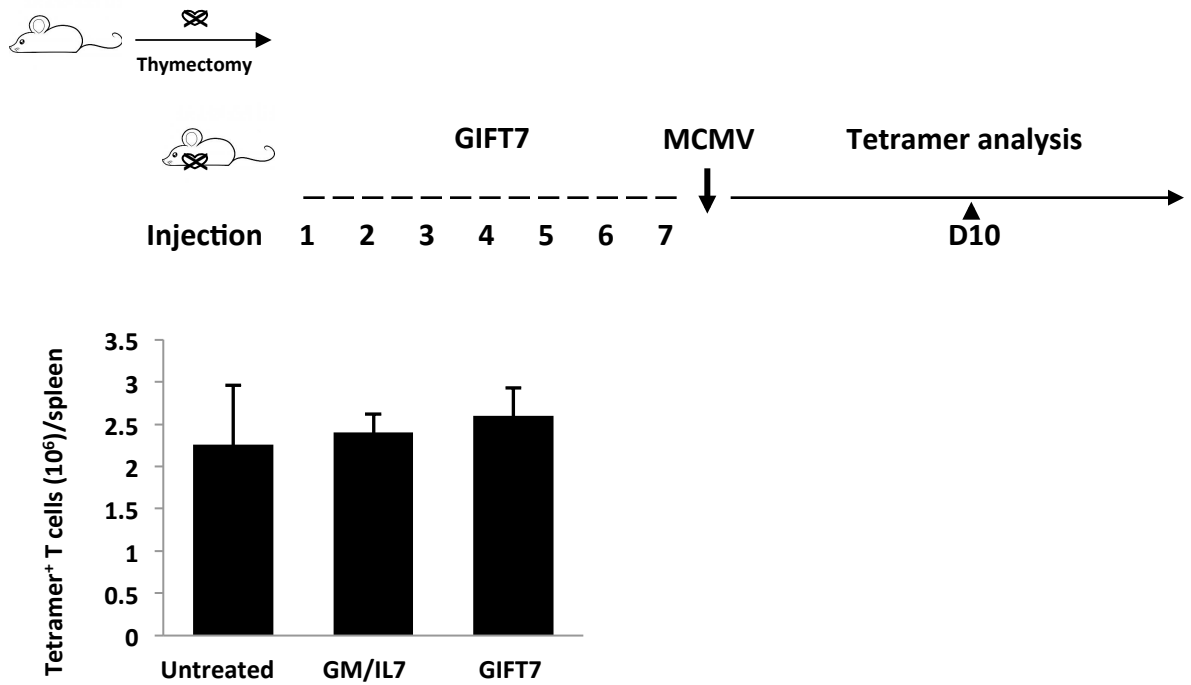

Supplement: Supplementary Figures [file cti20158x1.pdf]
