# Supplementary material for: A GMCSF and IL7 fusion cytokine leads to functional thymic-dependent T-cell regeneration in age-associated immune deficiency
Source: Clin Transl Immunology. 2015 May 8;4(5):e37–. doi: 10.1038/cti.2015.8 (PMC4478872; doi:10.1038/cti.2015.8)
Supplement: Supplementary Figure Legends [file cti20158x2.doc]

**Figure S1. Biochemical characterization of GIFT7** (A) The GIFT7 structural model aligned with the crystal structure of IL7 (white ribbon) in complex with IL7 receptor alpha (cyan ribbon). (B) Western blotting for phospho-specific and total STAT3 and STAT5 proteins from cell lysates of 3 × 10^5^ primary, naïve T-cells after stimulation with 1 nM GIFT-7 or cytokine controls for 15 minutes.

**Figure S2. GIFT7 treatment on primary thymocytes and PBMC**

(A) Thymocytes were isolated from 6-8 week old mice and cultured in the presence of GIFT7 at increasing concentration (10ngmL^-1^, 50ngmL^-1^, 100ngmL^-1^). Re-analysis of CD4/CD8 expression on cultured thymocytes at day 5. (B) Histogram indicates flow cytometry analysis of CFSE dilution of hPBMC 3 days after stimulation with CD3/CD28-coated beads in the presence of hGIFT7 or hIL7 at the indicated concentration.

**Figure S3. GIFT7 leads to the proliferation but not differentiation of pre-sorted DN cells**

(A) Carboxyfluorescein diacetate succinimidyl ester (CFSE) dilution of sorted DN thymocytes after cultured with GIFT7 (10ng mL^-1^) for 5 days. Cells are stained with TCRγδ before analysis. SSC, side scatter. FSC, forward scatter. (B) GIFT7 does not lead to the differenEaEon of pre-selected thymocytes. Dissociated thymi were stained for CD4 and CD8. FACS-sorted DN, SPCD4, SPCD8, and DP were subsequently cultured in GIFT7 (10ng/ml) for 9 days. Surface expression of CD4 and CD8 were reanalyzed. Flow cytometry of one representative experiment.

**Figure S4: GIFT7 leads to transient thymic hyperplasia in young mice**

**(A)** 2-month-old C57Bl/6 were injected with three doses of GIFT7 or IL7 i.v. (5ug/Kg) at 1-day interval. Thymi collected on day 7, 14, or 35 were analyzed for total and subset cellularity. **(B)** Total thymic cellularity in GIFT7 or IL7-treated groups at each time point. Results represent the mean cell number +/- SD (n = 3-5); **P*<0.05 **(C, D)** Dissociated thymi were analyzed for CD3, CD4, CD8, CD25, and CD44 expression. Dot plots from one representative animal indicate the frequency of each thymic subset on day 7 in **(C)** and day 14 in **(D)**. The histograms represent the number of cells associated with each phenotype. Data represent mean +/-SD (n = 5) *p<0.05.

**Figure S5: the biological effect of GIFT7 on thymocytes in vivo and in vitro**

(A) GIFT7 administration leads to increased thymic output detected in the periphery of 2/3 treated mice as measured by **sjTREC in splenic T cells**. mRNA expression of single-joint (sj) TREC to TCRα ratio of splenocytes from aged mice were measured by RT-PCR. Histogram represents fold difference of the relative mRNA expression (2^-ΔCT of sjTREC to TCRα^) from each treated mice normalized to the mean relative mRNA expression from the untreated group. RT-PCR was performed in triplicates; (B) IL7Rα is primarily expressed in the DN1 CD44^hi^ subset. Thymi derived from GIFT7-, IL7-, or untreated aged mice were dissociated and analyzed by surface expression of CD4, CD8, CD25, CD44, and CD127 (IL7Rα) by flow cytometry. Histogram represents IL7Rα expression in different subsets of DN thymocytes. **(C)** Naïve RB-derived, magnetically purified DN cells were cultured with IL7 or GIFT7 (20ngmL^-1^) for 5 days. Histogram plot of Ki67 expression over CD44^hi^, CD44^int^, and CD44^low^ subsets shows proliferative cells in the 3 different populations. Numbers in histogram plot indicate mean fluorescence intensity (MFI) of Ki67 staining over isotype staining

**Figure S6: GIFT7 treatment leads to an augmentation of anti-viral CTL response in thymic-intact mice**

**(A)** Schematic representation indicates 7 i.p. injections of GIFT7 or cytokine controls at 5ug/kg in thymectomized RB mice. 5 x 10^4^ PFU of MCMV were injected 7 days after the last cytokine injection. Spleens were analyzed for total or viral-specific cellularity at day 10 post-viral infection by mMCV peptide MHC tetramer staining. **(B)** Absolute numbers of mCMV tetramer+ CD8+ T cells were quantified based on flow cytometry analysis. Histogram shows mean cellularity per spleen +/- SDV (n = 4).
