# Supplementary material for: A GMCSF and IL7 fusion cytokine leads to functional thymic-dependent T-cell regeneration in age-associated immune deficiency
Source: Clin Transl Immunology. 2015 May 8;4(5):e37–. doi: 10.1038/cti.2015.8 (PMC4478872; doi:10.1038/cti.2015.8)
Supplement: Supplementary Table 1 [file cti20158x3.pdf]

Cluster	RefSeq Gene	NCBI Gene Sy	MaxDiff	IL7 / media	IL7+GM / me	GIFT7 / medi	NCBI Gene ID	LocusTag	Synonyms	dbXrefs	chromosome	map_locatio	description	type_of_gen	Symbol_from	Full_name_fr	Nomenclatur	Other_design	Modification_date
1	AGSK1	?	1.31917	1.23671	0.893751	-0.0824614	|nd|	|nd|	|nd|	|nd|	|nd|	|nd|	|nd|	|nd|	|nd|	|nd|	|nd|	|nd|	|nd|
1	AGSK1	?	1.12178	1.41452	1.12412	0.292741	|nd|	|nd|	|nd|	|nd|	|nd|	|nd|	|nd|	|nd|	|nd|	|nd|	|nd|	|nd|	|nd|
1	AGSK1	?	1.10375	1.2488	0.981767	0.145048	|nd|	|nd|	|nd|	|nd|	|nd|	|nd|	|nd|	|nd|	|nd|	|nd|	|nd|	|nd|	|nd|
3	LOC285359	?	1.38676	-1.71544	-0.838288	-2.22505	|nd|	|nd|	|nd|	|nd|	|nd|	|nd|	|nd|	|nd|	|nd|	|nd|	|nd|	|nd|	|nd|
3	ZNF487P	?	1.05867	-1.429	-0.370331	-0.540218	|nd|	|nd|	|nd|	|nd|	|nd|	|nd|	|nd|	|nd|	|nd|	|nd|	|nd|	|nd|	|nd|
2	RRP7B	RRP7B	1.05207	-0.652633	-0.152108	0.399434	91695	-	dJ222E13.2	HGNC:30454	22	22q13.2	ribosomal RN	pseudo	RRP7B	ribosomal RN	O	-	20130226
2	LOC90784	LOC90784	1.04038	-0.414713	-0.0537356	0.625662	90784	-	-	-	2	2p11.2	uncharacteri	miscRNA	-	-	-	-	20130226
2	HIST1H2AG	HIST1H2AG	1.00514	-0.264737	0.733356	0.740405	8969	-	H2A.1b|H2A	HGNC:4737|	6	6p22.1	histone clust	protein-codir	HIST1H2AG	histone clust	O	H2A histone	20130804
2	HIST1H4E	HIST1H4E	1.03655	1.20943	0.934774	1.97132	8367	-	H4/j|H4FJ	HGNC:4790|	6	6p22.1	histone clust	protein-codir	HIST1H4E	histone clust	O	H4 histone fa	20130804
4	HIST1H2BG	HIST1H2BG	1.83207	-0.99671	-2.79572	-0.96365	8339	-	H2B.1A|H2B	HGNC:4746|	6	6p21.3	histone clust	protein-codir	HIST1H2BG	histone clust	O	H2B histone	20130804
3	HIST1H4I	HIST1H4I	2.23365	-1.60785	0.625799	-0.618422	8294	-	H4/m|H4FM	HGNC:4793|	6	6p21.33	histone clust	protein-codir	HIST1H4I	histone clust	O	H4 histone fa	20130804
1	NEIL1	NEIL1	1.46686	2.22705	1.70318	0.760192	79661	-	FPG1|NEI1|I	HGNC:18448	15	15q24.2	nei endonucl	protein-codir	NEIL1	nei endonucl	O	DNA glycosyl	20130804
4	SNHG9	SNHG9	2.13323	-0.257188	-2.13723	-0.0039953	735301	-	NCRNA0006	HGNC:33102	16	16p13.3	small nucleol	miscRNA	SNHG9	small nucleol	O	-	20130216
2	FAM72A	FAM72A	1.14642	1.51645	2.13073	2.66287	729533	RP11-312O7.	LMPIP	HGNC:24044	1	1q32.1	family with s	protein-codir	FAM72A	family with s	O	LMP1-induce	20130804
1	MIR590	MIR590	1.83781	1.807	0.747423	-0.0308141	693175	-	MIRN590|hs	HGNC:32846	7	7q11.23	microRNA 59	miscRNA	MIR590	microRNA 59	O	-	20130807
3	SNORD94	SNORD94	1.93004	-2.05402	-0.370019	-2.30006	692225	-	U94	HGNC:32756	2	2p11.2	small nucleol	snoRNA	SNORD94	small nucleol	O	-	20130216
1	SNORD97	SNORD97	1.52699	-0.47622	-0.574664	-2.00321	692223	-	U97	HGNC:32760	11	11p15.3	small nucleol	snoRNA	SNORD97	small nucleol	O	-	20130216
3	SCARNA10	SCARNA10	2.55061	-2.18245	0.368161	-1.18952	692148	-	U85	HGNC:32567	12	12p13.31	small Cajal b	miscRNA	SCARNA10	small Cajal b	O	-	20130216
3	SNORA25	SNORA25	2.30661	-2.8089	-0.502291	-0.754812	684959	-	ACA25	HGNC:32615	11	11q21	small nucleol	snoRNA	SNORA25	small nucleol	O	-	20130216
3	SNORA55	SNORA55	1.88303	-0.527737	1.11682	-0.766208	677834	-	ACA55	HGNC:32649	1	1p34.3	small nucleol	snoRNA	SNORA55	small nucleol	O	-	20130216
4	SNORA53	SNORA53	1.26465	0.344297	-0.371726	0.892924	677832	-	ACA53	HGNC:32646	12	12q23.1	small nucleol	snoRNA	SNORA53	small nucleol	O	-	20130216
1	SNORA51	SNORA51	1.98622	1.82571	2.22254	0.236322	677831	-	ACA51	HGNC:32644	20	20p13	small nucleol	snoRNA	SNORA51	small nucleol	O	-	20130216
3	SNORA44	SNORA44	2.03818	-3.31098	-1.2728	-2.40312	677825	-	ACA44	HGNC:32637	1	1p35.3	small nucleol	snoRNA	SNORA44	small nucleol	O	-	20130216
3	SNORA40	SNORA40	1.17707	-0.456379	0.720688	0.501319	677822	-	ACA40	HGNC:32633	11	11q21	small nucleol	snoRNA	SNORA40	small nucleol	O	-	20130430
3	SNORA31	SNORA31	2.34553	-1.06212	1.28341	-0.771362	677814	-	ACA31	HGNC:32621	13	13q14.13	small nucleol	snoRNA	SNORA31	small nucleol	O	-	20130430
3	SNORA29	SNORA29	1.22694	-0.488226	0.738719	-0.128103	677812	-	ACA29	HGNC:32619	6	6q25.3	small nucleol	snoRNA	SNORA29	small nucleol	O	-	20130430
3	SNORA26	SNORA26	2.39554	-3.46396	-1.06842	-1.4813	677810	-	HBI-6	HGNC:32616	4	4q12	small nucleol	snoRNA	SNORA26	small nucleol	O	-	20130216
1	SNORA18	SNORA18	1.40366	-0.106462	-0.101949	-1.50561	677805	-	ACA18	HGNC:32608	11	11q21	small nucleol	snoRNA	SNORA18	small nucleol	O	-	20130216
1	SNORA12	SNORA12	1.20245	-0.485351	-0.918428	-1.6878	677800	-	U108	HGNC:32600	10	10q24.31	small nucleol	snoRNA	SNORA12	small nucleol	O	-	20130804
3	SNORA9	SNORA9	2.4888	-0.495263	1.99354	0.80908	677798	-	ACA9	HGNC:32597	7	7p13	small nucleol	snoRNA	SNORA9	small nucleol	O	-	20130216
3	SNORA5C	SNORA5C	2.2531	0.426144	2.67924	1.54394	677796	-	ACA5c	HGNC:32590	7	7p13	small nucleol	snoRNA	SNORA5C	small nucleol	O	-	20130216
3	SNORA1	SNORA1	2.33737	-1.45547	0.881902	-0.161238	677792	-	ACA1	HGNC:32557	11	11q21	small nucleol	snoRNA	SNORA1	small nucleol	O	-	20130216
3	SCARNA15	SCARNA15	1.80172	-1.46271	0.339013	-0.746577	677778	-	ACA45	HGNC:32572	15	15q25.2	small Cajal b	miscRNA	SCARNA15	small Cajal b	O	-	20130216
1	SCARNA6	SCARNA6	2.34011	1.69472	1.21931	-0.645393	677772	-	U88	HGNC:32562	2	2q37.1	small Cajal b	miscRNA	SCARNA6	small Cajal b	O	-	20130216
1	FSCN1	FSCN1	1.5115	3.64239	3.48236	2.13089	6624	-	FAN1|HSN|S	HGNC:11148	7	7p22	fascin homol	protein-codir	FSCN1	fascin homol	O	55 kDa actin-	20130804
3	SNORA8	SNORA8	1.05139	0.520875	1.57226	0.687778	654320	-	ACA8	HGNC:32596	11	11q21	small nucleol	snoRNA	SNORA8	small nucleol	O	-	20130216
2	FAM72B	FAM72B	1.17949	1.95958	2.8563	3.13907	653820	RP11-439A17	p17	HGNC:24805	1	1p11.2	family with s	protein-codir	FAM72B	family with s	O	amyloid-beta	20130804
1	FAM153C	FAM153C	3.7926	4.04484	3.43428	0.252244	653316	-	NY-REN-7-lik	HGNC:33936	5	5q35.3	family with s	pseudo	FAM153C	family with s	O	-	20130216
4	SNORA48	SNORA48	1.29049	-1.45612	-2.74661	-1.69266	652965	-	ACA48	HGNC:32641	17	17p13.1	small nucleol	snoRNA	SNORA48	small nucleol	O	-	20130216
1	LOC646471	LOC646471	1.02829	0.879804	0.778427	-0.148483	646471	-	-	-	1	1p36.11	uncharacteri	miscRNA	-	-	-	-	20130216
2	CCNI2	CCNI2	1.27906	-0.145509	0.619995	1.13355	645121	-	-	HGNC:33869	5	5q31.1	cyclin I family	protein-codir	CCNI2	cyclin I family	O	cyclin-I2	20130307
1	LOC642423	LOC642423	1.21141	2.05621	2.46934	1.25793	642423	-	-	-	15	15q25.3	golgin A2 pse	pseudo	-	-	-	-	20130508
3	SNORA4	SNORA4	2.01615	-2.04967	-0.0335196	-0.673798	619568	-	ACA4	HGNC:32587	3	3q27	small nucleol	snoRNA	SNORA4	small nucleol	O	-	20130216
1	BCYRN1	BCYRN1	1.67349	-1.95746	-2.15825	-3.63095	618	-	BC200|BC20	HGNC:1022|	2	2p21	brain cytopla	miscRNA	BCYRN1	brain cytopla	O	-	20130226
2	SNORA62	SNORA62	2.55685	0.4875	2.36343	3.04435	6044	-	E2|E2-1|RNE	HGNC:10107	3	3p22.1	small nucleol	snoRNA	SNORA62	small nucleol	O	-	20130628
1	RMRP	RMRP	1.6035	2.86595	2.98335	1.37985	6023	-	CHH|NME1|	HGNC:10031	9	9p21-p12	RNA compon	miscRNA	RMRP	RNA compon	O	-	20130804
3	SNORA33	SNORA33	1.13591	1.07787	2.21378	1.26769	594839	-	ACA33	HGNC:32623	6	6q23.2	small nucleol	snoRNA	SNORA33	small nucleol	O	-	20130216
3	SNORA6	SNORA6	1.17099	0.639481	1.76775	0.596762	574040	-	ACA6	HGNC:32591	3	3p22.2	small nucleol	snoRNA	SNORA6	small nucleol	O	-	20130523
1	ARL17A	ARL17A	1.28583	-1.03041	-1.56648	-2.31624	51326	-	ARF1P2|ARL	HGNC:24096	17	17q21.31	ADP-ribosyla	protein-codir	ARL17A	ADP-ribosyla	O	ADP-ribosyla	20130804
1	MT1L	MT1L	1.21113	2.2727	1.97725	1.06157	4500	-	MT1|MT1R|	HGNC:7404|	16	16q13	metallothion	pseudo	MT1L	metallothion	O	-	20130226
1	GOLGA6L9	GOLGA6L9	1.00307	0.717992	0.600296	-0.285074	440295	-	-	HGNC:37229	15	15q25.2	golgin A6 fan	protein-codir	GOLGA6L9	golgin A6 fan	O	Golgin subfar	20130518
2	MIR22	MIR22	1.4286	-1.90046	-1.35593	-0.471856	407004	-	MIRN22|hsa	HGNC:31599	17	17p13.3	microRNA 22	miscRNA	MIR22	microRNA 22	O	-	20130728
2	MIR17	MIR17	1.09289	-0.415855	0.582151	0.677035	406952	-	MIR17-5p|M	HGNC:31547	13	13q31.3	microRNA 17	miscRNA	MIR17	microRNA 17	O	-	20130804
2	OR2A20P	OR2A20P	1.08295	-1.24126	-0.753256	-0.158312	401428	-	OR2A20	HGNC:15413	7	7q35	olfactory rec	pseudo	OR2A20P	olfactory rec	O	-	20130430
2	OR2A20P	OR2A20P	1.08295	-1.24126	-0.753256	-0.158312	401428	-	OR2A20	HGNC:15413	7	7q35	olfactory rec	pseudo	OR2A20P	olfactory rec	O	-	20130430
1	DKFZP686I15	LINC01011	1.03524	0.438084	0.053874	-0.597152	401232	-	DKFZP686I15	HGNC:33812	6	6p25.2	long interger	miscRNA	LINC01011	long interger	O	-	20130727
1	LOC399715	LOC399715	1.24529	0.455059	0.403439	-0.790228	399715	-	-	Ensembl:ENS	10	10p15.1	uncharacteri	miscRNA	-	-	-	-	20130226
3	HERC2P10	HERC2P10	1.00334	-1.63458	-0.63124	-0.804584	390561	-	-	HGNC:39056	15	15q13.2	hect domain	pseudo	HERC2P10	hect domain	O	-	20130226
1	OR52N4	OR52N4	1.18566	0.0855865	-0.737027	-1.10007	390072	-	OR11-64	HGNC:15230	11	11p15.4	olfactory rec	protein-codir	OR52N4	olfactory rec	O	olfactory rec	20130720
1	LOC388152	LOC388152	1.53175	2.02239	1.88718	0.490643	388152	-	-	Ensembl:ENS	15	15q25.2	golgin A2 pse	pseudo	-	-	-	-	20130623
1	ZNF429	ZNF429	1.16804	-0.381164	-0.321132	-1.48917	353088	-	-	HGNC:20817	19	19p13.1	zinc finger pr	protein-codir	ZNF429	zinc finger pr	O	-	20130804
2	MT1DP	MT1DP	1.07438	0.808757	0.61798	1.69236	326343	-	MTM	HGNC:7396|	16	16q13	metallothion	pseudo	MT1DP	metallothion	O	-	20130804
1	EMR4P	EMR4P	1.17643	-0.396272	-0.568884	-1.5727	326342	hCG_181145	EMR4|FIRE|(	HGNC:19240	19	19p13.3	egf-like modu	pseudo	EMR4P	egf-like modu	O	-	20130226
3	HIST1H2AD	HIST1H2AD	1.87266	0.0890389	1.9617	0.555821	3013	-	H2A.3|H2A/g	HGNC:4729|	6	6p21.3	histone clust	protein-codir	HIST1H2AD	histone clust	O	H2A histone	20130804
3	PRICKLE4	PRICKLE4	1.1328	0.197605	0.81695	-0.315851	29964	RP11-298J23	C6orf49|OBT	HGNC:16805	6	6p21.31	prickle homo	protein-codir	PRICKLE4	prickle homo	O	over-express	20130226
1	FAM86B3P	FAM86B3P	1.27581	-0.76434	-0.631823	-1.90763	286042	hCG_199054	-	HGNC:44371	8	8p23.1	family with s	pseudo	FAM86B3P	family with s	O	-	20130804
1	LINC00663	LINC00663	1.66234	-0.437432	-0.612797	-2.09977	284440	-	-	HGNC:28609	19	19p13.11	long interger	miscRNA	LINC00663	long interger	O	-	20130804
2	FUOM	FUOM	1.27634	0.170339	-0.0670363	1.2093	282969	RP11-122K13	C10orf125|F	HGNC:24733	10	10q26.3	fucose mutar	protein-codir	FUOM	fucose mutar	O	protein fucU	20130226
1	PALD1	PALD1	1.69954	6.99629	6.78436	5.29675	27143	RP11-710A11	KIAA1274|PA	HGNC:23530	10	10q22.1	phosphatase	protein-codir	PALD1	phosphatase	O	paladin|palla	20130804
3	SNORA67	SNORA67	1.36803	-0.961513	0.406516	-0.612865	26781	-	RNU67|U67	HGNC:10224	17	17p13.1	small nucleol	snoRNA	SNORA67	small nucleol	O	-	20130804
2	LRWD1	LRWD1	1.5423	-1.3755	-0.676729	0.1668	222229	-	CENP-33|OR	HGNC:21769	7	7q22.1	leucine-rich r	protein-codir	LRWD1	leucine-rich r	O	ORC-associat	20130804
2	ABHD11-AS1	LINC00035	2.59039	-2.20207	-0.405771	0.388318	171022	-	ABHD11-AS1	HGNC:18289	7	7q11.23	long interger	miscRNA	LINC00035	long interger	O	-	20130226
1	LOC143666	LOC143666	1.29906	1.50331	1.27116	0.204252	143666	-	-	-	11	11p15.5	uncharacteri	miscRNA	-	-	-	-	20130226
2	RPS4Y2	RPS4Y2	1.0916	3.4422	3.6575	4.5338	140032	-	RPS4Y2P	HGNC:18501	Y	Yq11.223	ribosomal pr	protein-codir	RPS4Y2	ribosomal pr	O	40S ribosoma	20130804
1	ADORA2B	ADORA2B	2.24316	5.88617	5.27085	3.64301	136	-	ADORA2	HGNC:264|N	17	17p12	adenosine A2	protein-codir	ADORA2B	adenosine A2	O	adenosine re	20130804
2	HIST3H2BB	HIST3H2BB	1.03598	0.923392	1.85143	1.95937	128312	-	H2Bb	HGNC:20514	1	1q42.13	histone clust	protein-codir	HIST3H2BB	histone clust	O	H2B type 12|	20130804
1	HIST4H4	HIST4H4	1.94667	-0.594627	0.0934783	-1.85319	121504	-	H4/p	HGNC:20510	12	12p12.3	histone clust	protein-codir	HIST4H4	histone clust	O	histone 4, H4	20130804
1	GPR146	GPR146	1.22026	1.34317	1.18391	0.122915	115330	-	PGR8	HGNC:21718	7	7p22.3	G protein-co	protein-codir	GPR146	G protein-co	O	G protein-co	20130226
1	MST1P2	MST1P2	1.71735	0.803657	0.108242	-0.913693	11209	-	MSPL-1|MSF	HGNC:7383|	1	1p36.2	macrophage	pseudo	MST1P2	macrophage	O	-	20130717
1	SPON2	SPON2	1.09246	0.145865	-0.410182	-0.946599	10417	UNQ435/PRO	DIL-1|DIL1|N	HGNC:11253	4	4p16.3	spondin 2, ex	protein-codir	SPON2	spondin 2, ex	O	differentially	20130606
4	LINC00659	LINC00659	2.82863	-0.0610216	-2.87046	-0.0418283	100652730	-	-	HGNC:44316	20	-	long interger	miscRNA	LINC00659	long interger	O	-	20130220
3	MIR5047	MIR5047	1.38683	-1.43164	-0.420493	-1.80732	100616408	-	-	HGNC:41834	17	-	microRNA 50	miscRNA	MIR5047	microRNA 50	O	-	20130216
1	LOC1005073	LOC1005073	2.97777	-0.012467	-0.0885464	-2.99024	100507387	-	-	Ensembl:ENS	5	-	uncharacteri	miscRNA	-	-	-	-	20130217
1	MIR3916	MIR3916	1.04085	0.172867	-0.125252	-0.867986	100500849	-	-	HGNC:38912	1	-	microRNA 39	miscRNA	MIR3916	microRNA 39	O	-	20130217
1	BACE1-AS	BACE1-AS	1.32538	-0.353774	-0.0880811	-1.41346	100379571	-	BACE1-AS1|E	HGNC:37125	11	11q23.3	BACE1 antise	miscRNA	BACE1-AS	BACE1 antise	O	-	20130217
3	LOC1002888	LOC1002888	1.56275	-1.03032	0.53243	-0.736183	100288842	-	-	-	9	9q33.2	UDP-GlcNAc:	pseudo	-	-	-	-	20130724
1	LOC1002331	LOC1002331	1.13148	-0.146396	-0.847969	-1.27788	100233156	-	-	-	4	4p16.3	tektin 4 pseu	pseudo	-	-	-	-	20130220
1	UBE2Q2P2	UBE2Q2P2	1.2221	0.615341	0.500246	-0.606758	100134869	-	UBE2QP2	HGNC:37440	15	15q25	ubiquitin-cor	pseudo	UBE2Q2P2	ubiquitin-cor	O	-	20130217
1	UBE2Q2P2	UBE2Q2P2	1.22077	0.614291	0.499463	-0.606481	100134869	-	UBE2QP2	HGNC:37440	15	15q25	ubiquitin-cor	pseudo	UBE2Q2P2	ubiquitin-cor	O	-	20130217
4	PHKA2-AS1	PHKA2-AS1	1.15919	0.287899	-0.871288	-0.207658	100132163	-	-	HGNC:44110	X	Xp22.13	PHKA2 antise	miscRNA	PHKA2-AS1	PHKA2 antise	O	-	20130217
2	LOC1001321	LOC1001321	1.84004	-2.07095	-1.63201	-0.230914	100132111	-	-	-	1	1q21.3	uncharacteri	miscRNA	-	-	-	-	20130217
4	LOC1001316	LOC1001316	1.24334	-0.760381	-1.81695	-0.573615	100131626	-	-	-	11	11q23.3	uncharacteri	miscRNA	-	-	-	-	20130217
2	LOC1001310	LOC1001310	1.06475	-0.946302	-0.0513582	0.11845	100131094	-	-	-	19	19p13.3	uncharacteri	protein-codir	-	-	-	uncharacteri	20130217
1	C19orf71	C19orf71	1.03722	-0.11027	-0.767011	-1.14749	100128569	-	-	HGNC:34496	19	19p13.3	chromosome	protein-codir	C19orf71	chromosome	O	Uncharacteri	20130217
1	SCARNA27	SCARNA27	2.32758	1.52759	0.295857	-0.799988	100124533	-	-	HGNC:33614	6	6p24.3	small Cajal b	miscRNA	SCARNA27	small Cajal b	O	-	20130220
3	SNORD58C	SNORD58C	2.40029	1.33744	2.73609	0.335799	100124516	-	U58	HGNC:33613	18	18q21.1	small nucleol	snoRNA	SNORD58C	small nucleol	O	-	20130220
2	CDC20	CDC20	1.55528	1.46253	2.21249	3.01781	991	-	CDC20A|bA2	HGNC:1723|	1	1p34.1	cell division c	protein-codir	CDC20	cell division c	O	CDC20 cell di	20130804
2	URB2	URB2	1.07306	-0.476148	-0.195675	0.596909	9816	RP11-108F13	KIAA0133|NI	HGNC:28967	1	1q42.13	URB2 riboson	protein-codir	URB2	URB2 riboson	O	nucleolar pre	20130804
1	TMEM63A	TMEM63A	1.45603	-0.407402	-0.738219	-1.86343	9725	RP4-559A3.1	KIAA0792	HGNC:29118	1	1q42.12	transmembra	protein-codir	TMEM63A	transmembra	O	-	20130804
1	CD72	CD72	1.59398	1.60638	1.62859	0.034609	971	RP11-331F9.!	CD72b|LYB2	HGNC:1696|	9	9p13.3	CD72 molecu	protein-codir	CD72	CD72 molecu	O	B-cell differe	20130804
2	CD70	CD70	1.24098	-0.653773	-0.354504	0.587211	970	-	CD27L|CD27	HGNC:11937	19	19p13	CD70 molecu	protein-codir	CD70	CD70 molecu	O	CD27 ligand|	20130804
1	ZNF516	ZNF516	1.114	0.167488	-0.282718	-0.946515	9658	-	HsT287	HGNC:28990	18	18q23	zinc finger pr	protein-codir	ZNF516	zinc finger pr	O	-	20130804
1	PLCH2	PLCH2	1.59287	0.918008	0.63537	-0.674858	9651	RP3-395M20	PLC-eta2|PL(	HGNC:29037	1	1p36.32	phospholipas	protein-codir	PLCH2	phospholipas	O	1-phosphatic	20130804
2	TNFRSF8	TNFRSF8	1.19346	3.94926	4.37123	5.14272	943	-	CD30|D1S16	HGNC:11923	1	1p36	tumor necros	protein-codir	TNFRSF8	tumor necros	O	CD30L recept	20130804
2	CD86	CD86	1.48258	0.635938	0.33105	1.81363	942	-	B7-2|B7.2|B	HGNC:1705|	3	3q21	CD86 molecu	protein-codir	CD86	CD86 molecu	O	B-lymphocyt	20130804
1	ARHGAP12	ARHGAP12	1.06193	-0.0745449	-0.540846	-1.13647	94134	-	-	HGNC:16348	10	10p11.22	Rho GTPase a	protein-codir	ARHGAP12	Rho GTPase a	O	rho GTPase-a	20130804
1	SYTL3	SYTL3	1.22315	2.09399	1.82307	0.870839	94120	-	SLP3	HGNC:15587	6	6q25.3	synaptotagm	protein-codir	SYTL3	synaptotagm	O	exophilin-6|s	20130804
2	CD80	CD80	1.12312	2.02856	2.41465	3.15168	941	-	B7|B7-1|B7.	HGNC:1700|	3	3q13.3-q21	CD80 molecu	protein-codir	CD80	CD80 molecu	O	B-lymphocyt	20130804
2	TTYH2	TTYH2	1.02423	-2.54064	-1.69146	-1.51641	94015	-	C17orf29	HGNC:13877	17	17q25.1	tweety homo	protein-codir	TTYH2	tweety homo	O	hTTY2|prote	20130804
2	ARMC6	ARMC6	1.04622	-0.397732	-0.286574	0.64849	93436	-	R30923_1	HGNC:25049	19	19p13.11	armadillo rep	protein-codir	ARMC6	armadillo rep	O	armadillo rep	20130804
1	NREP	NREP	1.38636	3.944	3.68774	2.55764	9315	-	C5orf13|D4S	HGNC:16834	5	5q22.1	neuronal reg	protein-codir	NREP	neuronal reg	O	neuronal pro	20130804
1	OLFM2	OLFM2	1.59931	1.67911	1.18594	0.0798006	93145	-	NOE2|NOELI	HGNC:17189	19	19p13.2	olfactomedir	protein-codir	OLFM2	olfactomedir	O	neuronal olfa	20130804
2	MARS2	MARS2	1.00456	-1.01194	-0.725816	-0.0073758	92935	-	MetRS|mtM	HGNC:25133	2	2q33.1	methionyl-tR	protein-codir	MARS2	methionyl-tR	O	methionine t	20130804
1	HIST3H2A	HIST3H2A	1.12305	1.27024	0.79455	0.147194	92815	-	-	HGNC:20507	1	1q42.13	histone clust	protein-codir	HIST3H2A	histone clust	O	histone 3, H2	20130804
2	WDR46	WDR46	1.01422	-0.412054	0.00091961	0.602162	9277	DADB-159G1	BING4|C6orf	HGNC:13923	6	6p21.3	WD repeat d	protein-codir	WDR46	WD repeat d	O	WD repeat-c	20130804
2	MSC	MSC	1.28902	-1.13405	-1.05747	0.154965	9242	-	ABF-1|ABF1|	HGNC:7321|	8	8q21	musculin	protein-codir	MSC	musculin	O	activated B-c	20130804
1	NOG	NOG	3.16971	0.335239	-0.175732	-2.83447	9241	-	SYM1|SYNS1	HGNC:7866|	17	17q22	noggin	protein-codir	NOG	noggin	O	symphalangis	20130804
2	C17orf72	C17orf72	1.02082	0.359866	0.663236	1.38069	92340	-	-	HGNC:25673	17	17q23.3	chromosome	protein-codir	C17orf72	chromosome	O	uncharacteri	20130724

1	ZC3HAV1L	ZC3HAV1L	1.30857	2.5052	2.6622	1.35363	92092	-	C7orf39	HGNC:22423	7	7q34	zinc finger C(	protein-codir	ZC3HAV1L	zinc finger C(	O	zinc finger C(	20130804
2	IDI2	IDI2	1.07384	-0.731777	0.0269258	0.342063	91734	-	IPPI2	HGNC:23487	10	10p15.3	isopentenyl-(	protein-codir	IDI2	isopentenyl-(	O	IPP isomeras	20130806
1	PLXNA4	PLXNA4	1.4943	6.5174	6.26692	5.0231	91584	tcag7.1291	FAYV2820|PI	HGNC:9102|	7	7q32.3	plexin A4	protein-codir	PLXNA4	plexin A4	O	plexin-A4	20130804
2	RRP9	RRP9	1.1065	-0.427815	-0.433519	0.672977	9136	-	RNU3IP2|U3	HGNC:16829	3	3p21.2	ribosomal RN	protein-codir	RRP9	ribosomal RN	O	RNA, U3 sma	20130804
2	CCNB2	CCNB2	1.15059	1.93112	2.40123	3.08171	9133	-	HsT17299	HGNC:1580|	15	15q22.2	cyclin B2	protein-codir	CCNB2	cyclin B2	O	G2/mitotic-s|	20130804
1	SCARF2	SCARF2	1.41102	0.169647	-0.359113	-1.24137	91179	-	NSR1|SREC-I	HGNC:19869	22	22q11.21	scavenger re	protein-codir	SCARF2	scavenger re	O	scavenger re	20130804
1	PPP1R3E	PPP1R3E	1.7297	1.17846	0.916886	-0.551242	90673	-	-	HGNC:14943	14	14q11.2	protein phos	protein-codir	PPP1R3E	protein phos	O	protein phos	20130804
1	N4BP2L1	N4BP2L1	1.17946	0.384962	0.236023	-0.794499	90634	RP11-298P3.	CG018	HGNC:25037	13	13q13.1	NEDD4 bindi	protein-codir	N4BP2L1	NEDD4 bindi	O	NEDD4-bindi	20130226
2	C15orf23	KNSTRN	1.01262	1.72126	2.10807	2.73388	90417	HSD11	C15orf23|SK	HGNC:30767	15	15q15.1	kinetochore-	protein-codir	KNSTRN	kinetochore-	O	TRAF4 associ	20130804
1	ZNF160	ZNF160	1.08831	1.16937	0.988359	0.0810582	90338	-	F11|HKr18|H	HGNC:12948	19	19q13.42	zinc finger pr	protein-codir	ZNF160	zinc finger pr	O	KRAB zinc fin	20130804
2	TSPAN18	TSPAN18	1.07203	1.12566	1.813	2.19769	90139	UNQ3042/PF	TSPAN	HGNC:20660	11	11p11.2	tetraspanin 1	protein-codir	TSPAN18	tetraspanin 1	O	tetraspanin-1	20130226
1	CCNG2	CCNG2	1.16032	0.333702	0.0938053	-0.826614	901	-	-	HGNC:1593|	4	4q21.1	cyclin G2	protein-codir	CCNG2	cyclin G2	O	cyclin-G2	20130804
1	NOL3	NOL3	1.63556	6.14819	5.75316	4.51263	8996	-	ARC|FCM|M	HGNC:7869|	16	16q22.1	nucleolar prc	protein-codir	NOL3	nucleolar prc	O	muscle-enric	20130804
1	LHX4	LHX4	1.53786	-1.35535	-0.974617	-2.51248	89884	-	CPHD4	HGNC:21734	1	1q25.2	LIM homeob	protein-codir	LHX4	LIM homeob	O	LIM homeob	20130804
1	P4HA2	P4HA2	1.70978	6.2181	6.25305	4.54327	8974	UNQ290/PR(	-	HGNC:8547|	5	5q31	prolyl 4-hydr	protein-codir	P4HA2	prolyl 4-hydr	O	4-PH alpha 2	20130804
1	HERC3	HERC3	1.18901	0.938786	0.668423	-0.25022	8916	-	-	HGNC:4876|	4	4q21	HECT and RLI	protein-codir	HERC3	HECT and RLI	O	HECT domair	20130804
1	CACNA1I	CACNA1I	1.873	-1.26535	-1.67621	-3.13835	8911	RP1-172B20.	Cav3.3|ca(v)	HGNC:1396|	22	22q13.1	calcium chan	protein-codir	CACNA1I	calcium chan	O	calcium chan	20130804
2	CCNB1	CCNB1	1.24866	1.76239	2.36348	3.01105	891	-	CCNB	HGNC:1579|	5	5q12	cyclin B1	protein-codir	CCNB1	cyclin B1	O	G2/mitotic-s|	20130804
1	SYNGAP1	SYNGAP1	6.77154	6.512	0.205163	-0.259538	8831	DASS-97D12.	MRD5|RASA	HGNC:11497	6	6p21.3	synaptic Ras	protein-codir	SYNGAP1	synaptic Ras	O	Ras GTPase-a	20130804
1	SYNGAP1	SYNGAP1	6.76043	6.54344	0.217866	-0.216992	8831	DASS-97D12.	MRD5|RASA	HGNC:11497	6	6p21.3	synaptic Ras	protein-codir	SYNGAP1	synaptic Ras	O	Ras GTPase-a	20130804
1	TNFRSF10D	TNFRSF10D	1.55504	1.79617	1.48727	0.241126	8793	UNQ251/PR(	CD264|DCR2	HGNC:11907	8	8p21	tumor necros	protein-codir	TNFRSF10D	tumor necros	O	TNF receptor	20130804
1	TNK1	TNK1	1.19583	-0.351749	-0.603456	-1.54758	8711	-	-	HGNC:11940	17	17p13.1	tyrosine kina	protein-codir	TNK1	tyrosine kina	O	CD38 negativ	20130804
1	DNAH11	DNAH11	1.09534	2.51034	2.19187	1.415	8701	-	CILD7|DNAH	HGNC:2942|	7	7p21	dynein, axon	protein-codir	DNAH11	dynein, axon	O	axonemal be	20130804
2	RUVBL1	RUVBL1	1.14285	-0.434884	-0.0090128	0.707968	8607	-	ECP54|INO8(	HGNC:10474	3	3q21	RuvB-like 1 (I	protein-codir	RUVBL1	RuvB-like 1 (I	O	49 kDa TATA	20130804
1	DIXDC1	DIXDC1	1.03513	2.41023	2.1907	1.3751	85458	-	CCD1	HGNC:23695	11	11q23.1	DIX domain c	protein-codir	DIXDC1	DIX domain c	O	coiled-coil pr	20130804
2	TMEM241	TMEM241	1.11681	0.33411	0.612384	1.45092	85019	-	C18orf45|hV	HGNC:31723	18	18q11.2	transmembra	protein-codir	TMEM241	transmembra	O	putative vert	20130804
2	AGPAT9	AGPAT9	1.84506	-0.176053	0.535697	1.66901	84803	HMFN0839	AGPAT8|GPA	HGNC:28157	4	4q21.23	1-acylglycero	protein-codir	AGPAT9	1-acylglycero	O	1-AGP acyltra	20130804
2	MPV17L2	MPV17L2	2.03867	-1.34918	-0.357397	0.689491	84769	-	FKSG24	HGNC:28177	19	19p13.11	MPV17 mito(	protein-codir	MPV17L2	MPV17 mito(	O	mpv17-like p	20130804
1	CBX2	CBX2	1.09328	3.28899	3.73081	2.63753	84733	-	CDCA6|M33	HGNC:1552|	17	17q25.3	chromobox h	protein-codir	CBX2	chromobox h	O	Pc class hom	20130804
2	PSRC1	PSRC1	1.06742	2.30897	2.97674	3.37639	84722	RP11-297O4.	DDA3|FP321	HGNC:24472	1	1p13.3	proline/serin	protein-codir	PSRC1	proline/serin	O	differential d	20130804
1	KISS1R	KISS1R	2.42117	3.59752	3.08085	1.17635	84634	-	AXOR12|CPF	HGNC:4510|	19	19p13.3	KISS1 recept(	protein-codir	KISS1R	KISS1 recept(	O	G protein-co	20130804
1	NTNG2	NTNG2	1.6766	-0.106884	-0.39168	-1.78348	84628	RP11-479K2(	LHLL9381|Ln	HGNC:14288	9	9q34	netrin G2	protein-codir	NTNG2	netrin G2	O	bA479K20.1	20130804
2	MAP1LC3A	MAP1LC3A	1.88088	-2.57624	-1.53516	-0.695359	84557	RP11-346K17	ATG8E|LC3|I	HGNC:6838|	20	20q11.22	microtubule-	protein-codir	MAP1LC3A	microtubule-	O	MAP1 light cl	20130804
1	BRSK1	BRSK1	1.06557	1.99342	1.75044	0.927846	84446	-	hSAD1	HGNC:18994	19	19q13.4	BR serine/thr	protein-codir	BRSK1	BR serine/thr	O	BR serine/thr	20130804
2	PDCD2L	PDCD2L	1.38121	-1.21528	-0.978615	0.165928	84306	-	-	HGNC:28194	19	19q13.11	programmed	protein-codir	PDCD2L	programmed	O	programmed	20130804
2	SLC25A33	SLC25A33	1.14619	-0.0421734	0.368034	1.10402	84275	-	BMSC-MCP|I	HGNC:29681	1	1p36.22	solute carrier	protein-codir	SLC25A33	solute carrier	O	PNC1 proteir	20130804
1	TMEM191A	TMEM191A	1.15377	0.146545	-0.0186928	-1.00723	84222	-	TMEM191AP	HGNC:25317	22	22q11.21	transmembra	pseudo	TMEM191A	transmembra	O	-	20130226
2	TOMM40L	TOMM40L	1.08947	-0.572278	-0.159957	0.517193	84134	-	RP11-297K8.	HGNC:25756	1	1q23.3	translocase c	protein-codir	TOMM40L	translocase c	O	mitochondria	20130804
1	OBSCN	OBSCN	1.30911	-0.1817	-0.553088	-1.49081	84033	RP5-1139B12	ARHGEF30|U	HGNC:15719	1	1q42.13	obscurin, cyt	protein-codir	OBSCN	obscurin, cyt	O	obscurin|obs	20130804
1	TMPRSS13	TMPRSS13	1.6495	-0.684203	0.121463	-1.52804	84000	-	MSP|MSPL|I	HGNC:29808	11	11q23	transmembra	protein-codir	TMPRSS13	transmembra	O	membrane-t	20130804
1	RASSF4	RASSF4	1.02545	-0.097786	-1.06582	-1.12324	83937	RP11-285G1.	AD037	HGNC:20793	10	10q11.21	Ras associati	protein-codir	RASSF4	Ras associati	O	Ras associati	20130804
1	FGFBP2	FGFBP2	2.27248	1.58977	1.1227	-0.682709	83888	UNQ425/PR(	HBP17RP|KS	HGNC:29451	4	4p16	fibroblast grc	protein-codir	FGFBP2	fibroblast grc	O	37 kDa killer-	20130804
2	ATAD3B	ATAD3B	1.20513	-0.437686	-0.036923	0.767443	83858	RP4-628J24.3	AAA-TOB3|T	HGNC:24007	1	1p36.33	ATPase famil	protein-codir	ATAD3B	ATPase famil	O	AAA-ATPase	20130804
3	ROPN1L	ROPN1L	1.56755	2.79872	4.36627	3.80162	83853	-	ASP|RSPH11	HGNC:24060	5	5p15.2	rhophilin ass	protein-codir	ROPN1L	rhophilin ass	O	AKAP-associa	20130804
2	GRWD1	GRWD1	1.14568	-0.801069	-0.505818	0.34461	83743	-	CDW4|GRWI	HGNC:21270	19	19q13.33	glutamate-ric	protein-codir	GRWD1	glutamate-ric	O	CUL4- and DI	20130804
1	JAM3	JAM3	2.66112	3.57853	3.0573	0.917412	83700	UNQ859/PR(	JAM-2|JAM-3	HGNC:15532	11	11q25	junctional ad	protein-codir	JAM3	junctional ad	O	junctional ad	20130804
2	HIST1H3H	HIST1H3H	1.33011	-0.637664	0.130745	0.692447	8357	-	H3/k|H3F1K	HGNC:4775|	6	6p22.1	histone clust	protein-codir	HIST1H3H	histone clust	O	H3 histone fa	20130804
4	HIST1H3E	HIST1H3E	1.14234	0.696152	-0.446191	-0.0444507	8353	RP1-34B20.8	H3.1|H3/d|H	HGNC:4769|	6	6p22.1	histone clust	protein-codir	HIST1H3E	histone clust	O	H3 histone fa	20130804
2	EMC6	EMC6	1.72487	-0.388756	-0.686585	1.03828	83460	-	TMEM93	HGNC:28430	17	17p13.2	ER membran	protein-codir	EMC6	ER membran	O	transmembra	20130714
1	SBF2	SBF2	1.62611	1.63778	1.19983	0.0116686	81846	-	CMT4B2|DEI	HGNC:2135|	11	11p15.4	SET binding f	protein-codir	SBF2	SET binding f	O	DENN/MADD	20130804
2	NETO2	NETO2	1.06479	0.164881	0.572747	1.22967	81831	UNQ1926/PF	BTCL2|NEOT	HGNC:14644	16	16q11	neuropilin (N	protein-codir	NETO2	neuropilin (N	O	brain-specific	20130804
2	SLC7A5	SLC7A5	1.00971	-0.0944932	0.11797	0.915213	8140	-	4F2LC|CD98	HGNC:11063	16	16q24.3	solute carrier	protein-codir	SLC7A5	solute carrier	O	4F2 LC|4F2 li	20130804
1	PAR5	PWAR5	1.10451	-0.606535	-1.32978	-1.71105	8123	-	D15S226E|PA	HGNC:30090	15	15q11.2	Prader Willi/A	miscRNA	PWAR5	Prader Willi/A	O	-	20130618
1	PRR7	PRR7	1.88871	3.2516	2.66621	1.36289	80758	-	-	HGNC:28130	5	5q35.3	proline rich 7	protein-codir	PRR7	proline rich 7	O	proline-rich p	20130804
2	TSEN2	TSEN2	1.0052	-1.05121	-0.8982	-0.0460103	80746	-	PCH2B|SEN2	HGNC:28422	3	3p25.2	TSEN2 tRNA s	protein-codir	TSEN2	TSEN2 tRNA s	O	hsSen2|tRNA	20130807
2	FOSL1	FOSL1	1.11349	3.45422	3.35634	4.46983	8061	-	FRA|FRA1|fr	HGNC:13718	11	11q13	FOS-like anti	protein-codir	FOSL1	FOS-like anti	O	FOS-like anti	20130804
2	PUS1	PUS1	1.43481	-0.900183	-0.231954	0.534627	80324	PP8985	MLASA1	HGNC:15508	12	12q24.33	pseudouridyl	protein-codir	PUS1	pseudouridyl	O	mitochondria	20130804
1	GKAP1	GKAP1	1.05971	-0.332399	-0.513378	-1.39211	80318	FKSG21	GKAP42	HGNC:17496	9	9q21.32	G kinase ancl	protein-codir	GKAP1	G kinase ancl	O	G kinase-anc	20130804
1	LRRC27	LRRC27	1.114	0.0681098	-0.171656	-1.04589	80313	RP11-273H7.	-	HGNC:29346	10	10q26.3	leucine rich r	protein-codir	LRRC27	leucine rich r	O	leucine-rich r	20130430
2	C16orf59	C16orf59	1.02294	3.27573	3.85478	4.29867	80178	-	-	HGNC:25849	16	16p13.3	chromosome	protein-codir	C16orf59	chromosome	O	uncharacteri	20130804
2	PIF1	PIF1	1.11359	1.64175	2.34877	2.75534	80119	-	C15orf20|PIF	HGNC:26220	15	15q22.31	PIF1 5'-to-3' I	protein-codir	PIF1	PIF1 5'-to-3' I	O	ATP-depende	20130709
4	GPR157	GPR157	1.14493	0.448409	-0.696523	-0.636545	80045	RP5-963K15.	-	HGNC:23687	1	1p36.23	G protein-co	protein-codir	GPR157	G protein-co	O	probable G-p	20130226
1	UBTD1	UBTD1	1.05048	-0.161732	-0.7778	-1.21221	80019	-	-	HGNC:25683	10	10q24.2	ubiquitin dor	protein-codir	UBTD1	ubiquitin dor	O	ubiquitin dor	20130804
1	C10orf95	C10orf95	1.49353	-0.732537	-0.476658	-1.97019	79946	RP11-18I14.6	-	HGNC:25880	10	10q24.32	chromosome	protein-codir	C10orf95	chromosome	O	uncharacteri	20130226
1	DOK3	DOK3	1.33233	2.07884	2.43268	1.10035	79930	-	DOKL	HGNC:24583	5	5q35.3	docking prot	protein-codir	DOK3	docking prot	O	Dok-like prot	20130804
2	UBXN8	UBXN8	1.36203	-0.264478	0.449624	1.09755	7993	-	D8S2298E|RI	HGNC:30307	8	8p12-p11.2	UBX domain	protein-codir	UBXN8	UBX domain	O	Reproductior	20130804
1	MORN1	MORN1	1.0551	-0.104151	-0.583737	-1.15925	79906	RP4-740C4.1	-	HGNC:25852	1	1p36.33-p36	MORN repea	protein-codir	MORN1	MORN repea	O	MORN repea	20130804
1	HDAC11	HDAC11	1.41785	-0.0745976	-0.202966	-1.49245	79885	-	HD11	HGNC:19086	3	3p25.1	histone deac	protein-codir	HDAC11	histone deac	O	-	20130804
2	TREML2	TREML2	1.16403	2.48959	2.79562	3.65362	79865	UNQ6268/PF	C6orf76|TLT-	HGNC:21092	6	6p21.1	triggering rec	protein-codir	TREML2	triggering rec	O	trem-like trar	20130226
1	FAM57A	FAM57A	1.26026	3.98687	3.86147	2.72661	79850	-	CT120	HGNC:29646	17	17p13.3	family with s	protein-codir	FAM57A	family with s	O	membrane p	20130804
2	AGMAT	AGMAT	1.04515	-0.810167	-0.618133	0.234986	79814	-	-	HGNC:18407	1	1p36.21	agmatine ure	protein-codir	AGMAT	agmatine ure	O	AUH|agmatir	20130804
2	ISOC2	ISOC2	1.28835	-0.84963	-0.0058014	0.438725	79763	-	-	HGNC:26278	19	19q13.42	isochorismat	protein-codir	ISOC2	isochorismat	O	isochorismat	20130804
1	ASB13	ASB13	1.04561	0.681211	-0.134067	-0.364397	79754	-	-	HGNC:19765	10	10p15.1	ankyrin repea	protein-codir	ASB13	ankyrin repea	O	ankyrin repea	20130804
1	SH3D21	SH3D21	1.67948	5.06446	4.84594	3.38498	79729	RP11-268J15	C1orf113	HGNC:26236	1	1p34.3	SH3 domain (	protein-codir	SH3D21	SH3 domain (	O	SH3 domain-	20130226
2	YRDC	YRDC	1.04531	-0.350624	-0.117355	0.694686	79693	-	DRIP3|IRIP|F	HGNC:28905	1	1p34.3	yrdC domain	protein-codir	YRDC	yrdC domain	O	dopamine re	20130804
2	ZDHHC14	ZDHHC14	1.09651	-1.28966	-0.941484	-0.193147	79683	RP3-481C9.1	NEW1CP	HGNC:20341	6	6q25.3	zinc finger, D	protein-codir	ZDHHC14	zinc finger, D	O	DHHC-14|NE	20130804
1	PPP1R3B	PPP1R3B	1.65972	1.69865	1.44711	0.0389343	79660	-	GL|PPP1R4|I	HGNC:14942	8	8p23.1	protein phos	protein-codir	PPP1R3B	protein phos	O	PP1 subunit I	20130804
1	BEND5	BEND5	2.3544	4.04706	4.10224	1.74784	79656	-	C1orf165	HGNC:25668	1	1p33	BEN domain	protein-codir	BEND5	BEN domain	O	BEN domain-	20130804
2	AIMP2	AIMP2	1.46633	0.395379	1.12527	1.86171	7965	PRO0992	JTV-1|JTV1|F	HGNC:20609	7	7p22	aminoacyl tR	protein-codir	AIMP2	aminoacyl tR	O	ARS-interacti	20130804
3	C1orf54	C1orf54	1.23176	-1.859	-0.627235	-1.33523	79630	RP4-790G17.	-	HGNC:26258	1	1q21.2	chromosome	protein-codir	C1orf54	chromosome	O	uncharacteri	20130226
2	FAM118B	FAM118B	1.05603	-0.686569	-0.637982	0.369463	79607	-	-	HGNC:26110	11	11q24.2	family with s	protein-codir	FAM118B	family with s	O	protein FAM	20130804
2	SLC52A2	SLC52A2	1.1735	-0.560688	-0.135014	0.612813	79581	-	BVVLS2|D15	HGNC:30224	8	8q24.3	solute carrier	protein-codir	SLC52A2	solute carrier	O	G protein-co	20130718
3	RSG1	RSG1	1.03431	-0.282901	0.33408	-0.700227	79363	RP4-733M16	C1orf89	HGNC:28127	1	1p36.13	REM2 and RA	protein-codir	RSG1	REM2 and RA	O	REM2- and R	20130804
2	CCDC86	CCDC86	1.09948	0.0221857	0.501377	1.12167	79080	-	-	HGNC:28359	11	11q12.2	coiled-coil dc	protein-codir	CCDC86	coiled-coil dc	O	coiled-coil dc	20130804
2	METRN	METRN	1.11234	-0.898063	-0.503505	0.214282	79006	RJD6	C16orf23|c3	HGNC:14151	16	16p13.3	meteorin, gli	protein-codir	METRN	meteorin, gli	O	meteorin	20130226
1	ZNF204P	ZNF204P	1.04934	-0.265143	-0.804062	-1.31448	7754	-	ZNF184-Lp|Z	HGNC:12995	6	6p21.3	zinc finger pr	pseudo	ZNF204P	zinc finger pr	O	-	20130226
1	ZBTB25	ZBTB25	1.19412	0.387732	0.254204	-0.806384	7597	-	C14orf51|KU	HGNC:13112	14	14q23-q24	zinc finger ar	protein-codir	ZBTB25	zinc finger ar	O	zinc finger ar	20130804
1	VLDLR	VLDLR	1.66366	5.42939	5.29505	3.76573	7436	RP11-320E16	CARMQ1|CH	HGNC:12698	9	9p24	very low den	protein-codir	VLDLR	very low den	O	VLDL recepto	20130804
1	VEGFA	VEGFA	2.27897	6.86428	6.36811	4.58531	7422	RP1-261G23.	MVCD1|VEG	HGNC:12680	6	6p12	vascular end	protein-codir	VEGFA	vascular end	O	vascular perr	20130806
2	VARS	VARS	1.01946	0.259067	0.846049	1.27853	7407	DAAP-21F2.2	G7A|VARS1|	HGNC:12651	6	6p21.3	valyl-tRNA sy	protein-codir	VARS	valyl-tRNA sy	O	protein G7a|	20130804
3	UBA7	UBA7	5.38076	-4.83856	0.443249	-4.93751	7318	-	D8|UBA1B|U	HGNC:12471	3	3p21	ubiquitin-like	protein-codir	UBA7	ubiquitin-like	O	UBA1, ubiqui	20130804
2	CCR2	CCR2	1.13345	-0.0579517	0.402467	1.0755	729230	hCG_14621	CC-CKR-2|CC	HGNC:1603|	3	3p21.31	chemokine ((	protein-codir	CCR2	chemokine ((	O	C-C chemokir	20130803
4	TUBB2A	TUBB2A	1.55608	0.580469	-0.693481	0.862597	7280	-	TUBB|TUBB2	HGNC:12412	6	6p25	tubulin, beta	protein-codir	TUBB2A	tubulin, beta	O	class IIa beta	20130804
1	RNF208	RNF208	1.27733	3.11375	3.20646	1.92913	727800	-	-	HGNC:25420	9	9q34.3	ring finger pr	protein-codir	RNF208	ring finger pr	O	RING finger p	20130804
2	TSTA3	TSTA3	1.10169	0.0855404	0.555263	1.18723	7264	-	FX|P35B|SDI	HGNC:12390	8	8q24.3	tissue specifi	protein-codir	TSTA3	tissue specifi	O	3-5 epimeras	20130804
2	TST	TST	1.07455	0.421422	0.678049	1.49597	7263	LL22NC01-14	RDS	HGNC:12388	22	22q13.1	thiosulfate su	protein-codir	TST	thiosulfate su	O	thiosulfate su	20130804
4	C4BPB	C4BPB	1.09862	1.00416	0.282945	1.38157	725	RP11-164O2	C4BP	HGNC:1328|	1	1q32	complement	protein-codir	C4BPB	complement	O	C4b-binding	20130807
1	TNS1	TNS1	1.00445	6.56362	6.52358	5.55917	7145	MSTP091	MST091|MST	HGNC:11973	2	2q35-q36	tensin 1	protein-codir	TNS1	tensin 1	O	Matrix-remo	20130804
1	TNNT3	TNNT3	2.45611	1.87569	1.25157	-0.580424	7140	-	TNTF	HGNC:11950	11	11p15.5	troponin T ty	protein-codir	TNNT3	troponin T ty	O	beta TnTF|be	20130804
1	TNFAIP3	TNFAIP3	1.00881	-0.0380759	-0.3005	-1.04689	7128	RP11-356I2.3	A20|OTUD7(	HGNC:11896	6	6q23	tumor necros	protein-codir	TNFAIP3	tumor necros	O	OTU domain-	20130804
1	TIMP2	TIMP2	1.13209	0.220458	0.00663104	-0.911627	7077	-	CSC-21K|DD(	HGNC:11821	17	17q25	TIMP metallc	protein-codir	TIMP2	TIMP metallc	O	TIMP-2|meta	20130804
1	KLF10	KLF10	1.09842	1.57337	1.37399	0.474954	7071	-	EGR-alpha|E	HGNC:11810	8	8q22.2	Kruppel-like f	protein-codir	KLF10	Kruppel-like f	O	Krueppel-like	20130804
1	TEC	TEC	1.0169	1.7648	1.89939	0.882492	100124696	-	-	MIM:227050	19	19q13.2	transient ery	unknown	-	-	-	-	20130220
1	TCEA3	TCEA3	1.52709	3.16507	3.08763	1.63798	6920	-	TFIIS|TFIIS.H	HGNC:11615	1	1p36.12	transcription	protein-codir	TCEA3	transcription	O	rhabdomyos	20130804
1	TBX6	TBX6	1.69456	1.74408	1.53236	0.0495216	6911	-	-	HGNC:11605	16	16p11.2	T-box 6	protein-codir	TBX6	T-box 6	O	T-box proteir	20130804
1	ZFP36L2	ZFP36L2	1.10328	0.0663862	-0.168968	-1.03689	678	-	BRF2|ERF-2|	HGNC:1108|	2	2p22.3-p21	ZFP36 ring fir	protein-codir	ZFP36L2	ZFP36 ring fir	O	EGF-respons	20130804
1	SCARNA2	SCARNA2	1.62033	-0.0194224	-0.441162	-1.63975	677766	-	HBII-382|mg	HGNC:32558	1	1q13.1	small Cajal b	miscRNA	SCARNA2	small Cajal b	O	-	20130416
1	SSTR3	SSTR3	2.40247	0.334984	-0.905689	-2.06749	6753	-	SS-3-R|SS3-R	HGNC:11332	22	22q13.1	somatostatin	protein-codir	SSTR3	somatostatin	O	somatostatin	20130804
2	SRM	SRM	1.1748	-0.540998	-0.0962325	0.633802	6723	-	PAPT|SPDSY	HGNC:11296	1	1p36-p22	spermidine s	protein-codir	SRM	spermidine s	O	putrescine ar	20130804
1	SPINT1	SPINT1	1.18306	1.80654	1.7807	0.623479	6692	UNQ223/PR(	HAI|HAI1|M	HGNC:11246	15	15q15.1	serine peptid	protein-codir	SPINT1	serine peptid	O	HAI-1|hepat	20130804
1	SPINK2	SPINK2	2.05812	-0.204672	-1.81191	-2.26279	6691	-	HUSI-II	HGNC:11245	4	4q12	serine peptid	protein-codir	SPINK2	serine peptid	O	epididymis ti	20130709
1	BNIP3L	BNIP3L	1.38523	2.81668	2.5876	1.43145	665	-	BNIP3a|NIX	HGNC:1085|	8	8p21	BCL2/adenov	protein-codir	BNIP3L	BCL2/adenov	O	BCL2/adenov	20130804
1	BNIP3	BNIP3	1.77517	3.33502	3.04592	1.55985	664	-	NIP3	HGNC:1084|	10	10q26.3	BCL2/adenov	protein-codir	BNIP3	BCL2/adenov	O	BCL2/adenov	20130806
1	SLC2A11	SLC2A11	1.12628	-0.260105	-0.38889	-1.38639	66035	-	GLUT10|GLU	HGNC:14239	22	22q11.23	solute carrier	protein-codir	SLC2A11	solute carrier	O	GLUT-10|GLU	20130623
2	FAM173A	FAM173A	2.17033	-2.45785	-0.929646	-0.287524	65990	LA16c-444G9	C16orf24	HGNC:14152	16	16p13.3	family with s	protein-codir	FAM173A	family with s	O	protein FAM	20130804
4	SLC18A2	SLC18A2	1.08386	-0.713899	-1.47154	-0.387685	6571	-	SVAT|SVMT|	HGNC:10935	10	10q25	solute carrier	protein-codir	SLC18A2	solute carrier	O	monoamine	20130804
1	KIAA0895L	KIAA0895L	1.03934	3.40212	3.30624	2.36278	653319	-	-	HGNC:34408	16	16q22.1	KIAA0895-lik	protein-codir	KIAA0895L	KIAA0895-lik	O	uncharacteri	20130216
2	PYCRL	PYCRL	1.21479	0.778745	1.15289	1.99353	65263	-	-	HGNC:25846	8	8q24.3	pyrroline-5-c	protein-codir	PYCRL	pyrroline-5-c	O	P5C reductas	20130804
1	SLC2A3	SLC2A3	1.19338	2.29602	2.019	1.10264	6515	-	GLUT3	HGNC:11007	12	12p13.3	solute carrier	protein-codir	SLC2A3	solute carrier	O	GLUT-3|gluc	20130804

2	NOL6	NOL6	1.19055	-1.11743	-0.564978	0.0731177	65083	-	NRAP|UTP22	HGNC:19910	9	9p13.3	nucleolar prc	protein-codir	NOL6	nucleolar prc	O	nucleolar RN	20130804
1	EPS8L2	EPS8L2	1.37039	2.12182	1.62408	0.751429	64787	PP13181	EPS8R2	HGNC:21296	11	11p15.5	EPS8-like 2	protein-codir	EPS8L2	EPS8-like 2	O	EPS8-like pro	20130804
2	LOC644656	LOC644656	1.06881	-1.61259	-0.881953	-0.543781	644656	-	-	-	11	11p15.4	uncharacteri:	miscRNA	-	-	-	-	20130216
2	SGK1	SGK1	1.20079	0.655892	1.12268	1.85668	6446	RP1-188K17.	SGK	HGNC:10810	6	6q23	serum/gluco	protein-codir	SGK1	serum/gluco	O	Sgk1 variant	20130804
1	KANSL1-AS1	KANSL1-AS1	1.05763	0.435466	0.161547	-0.622164	644246	-	-	HGNC:43740	17	17q21.31	KANSL1 antis	miscRNA	KANSL1-AS1	KANSL1 antis	O	-	20130415
1	KANSL1-AS1	KANSL1-AS1	1.05762	0.435423	0.161547	-0.622196	644246	-	-	HGNC:43740	17	17q21.31	KANSL1 antis	miscRNA	KANSL1-AS1	KANSL1 antis	O	-	20130415
2	SUSD1	SUSD1	1.07533	0.50079	0.984644	1.57612	64420	RP11-4O1.1	-	HGNC:25413	9	9q31.3-q33.1	sushi domair	protein-codir	SUSD1	sushi domair	O	sushi domair	20130804
4	FLVCR1-AS1	FLVCR1-AS1	1.08404	0.659935	0.0144996	1.09854	642946	-	LQK1|NCRNA	HGNC:39077	1	1q32.3	FLVCR1 antis	miscRNA	FLVCR1-AS1	FLVCR1 antis	O	-	20130216
1	TMEM91	TMEM91	1.18067	0.593903	-0.0283502	-0.586766	641649	-	DSPC3|IFITM	HGNC:32393	19	19q13.2	transmembra	protein-codir	TMEM91	transmembra	O	dispanin subt	20130724
4	FN3K	FN3K	1.18133	1.05076	-0.130571	0.234197	64122	-	-	HGNC:24822	17	17q25.3	fructosamine	protein-codir	FN3K	fructosamine	O	fructosamine	20130226
1	RBKS	RBKS	1.03701	2.95937	2.56249	1.92236	64080	-	RBSK	HGNC:30325	2	2p23.3	ribokinase	protein-codir	RBKS	ribokinase	O	5230400M11	20130804
4	CCL22	CCL22	1.09787	2.73582	2.02478	3.12265	6367	A-152E5.1	ABCD-1|DC/	HGNC:10621	16	16q13	chemokine (C	protein-codir	CCL22	chemokine (C	O	C-C motif che	20130804
2	S100P	S100P	2.18881	1.54114	2.10056	3.72995	6286	-	MIG9	HGNC:10504	4	4p16	S100 calcium	protein-codir	S100P	S100 calcium	O	S100 calcium	20130804
1	RXRA	RXRA	1.79394	1.31597	1.01439	-0.477975	6256	-	NR2B1	HGNC:10477	9	9q34.3	retinoid X rec	protein-codir	RXRA	retinoid X rec	O	nuclear recep	20130804
2	MRPL12	MRPL12	1.01258	1.21107	1.45144	2.22365	6182	-	5c5-2|L12mt	HGNC:10378	17	17q25	mitochondria	protein-codir	MRPL12	mitochondria	O	39S ribosoma	20130804
2	RORC	RORC	1.51472	-2.14088	-1.77526	-0.626165	6097	RP11-98D18.	NR1F3|RORC	HGNC:10260	1	1q21	RAR-related	protein-codir	RORC	RAR-related	O	RAR-related	20130804
2	RGS16	RGS16	1.08817	1.59534	1.44916	2.53733	6004	-	A28-RGS14|	HGNC:9997|	1	1q25-q31	regulator of	protein-codir	RGS16	regulator of	O	hRGS-r|regul	20130804
1	RBMS2	RBMS2	1.18382	0.335704	0.138272	-0.848114	5939	-	SCR3	HGNC:9909|	12	12q13.3	RNA binding	protein-codir	RBMS2	RNA binding	O	RNA-binding	20130804
1	RRAGD	RRAGD	2.16812	2.74473	2.09516	0.576607	58528	-	RAGD|bA11	HGNC:19903	6	6q15-q16	Ras-related C	protein-codir	RRAGD	Ras-related C	O	Rag D protein	20130804
2	FAM108C1	ABHD17C	1.58657	-2.12265	-1.01554	-0.53608	58489	-	FAM108C1	HGNC:26925	15	15q25.1	abhydrolase	protein-codir	ABHD17C	abhydrolase	O	abhydrolase	20130804
2	PYCR1	PYCR1	1.18497	0.376659	0.477346	1.56163	5831	-	ARCL2B|ARC	HGNC:9721|	17	17q25.3	pyrroline-5-c	protein-codir	PYCR1	pyrroline-5-c	O	P5C reductas	20130804
1	CXCL16	CXCL16	1.33914	1.04343	0.331802	-0.295708	58191	UNQ2759/PF	CXCLG16|SR	HGNC:16642	17	17p13	chemokine (C	protein-codir	CXCL16	chemokine (C	O	C-X-C motif c	20130804
1	SEMA4G	SEMA4G	1.23888	0.397437	0.17038	-0.84144	57715	RP11-108L7.	-	HGNC:10735	10	10q24.31	sema domair	protein-codir	SEMA4G	sema domair	O	semaphorin-	20130804
1	KIAA1609	TLDC1	1.22798	2.78317	2.58745	1.55519	57707	-	KIAA1609	HGNC:29325	16	16q24.1	TBC/LysM-as	protein-codir	TLDC1	TBC/LysM-as	O	TBC/LysM-as	20130804
1	ZDBF2	ZDBF2	1.215	0.540098	0.185535	-0.674907	57683	-	-	HGNC:29313	2	2q33.3	zinc finger, D	protein-codir	ZDBF2	zinc finger, D	O	DBF4-type zi	20130804
1	ZSWIM5	ZSWIM5	1.46812	-0.432909	-0.488358	-1.90103	57643	RP11-69J16.	-	HGNC:29299	1	1p34.1	zinc finger, S	protein-codir	ZSWIM5	zinc finger, S	O	zinc finger SV	20130226
1	KIAA1467	KIAA1467	1.43421	2.11063	1.70143	0.676421	57613	-	-	HGNC:29288	12	12p13.1	KIAA1467	protein-codir	KIAA1467	KIAA1467	O	uncharacteri:	20130804
1	PDZD4	PDZD4	1.20148	0.793884	0.688486	-0.407592	57595	-	LU1|PDZK4|	HGNC:21167	X	Xq28	PDZ domain	protein-codir	PDZD4	PDZ domain	O	PDZ domain-	20130804
1	CARNS1	CARNS1	1.11217	0.820126	0.762403	-0.292039	57571	-	ATPGD1	HGNC:29268	11	11q13.2	carnosine syr	protein-codir	CARNS1	carnosine syr	O	ATP-grasp dc	20130804
1	ARRDC3	ARRDC3	1.00388	-1.04722	-1.27501	-2.0511	57561	-	TLIMP	HGNC:29263	5	5q14.3	arrestin dom	protein-codir	ARRDC3	arrestin dom	O	TBP-2-like in	20130804
1	NLGN2	NLGN2	1.14915	0.694863	0.430146	-0.454285	57555	-	-	HGNC:14290	17	17p13.1	neuroligin 2	protein-codir	NLGN2	neuroligin 2	O	neuroligin-2	20130804
2	DANCR	DANCR	1.03772	-1.22422	-0.665841	-0.186498	57291	-	AGU2|ANCR	HGNC:28964	4	4q12	differentiatio	miscRNA	DANCR	differentiatio	O	-	20130705
2	ADCK1	ADCK1	1.26316	-1.31005	-0.766242	-0.046886	57143	-	-	HGNC:19038	14	14q24.3	aarF domain	protein-codir	ADCK1	aarF domain	O	uncharacteri:	20130804
2	C10orf2	C10orf2	1.12254	-1.66375	-1.18905	-0.541212	56652	RP11-108L7.	ATXN8|IOSC	HGNC:1160|	10	10q24	chromosome	protein-codir	C10orf2	chromosome	O	T7 gp4-like p	20130804
2	PSEN2	PSEN2	1.11988	-1.7004	-0.995617	-0.580517	5664	-	AD3L|AD4|C	HGNC:9509|	1	1q31-q42	presenilin 2 (	protein-codir	PSEN2	presenilin 2 (	O	AD3LP|AD5|	20130804
1	PSD	PSD	1.25771	0.233735	0.131061	-1.02398	5662	RP11-18I14.2	EFA6|EFA6A	HGNC:9507|	10	10q24	pleckstrin an	protein-codir	PSD	pleckstrin an	O	PH and SEC7	20130804
1	CCL28	CCL28	1.51525	1.08986	1.18355	-0.331703	56477	-	CCK1|MEC|S	HGNC:17700	5	5p12	chemokine (C	protein-codir	CCL28	chemokine (C	O	C-C motif che	20130803
1	MESP1	MESP1	1.31846	1.82315	1.9862	0.667741	55897	-	bHLHc5	HGNC:29658	15	15q26.1	mesoderm p	protein-codir	MESP1	mesoderm p	O	class C basic	20130226
1	ZNF395	ZNF395	1.91268	2.19992	1.79248	0.287236	55893	-	HDBP-2|HDE	HGNC:18737	8	8p21.1	zinc finger pr	protein-codir	ZNF395	zinc finger pr	O	HD gene regu	20130804
1	KDM3A	KDM3A	1.14791	1.24452	0.968031	0.0966061	55818	-	JHDM2A|JHI	HGNC:20815	2	2p11.2	lysine (K)-spe	protein-codir	KDM3A	lysine (K)-spe	O	jmjC domain-	20130804
2	IL26	IL26	1.23385	0.41328	0.234198	1.46805	55801	-	AK155|IL-26	HGNC:17119	12	12q15	interleukin 2	protein-codir	IL26	interleukin 2	O	interleukin-2	20130804
1	RAB20	RAB20	1.9035	4.25892	4.19845	2.35542	55647	-	-	HGNC:18260	13	13q34	RAB20, mem	protein-codir	RAB20	RAB20, mem	O	ras-related p	20130804
2	LYAR	LYAR	1.05455	-1.39368	-1.0478	-0.339134	55646	PNAS-5	ZC2HC2|ZLY	HGNC:26021	4	4p16.3	Ly1 antibody	protein-codir	LYAR	Ly1 antibody	O	Ly1 antibody	20130804
1	CDC42BPG	CDC42BPG	1.08274	0.641852	0.172128	-0.440889	55561	-	DMPK2|HSM	HGNC:29829	11	11q13.1	CDC42 bindir	protein-codir	CDC42BPG	CDC42 bindir	O	CDC42-bindi	20130804
2	BATF3	BATF3	1.43141	1.04652	1.7851	2.47793	55509	-	JDP1|JUNDN	HGNC:28915	1	1q32.3	basic leucine	protein-codir	BATF3	basic leucine	O	21 kDa small	20130804
2	CHDH	CHDH	1.10904	0.71267	1.09809	1.82171	55349	-	-	HGNC:24288	3	3p21.1	choline dehy	protein-codir	CHDH	choline dehy	O	CDH|CHD|ch	20130804
1	PPP2R5B	PPP2R5B	1.02515	1.11647	0.87699	0.0913248	5526	-	B56B|PR61B	HGNC:9310|	11	11q12	protein phos	protein-codir	PPP2R5B	protein phos	O	PP2A B subu	20130804
2	FAM86C1	FAM86C1	1.65294	-1.48456	-0.947977	0.168379	55199	-	FAM86C	HGNC:25561	11	11q13.4	family with s	protein-codir	FAM86C1	family with s	O	family with s	20130804
2	FAIM	FAIM	1.3256	-1.80468	-1.17869	-0.479076	55179	-	FAIM1	HGNC:18703	3	3q22.3	Fas apoptotic	protein-codir	FAIM	Fas apoptotic	O	fas apoptotic	20130804
1	C19orf73	C19orf73	2.01091	-0.946663	0.0132143	-1.9977	55150	-	-	HGNC:25534	19	19q13.33	chromosome	protein-codir	C19orf73	chromosome	O	putative uncl	20130226
1	ANKZF1	ANKZF1	1.18081	1.29042	1.29537	0.114564	55139	-	ZNF744	HGNC:25527	2	2q35	ankyrin repea	protein-codir	ANKZF1	ankyrin repea	O	ankyrin repea	20130804
1	EXD3	EXD3	1.24246	0.502922	0.222399	-0.739534	54932	HBE269	mut-7	HGNC:26023	9	9q34.3	exonuclease	protein-codir	EXD3	exonuclease	O	exonuclease	20130804
2	GPATCH4	GPATCH4	1.55693	-0.663296	0.12127	0.893633	54865	RP11-284F21	GPATC4	HGNC:25982	1	1q22	G patch dom	protein-codir	GPATCH4	G patch dom	O	G patch dom	20130804
1	FAM46C	FAM46C	1.18055	-0.133086	-0.390609	-1.31364	54855	-	-	HGNC:24712	1	1p12	family with s	protein-codir	FAM46C	family with s	O	protein FAM	20130804
1	KLHL24	KLHL24	1.01907	-1.51666	-1.82291	-2.53573	54800	-	DRE1|KRIP6	HGNC:25947	3	3q27.1	kelch-like fan	protein-codir	KLHL24	kelch-like fan	O	kainate recep	20130804
1	PPARD	PPARD	1.14705	1.26096	1.20517	0.113908	5467	-	FAAR|NR1C2	HGNC:9235|	6	6p21.2	peroxisome p	protein-codir	PPARD	peroxisome p	O	PPAR-beta|P	20130804
1	EGLN1	EGLN1	1.21395	1.89075	1.68298	0.676803	54583	PNAS-118	C1orf12|ECY	HGNC:1232|	1	1q42.1	egl nine hom	protein-codir	EGLN1	egl nine hom	O	HIF prolyl hy	20130804
1	SDK2	SDK2	2.58522	5.57505	5.04646	2.98983	54549	-	-	HGNC:19308	17	17q25.1	sidekick cell	protein-codir	SDK2	sidekick cell	O	protein sidek	20130804
1	DDIT4	DDIT4	1.57606	4.71593	4.4628	3.13987	54541	RP11-442H2	Dig2|REDD-1	HGNC:24944	10	10q22.1	DNA-damage	protein-codir	DDIT4	DNA-damage	O	DNA damage	20130804
4	ANKRD16	ANKRD16	1.64463	0.360534	-1.2841	0.00646966	54522	RP11-318E3.	-	HGNC:23471	10	10p15.1	ankyrin repea	protein-codir	ANKRD16	ankyrin repea	O	ankyrin repea	20130708
2	EXOSC4	EXOSC4	1.00448	0.77358	0.372099	1.37658	54512	-	RRP41|RRP4	HGNC:18189	8	8q24.3	exosome con	protein-codir	EXOSC4	exosome con	O	exosome con	20130804
1	C11orf71	C11orf71	1.45326	0.418754	-0.310999	-1.03451	54494	-	-	HGNC:25937	11	11q23.2	chromosome	protein-codir	C11orf71	chromosome	O	uncharacteri:	20130804
2	FAM64A	FAM64A	1.04224	4.90877	5.19167	5.95101	54478	-	CATS|RCS1	HGNC:25483	17	17p13.2	family with s	protein-codir	FAM64A	family with s	O	CALM interac	20130804
1	TAS2R5	TAS2R5	1.90368	-0.50882	0.420411	-1.48327	54429	-	T2R5	HGNC:14912	7	7q31.3-q32	taste recepto	protein-codir	TAS2R5	taste recepto	O	taste recepto	20130627
2	POLR2L	POLR2L	1.33463	-0.0872673	0.502996	1.24736	5441	-	RBP10|RPAB	HGNC:9199|	11	11p15	polymerase (	protein-codir	POLR2L	polymerase (	O	DNA-directec	20130804
2	GDAP1	GDAP1	1.09213	-0.740237	-0.144882	0.351894	54332	-	CMT4|CMT4	HGNC:15968	8	8q21.11	ganglioside ir	protein-codir	GDAP1	ganglioside ir	O	Charcot-Mar	20130804
1	C21orf49	C21orf49	1.05841	-1.45875	-1.55481	-2.51716	54067	-	-	HGNC:1290|	21	21q22.11	chromosome	miscRNA	C21orf49	chromosome	O	-	20130226
1	PMS2P3	PMS2P3	1.19131	-0.241405	0.342654	-0.848653	5387	-	PMS2L3|PM	HGNC:9128|	7	7q11.23	postmeiotic s	pseudo	PMS2P3	postmeiotic s	O	-	20130226
1	ATP7A	ATP7A	1.51008	1.20153	0.763188	-0.308553	538	RP3-465G10.	DSMAX|MK|	HGNC:869|M	X	Xq21.1	ATPase, Cu++	protein-codir	ATP7A	ATPase, Cu++	O	Cu++-transpo	20130804
1	S1PR5	S1PR5	1.45226	-0.571414	-0.700693	-2.02367	53637	-	EDG8|Edg-8	HGNC:14299	19	19p13.2	sphingosine-	protein-codir	S1PR5	sphingosine-	O	S1P receptor	20130804
2	PLK1	PLK1	1.28457	1.51042	2.21269	2.79499	5347	-	PLK|STPK13	HGNC:9077|	16	16p12.2	polo-like kin	protein-codir	PLK1	polo-like kin	O	PLK-1|cell cy	20130804
2	SPA17	SPA17	1.07211	-0.203097	-0.0694373	0.869008	53340	-	CT22|SP17|	HGNC:11210	11	11q24.2	sperm autoa	protein-codir	SPA17	sperm autoa	O	cancer/testis	20130804
1	PLAG1	PLAG1	1.22736	0.632956	0.21975	-0.594406	5324	-	PSA|SGPA|Z	HGNC:9045|	8	8q12	pleiomorphic	protein-codir	PLAG1	pleiomorphic	O	COL1A2/PLA	20130804
1	SERPINE2	SERPINE2	1.317	3.34518	3.09024	2.02818	5270	-	GDN|GDNPF	HGNC:8951|	2	2q36.1	serpin peptid	protein-codir	SERPINE2	serpin peptid	O	glia-derived	20130804
1	PGK1	PGK1	1.11932	3.87024	3.62406	2.75092	5230	RP4-570L12.	MIG10|PGKA	HGNC:8896|	X	Xq13.3	phosphoglyc	protein-codir	PGK1	phosphoglyc	O	PRP 2|cell m	20130804
1	PFKFB4	PFKFB4	2.19689	6.91961	6.61096	4.72272	5210	-	-	HGNC:8875|	3	3p22-p21	6-phosphofru	protein-codir	PFKFB4	6-phosphofru	O	6-phosphofru	20130804
1	PFKFB3	PFKFB3	1.11123	1.03593	0.746193	-0.0753027	5209	RP11-298K2	IPFK2|PFK2	HGNC:8874|	10	10p15.1	6-phosphofru	protein-codir	PFKFB3	6-phosphofru	O	6-phosphofru	20130804
1	PER1	PER1	1.03797	0.603945	0.31919	-0.434026	5187	-	PER|RIGUI|	HGNC:8845|	17	17p13.1	period circad	protein-codir	PER1	period circad	O	Period, drosc	20130804
1	TMEM8B	TMEM8B	1.06117	0.263367	0.159655	-0.797802	51754	RP11-112J3.	C9orf127|NA	HGNC:21427	9	9p13.3	transmembra	protein-codir	TMEM8B	transmembra	O	Protein NGX	20130804
2	DNAJB11	DNAJB11	1.11928	-0.189709	0.222657	0.929568	51726	PSEC0121	ABBP-2|ABB	HGNC:14889	3	3q27.3	DnaJ (Hsp40)	protein-codir	DNAJB11	DnaJ (Hsp40)	O	APOBEC1-bir	20130804
2	PDK1	PDK1	1.21787	1.72406	1.42955	0.50619	5163	-	-	HGNC:8809|	2	2q31.1	pyruvate deh	protein-codir	PDK1	pyruvate deh	O	PDH kinase 1	20130804
1	NCKIPSD	NCKIPSD	1.08611	3.19426	3.05608	2.10815	51517	-	AF3P21|DIP	HGNC:15486	3	3p21	NCK interacti	protein-codir	NCKIPSD	NCK interacti	O	54 kDa VacA	20130804
2	NOP16	NOP16	1.37135	-0.408474	0.153425	0.962874	51491	CGI-117	HSPC111|HS	HGNC:26934	5	5q35.2	NOP16 nucle	protein-codir	NOP16	NOP16 nucle	O	HBV pre-S2 t	20130804
1	PDE4D	PDE4D	1.03355	1.63037	1.25963	0.596821	5144	-	ACRDYS2|DP	HGNC:8783|	5	5q12	phosphodies	protein-codir	PDE4D	phosphodies	O	cAMP-specifi	20130804
2	ZNF771	ZNF771	1.18483	-1.63341	-0.733903	-0.44858	51333	-	DSC43	HGNC:29653	16	16p11.2	zinc finger pr	protein-codir	ZNF771	zinc finger pr	O	mesenchyma	20130709
1	PLAC8	PLAC8	1.24919	2.22924	1.90994	0.980049	51316	BM-004	C15|onzin	HGNC:19254	4	4q21.22	placenta-spe	protein-codir	PLAC8	placenta-spe	O	placenta-spe	20130804
1	SLC22A17	SLC22A17	1.32945	0.0438045	-0.112945	-1.28565	51310	-	24p3R|BOCT	HGNC:23095	14	14q11.2	solute carrier	protein-codir	SLC22A17	solute carrier	O	24p3 recepto	20130804
1	NRN1	NRN1	1.80202	5.89807	5.88772	4.09605	51299	-	NRN|dJ380B	HGNC:17972	6	6p25.1	neuritin 1	protein-codir	NRN1	neuritin 1	O	neuritin	20130723
2	MRPL27	MRPL27	1.10625	0.14814	0.502561	1.25439	51264	HSPC250	L27mt	HGNC:14483	17	17q21.3-q22	mitochondria	protein-codir	MRPL27	mitochondria	O	39S ribosoma	20130804
1	PCSK5	PCSK5	1.36027	1.10273	0.437413	-0.257535	5125	RP11-422N1	PC5|PC6|PC	HGNC:8747|	9	9q21.3	proprotein co	protein-codir	PCSK5	proprotein co	O	hPC6|prohor	20130804
2	MRTO4	MRTO4	1.14345	0.514977	0.889781	1.65843	51154	-	C1orf33|MR	HGNC:18477	1	1p36.13	mRNA turnov	protein-codir	MRTO4	mRNA turnov	O	60S acidic rib	20130804
1	INSIG2	INSIG2	1.27827	2.11528	1.98696	0.837014	51141	-	-	HGNC:20452	2	2q14.2	insulin induc	protein-codir	INSIG2	insulin induc	O	INSIG-2|INSI	20130804
2	MECR	MECR	1.03688	-0.144701	-0.0778729	0.89218	51102	RP11-467D1	CGI-63|FASN	HGNC:19691	1	1p35.3	mitochondria	protein-codir	MECR	mitochondria	O	NRBF-1|hom	20130804
1	PAM	PAM	1.01607	0.509291	0.365203	-0.506777	5066	-	PAL|PHM	HGNC:8596|	5	5q14-q21	peptidylglyci	protein-codir	PAM	peptidylglyci	O	pancreatic pe	20130804
1	P4HA1	P4HA1	1.54304	3.86122	3.38973	2.31818	5033	RP11-344N1	P4HA	HGNC:8546|	10	10q21.3-q23	prolyl 4-hydr	protein-codir	P4HA1	prolyl 4-hydr	O	C-P4Halpha(	20130804
2	CHAC2	CHAC2	1.17215	0.857443	1.49563	2.02959	494143	-	-	HGNC:32363	2	2p16	ChaC, cation	protein-codir	CHAC2	ChaC, cation	O	ChaC, cation	20130804
1	NTRK1	NTRK1	2.01512	5.48916	5.05912	3.47404	4914	-	MTC|TRK|TF	HGNC:8031|	1	1q21-q22	neurotrophic	protein-codir	NTRK1	neurotrophic	O	Oncogene TR	20130804
1	NPAS2	NPAS2	1.50269	0.946415	0.637627	-0.556276	4862	-	MOP4|PASD	HGNC:7895|	2	2q11.2	neuronal PAS	protein-codir	NPAS2	neuronal PAS	O	PAS domain-	20130804
2	NEK3	NEK3	1.2951	-1.38441	-0.740524	-0.0893066	4752	RP11-248G5.	HSPK36	HGNC:7746|	13	13q14.13	NIMA-relatec	protein-codir	NEK3	NIMA-relatec	O	HSPK 36|NIN	20130804
2	NDUFS6	NDUFS6	1.08412	0.0626459	0.496652	1.14677	4726	-	CI-13kA|CI-1	HGNC:7713|	5	5p15.33	NADH dehyd	protein-codir	NDUFS6	NADH dehyd	O	NADH dehyd	20130804
1	MYH10	MYH10	1.38384	2.18214	1.87809	0.798299	4628	-	NMMHC-IIB|	HGNC:7568|	17	17p13	myosin, heav	protein-codir	MYH10	myosin, heav	O	cellular myos	20130804
1	MXI1	MXI1	1.81243	1.28416	0.985338	-0.528273	4601	RP11-549L6.	MAD2|MXD2	HGNC:7534|	10	10q24-q25	MAX interact	protein-codir	MXI1	MAX interact	O	MAX dimeriz	20130804
1	MT3	MT3	2.88785	5.46164	4.72198	2.57379	4504	-	GIF|GIFB|GR	HGNC:7408|	16	16q13	metallothion	protein-codir	MT3	metallothion	O	MT-3|MT-III	20130804
1	MST1	MST1	1.09537	-0.0715376	-0.523823	-1.16691	4485	-	D3F15S2|DN	HGNC:7380|	3	3p21	macrophage	protein-codir	MST1	macrophage	O	hepatocyte g	20130804
1	MYO1B	MYO1B	1.17723	4.97785	4.92089	3.80062	4430	-	myr1	HGNC:7596|	2	2q12-q34	myosin IB	protein-codir	MYO1B	myosin IB	O	MMI-alpha|	20130416
1	FAM26F	FAM26F	1.43067	3.30055	3.25697	1.86988	441168	RP1-93H18.5	C6orf187|dJ	HGNC:33391	6	6q22.1	family with s	protein-codir	FAM26F	family with s	O	protein FAM	20130416
1	ODF3B	ODF3B	1.52594	-0.423558	-0.153607	-1.67955	440836	-	ODF3L3	HGNC:34388	22	22q13.33	outer dense	protein-codir	ODF3B	outer dense	O	orf2 5' to PD	20130613
1	SLC2A1-AS1	SLC2A1-AS1	1.32218	2.30001	2.92093	1.59875	440584	-	-	HGNC:44187	1	1p34.2	SLC2A1 antis	miscRNA	SLC2A1-AS1	SLC2A1 antis	O	-	20130226
1	DNM1P41	DNM1P41	1.54091	1.0946	1.28719	-0.253724	440299	-	DNM1DN11-	HGNC:35191	15	15q25.2	DNM1 pseud	pseudo	DNM1P41	DNM1 pseud	O	-	20130717
1	THAP7-AS1	THAP7-AS1	1.72205	0.440083	0.0258442	-1.28197	439931	-	-	HGNC:41013	22	22q11.21	THAP7 antise	miscRNA	THAP7-AS1	THAP7 antise	O	-	20130226
1	MMP9	MMP9	1.15732	-0.93929	-1.02767	-2.09661	4318	-	CLG4B|GELB	HGNC:7176|	20	20q11.2-q13	matrix metal	protein-codir	MMP9	matrix metal	O	92 kDa gelati	20130804
2	METTL1	METTL1	1.30404	0.329181	1.0614	1.63322	4234	-	C12orf1|TRN	HGNC:7030|	12	12q13	methyltransf	protein-codir	METTL1	methyltransf	O	D1075-like g	20130804
3	MC1R	MC1R	1.13265	-0.839214	0.29344	0.0409809	4157	-	CMM5|MSH	HGNC:6929|	16	16q24.3	melanocortir	protein-codir	MC1R	melanocortir	O	MC1-R|mela	20130804
2	MARS	MARS	1.0445	-0.963831	-0.626702	0.0806713	4141	-	METRS|MRS	HGNC:6898|	12	12q13	methionyl-tR	protein-codir	MARS	methionyl-tR	O	cytosolic met	20130804
2	ARSB	ARSB	1.10701	-0.14005	0.132907	0.966963	411	-	ASB|G4S|MI	HGNC:714|M	5	5q14.1	arylsulfatase	protein-codir	ARSB	arylsulfatase	O	N-acetylgalac	20130804
4	LYZ	LYZ	1.61683	-3.33981	-4.95664	-3.87686	4069	-	LZM	HGNC:6740|	12	12q15	lysozyme	protein-codir	LYZ	lysozyme	O	1,4-beta-N-a	20130804
1	LY9	LY9	1.24903	-1.90123	-2.66222	-3.15026	4063	RP11-312J18	CD229|SLAN	HGNC:6730|	1	1q23.3	lymphocyte a	protein-codir	LY9	lymphocyte a	O	SLAM family	20130804
2	LTK	LTK	1.51007	-2.45395	-1.3209	-0.943882	4058	-	TYK1	HGNC:6721|	15	15q15.1-q21	leukocyte rec	protein-codir	LTK	leukocyte rec	O	leukocyte tyr	20130804
2	UFSP1	UFSP1	1.31278	-1.79088	-1.28997	-0.478096	402682	tcag7.1226	UFSP	HGNC:33821	7	7q22.1	UFM1-specif	protein-codir	UFSP1	UFM1-specif	O	inactive Ufm	20130613

4	FAM224A	FAM224A	2.79049	-0.589244	-2.96751	-0.177016	401630	-	LINC00230A|	HGNC:37736	Y	Yq11.222	family with s	miscRNA	FAM224A	family with s	O	-	20130226
4	FAM224A	FAM224A	2.76068	-0.587995	-2.93476	-0.174083	401630	-	LINC00230A|	HGNC:37736	Y	Yq11.222	family with s	miscRNA	FAM224A	family with s	O	-	20130226
1	LOXL1	LOXL1	1.19478	4.55994	4.17419	3.36516	4016	-	LOL|LOXL	HGNC:6665|	15	15q22	lysyl oxidase-	protein-codir	LOXL1	lysyl oxidase-	O	lysyl oxidase	20130804
1	C4orf3	C4orf3	1.18271	1.36483	1.10565	0.182124	401152	-	-	HGNC:19225	4	4q26	chromosome	protein-codir	C4orf3	chromosome	O	HCV F-transa	20130430
2	C4orf48	C4orf48	1.3946	-0.205318	-0.433878	0.960724	401115	Chr4_55	CHR4_55	HGNC:34437	4	4p16.3	chromosome	protein-codir	C4orf48	chromosome	O	neuropeptid	20130226
3	FLJ42351	FLJ42351	1.22615	0.0973535	1.3235	0.799116	400999	-	-	-	2	2q13	uncharacteri:	miscRNA	-	-	-	-	20130226
1	PAIP2B	PAIP2B	1.42019	0.0513548	-0.104467	-1.36884	400961	-	-	HGNC:29200	2	2p13.3	poly(A) bindi	protein-codir	PAIP2B	poly(A) bindi	O	PABP-interac	20130804
1	LOC400927	LOC400927	1.18735	0.406727	-0.0135835	-0.780622	400927	-	-	-	22	22q13.1	TPTE and PTE	pseudo	-	-	-	hypothetical	20130226
1	LMO7	LMO7	1.14739	-0.236344	-0.496104	-1.38373	4008	RP11-332E3.	FBX20|FBXO	HGNC:6646|	13	13q22.2	LIM domain	protein-codir	LMO7	LIM domain	O	F-box only pr	20130804
3	GRAPL	GRAPL	2.11909	-0.447304	1.11486	-1.00423	400581	-	-	HGNC:37240	17	17p11.2	GRB2-relatec	protein-codir	GRAPL	GRB2-relatec	O	GRB2-relatec	20130226
1	DICER1-AS1	DICER1-AS1	1.0739	-0.944407	-0.434438	-1.50834	400242	-	DICER1-AS	HGNC:43017	14	14q32.13	DICER1 antis	miscRNA	DICER1-AS1	DICER1 antis	O	-	20130804
2	SLC25A35	SLC25A35	1.14403	-1.08506	-1.52246	-0.378434	399512	-	-	HGNC:31921	17	17p13.1	solute carrie	protein-codir	SLC25A35	solute carrie	O	solute carrie	20130703
1	L1CAM	L1CAM	1.44671	2.41144	1.89708	0.964734	3897	-	CAML1|CD1	HGNC:6470|	X	Xq28	L1 cell adhes	protein-codir	L1CAM	L1 cell adhes	O	antigen ident	20130804
2	LOC389641	LOC389641	1.00012	-1.29694	-0.910121	-0.296816	389641	-	-	Ensembl:ENS	8	8p21.3	uncharacteri:	miscRNA	-	-	-	-	20130725
4	LOC389634	LINC00937	1.133	-0.293247	-1.42625	-1.04551	389634	-	-	HGNC:48629	12	12p13.31	long intergen	miscRNA	LINC00937	long intergen	O	-	20130602
1	GTF2IRD2B	GTF2IRD2B	1.13145	-0.912035	-1.07063	-2.04348	389524	-	-	HGNC:33125	7	7q11.23	GTF2I repeat	protein-codir	GTF2IRD2B	GTF2I repeat	O	GTF2I repeat	20130804
2	FAM211B	FAM211B	1.02124	1.67459	1.36036	2.3816	388886	-	C22orf36	HGNC:33155	22	22q11.23	family with s	protein-codir	FAM211B	family with s	O	leucine-rich r	20130226
1	SBK1	SBK1	1.09968	0.398809	0.333402	-0.700872	388228	-	SBK	HGNC:17699	16	16p11.2	SH3-binding	protein-codir	SBK1	SH3-binding	O	serine/threo	20130804
2	KPNA2	KPNA2	1.25322	0.845271	1.39669	2.09849	3838	-	IPOA1|QIP2|	HGNC:6395|	17	17q24.2	karyopherin	protein-codir	KPNA2	karyopherin	O	RAG cohort 1	20130804
1	AGRN	AGRN	1.15275	1.20004	0.917685	0.0472881	375790	RP11-54O7.1	-	HGNC:329|M	1	1p36.33	agrin	protein-codir	AGRN	agrin	O	agrin proteog	20130804
1	PNPLA7	PNPLA7	1.22457	-0.36589	-0.765712	-1.59046	375775	RP11-48C7.2	C9orf111|NT	HGNC:24768	9	9q34.3	patatin-like p	protein-codir	PNPLA7	patatin-like p	O	patatin-like p	20130226
2	LRRC33	NRROS	1.1132	0.0450821	0.209078	1.15828	375387	UNQ3030/PF	GARPL1|LRR	HGNC:24613	3	3q29	negative regu	protein-codir	NRROS	negative regu	O	ELLP3030|le	20130804
1	HSD11B1L	HSD11B1L	1.00255	0.397327	0.822402	-0.180151	374875	-	11-DH3|11-b	HGNC:30419	19	19p13.3	hydroxystero	protein-codir	HSD11B1L	hydroxystero	O	11-beta-hydr	20130804
2	IL13	IL13	2.01602	3.08396	3.91769	5.09998	3596	-	IL-13|P600	HGNC:5973|	5	5q31	interleukin 1	protein-codir	IL13	interleukin 1	O	interleukin-1	20130804
1	ANKRD37	ANKRD37	1.81998	3.8287	3.54353	2.00872	353322	-	Lrp2bp	HGNC:29593	4	4q35.1	ankyrin repea	protein-codir	ANKRD37	ankyrin repea	O	ankyrin repea	20130804
1	DPY19L2P2	DPY19L2P2	1.09015	2.11935	1.90036	1.0292	349152	-	-	HGNC:21764	7	7q22.1	dpy-19-like 2	pseudo	DPY19L2P2	dpy-19-like 2	O	-	20130226
2	CTU2	CTU2	1.40466	-0.588361	0.27906	0.816295	348180	-	C16orf84|NC	HGNC:28005	16	16q24.3	cytosolic thic	protein-codir	CTU2	cytosolic thic	O	cytoplasmic t	20130804
1	IGF1R	IGF1R	1.27814	-0.208533	-0.536061	-1.48667	3480	-	CD221|IGFIR	HGNC:5465|	15	15q26.3	insulin-like g	protein-codir	IGF1R	insulin-like g	O	IGF-I recepto	20130804
2	IFNG	IFNG	1.48061	-0.192247	0.241655	1.28836	3458	-	IFG|IFI	HGNC:5438|	12	12q14	interferon, g	protein-codir	IFNG	interferon, g	O	IFN-gamma|	20130804
1	ZNF404	ZNF404	1.09556	0.0340796	-0.220848	-1.06148	342908	-	-	HGNC:19417	19	19q13.31	zinc finger pr	protein-codir	ZNF404	zinc finger pr	O	-	20130709
1	ZNF517	ZNF517	1.28904	-0.145984	-0.803014	-1.43502	340385	-	-	HGNC:27984	8	8q24.3	zinc finger pr	protein-codir	ZNF517	zinc finger pr	O	-	20130709
4	SLC35E4	SLC35E4	1.80015	0.261468	-0.997939	0.802214	339665	-	-	HGNC:17058	22	22q12.2	solute carrie	protein-codir	SLC35E4	solute carrie	O	solute carrie	20130804
2	HPGD	HPGD	1.05728	1.18506	1.62889	2.24234	3248	-	15-PGDH|PG	HGNC:5154|	4	4q34-q35	hydroxyprost	protein-codir	HPGD	hydroxyprost	O	15-hydroxyp	20130804
1	HLA-DQB1	HLA-DQB1	1.16651	1.90876	1.72836	0.742249	3119	DADB-249P1	CELIAC1|HLA	HGNC:4944|	6	6p21.3	major histoc	protein-codir	HLA-DQB1	major histoc	O	HLA class II h	20130804
1	HK2	HK2	1.29983	4.30311	4.14191	3.00328	3099	-	HKII|HXK2	HGNC:4923|	2	2p13	hexokinase 2	protein-codir	HK2	hexokinase 2	O	HK II|hexokir	20130804
1	KCNIP2	KCNIP2	1.55779	1.44133	1.72998	0.172188	30819	-	KCHIP2	HGNC:15522	10	10q24	Kv channel in	protein-codir	KCNIP2	Kv channel in	O	A-type potas	20130804
4	HIST1H1D	HIST1H1D	1.13655	2.4727	1.33615	1.50085	3007	-	H1.3|H1D|H	HGNC:4717|	6	6p21.3	histone clust	protein-codir	HIST1H1D	histone clust	O	H1 histone fa	20130804
1	EFEMP2	EFEMP2	1.71498	2.32034	1.88584	0.605355	30008	UNQ200/PRC	ARCL1B|FBLI	HGNC:3219|	11	11q13.1	EGF containi	protein-codir	EFEMP2	EGF containi	O	EGF-containi	20130804
1	PNMA3	PNMA3	1.10247	-0.438255	-0.922197	-1.54072	29944	-	MA3|MA5	HGNC:18742	X	Xq28	paraneoplast	protein-codir	PNMA3	paraneoplast	O	paraneoplast	20130804
1	HILPDA	HILPDA	2.64607	6.41102	5.91742	3.76495	29923	-	C7orf68|HIG	HGNC:28859	7	7q32.1	hypoxia indu	protein-codir	HILPDA	hypoxia indu	O	hypoxia indu	20130804
1	YPEL1	YPEL1	1.36917	0.223944	-0.152999	-1.14523	29799	FKSG3	-	HGNC:12845	22	22q11.2	yippee-like 1	protein-codir	YPEL1	yippee-like 1	O	DiGeorge syr	20130330
4	GSTT1	GSTT1	1.33344	0.436038	-0.69313	0.640308	2952	-	-	HGNC:4641|	22	22q11.23	glutathione S	protein-codir	GSTT1	glutathione S	O	GST class-the	20130804
4	GSN	GSN	1.184	-0.380478	-1.56448	-1.25269	2934	RP11-477J21	ADF|AGEL	HGNC:4620|	9	9q33	gelsolin	protein-codir	GSN	gelsolin	O	actin-depoly	20130804
1	MKNK2	MKNK2	1.5467	2.10086	1.74497	0.554159	2872	-	GPRK7|MNK	HGNC:7111|	19	19p13.3	MAP kinase i	protein-codir	MKNK2	MAP kinase i	O	G protein-co	20130804
1	ATG9B	ATG9B	1.61676	1.47126	1.20119	-0.145502	285973	-	APG9L2|NOS	HGNC:21899	7	7q36.1	autophagy re	protein-codir	ATG9B	autophagy re	O	APG9-like 2|	20130801
1	ARL10	ARL10	1.01552	0.845484	0.590292	-0.170038	285598	-	ARL10A	HGNC:22042	5	5q35.2	ADP-ribosyla	protein-codir	ARL10	ADP-ribosyla	O	ADP-ribosyla	20130804
1	FAM13A-AS1	FAM13A-AS1	1.51648	0.584373	0.406447	-0.93211	285512	-	FAM13A1OS	HGNC:19370	4	4q22.1	FAM13A anti	miscRNA	FAM13A-AS1	FAM13A anti	O	-	20130430
2	THEM5	THEM5	1.30954	-1.45398	-0.826355	-0.144439	284486	RP11-139D2	-	HGNC:26755	1	1q21.3	thioesterase	protein-codir	THEM5	thioesterase	O	acyl-CoA thic	20130226
1	LOC284023	LOC284023	1.37462	1.61152	1.47903	0.236901	284023	-	-	Ensembl:ENS	17	17p13.1	uncharacteri:	miscRNA	-	-	-	-	20130226
1	NHLRC4	NHLRC4	1.14953	0.144586	0.113679	-1.00494	283948	-	-	HGNC:26700	16	16p13.3	NHL repeat c	protein-codir	NHLRC4	NHL repeat c	O	NHL-repeat-	20130226
2	GPR15	GPR15	1.02648	-1.69884	-1.43774	-0.672362	2838	-	BOB	HGNC:4469|	3	3q11.2-q13.1	G protein-co	protein-codir	GPR15	G protein-co	O	G-protein co	20130804
1	LOC283174	MIR4697HG	1.35517	2.47145	2.3271	1.11628	283174	-	LINC00947	HGNC:27448	11	11q25	MIR4697 hos	miscRNA	MIR4697HG	MIR4697 hos	O	-	20130604
1	GPM6B	GPM6B	1.09226	-0.5378	-0.537039	-1.6293	2824	-	M6B	HGNC:4461|	X	Xp22.2	glycoprotein	protein-codir	GPM6B	glycoprotein	O	neuronal me	20130804
1	GPI	GPI	1.58299	4.19958	3.82114	2.61659	2821	-	AMF|GNPI|N	HGNC:4458|	19	19q13.1	glucose-6-ph	protein-codir	GPI	glucose-6-ph	O	autocrine mo	20130804
2	UTP20	UTP20	1.06675	-1.03815	-0.770304	0.0285953	27340	-	DRIM	HGNC:17897	12	12q23	UTP20, small	protein-codir	UTP20	UTP20, small	O	NNP73|dow	20130804
1	LYPD3	LYPD3	1.32631	0.037883	-0.88943	-1.28843	27076	UNQ491/PRC	C4.4A	HGNC:24880	19	19q13.31	LY6/PLAUR d	protein-codir	LYPD3	LY6/PLAUR d	O	2310061G07	20130804
2	ZBTB32	ZBTB32	1.6633	3.59889	4.12971	5.26219	27033	-	FAXF|FAZF|F	HGNC:16763	19	19q13.1	zinc finger ar	protein-codir	ZBTB32	zinc finger ar	O	FANCC-intera	20130804
1	GPR160	GPR160	1.45733	1.96303	1.69798	0.505704	26996	-	GPCR1|GPCF	HGNC:23693	3	3q26.2-q27	G protein-co	protein-codir	GPR160	G protein-co	O	G-protein co	20130226
1	GIPR	GIPR	1.49433	1.29968	1.0721	-0.194647	2696	-	PGQTL2	HGNC:4271|	19	19q13.3	gastric inhibi	protein-codir	GIPR	gastric inhibi	O	GIP-R|glucos	20130804
2	SNORA65	SNORA65	1.09595	0.478759	1.16468	1.57471	26783	-	RNU65|U65	HGNC:10222	9	9q34	small nucleol	snoRNA	SNORA65	small nucleol	O	-	20130226
1	FAM162A	FAM162A	1.28367	3.43369	3.31723	2.15002	26355	DC16	C3orf28|E2I	HGNC:17865	3	3q21.1	family with s	protein-codir	FAM162A	family with s	O	E2-induced g	20130804
2	GBGT1	GBGT1	1.4581	0.166692	-0.237514	1.22059	26301	RP11-326L24	A3GALNT|FS	HGNC:20460	9	9q34.13-q34	globoside alp	protein-codir	GBGT1	globoside alp	O	Forssman gly	20130720
1	WSB1	WSB1	1.16624	1.5917	1.42471	0.425462	26118	-	SWIP1|WSB-	HGNC:19221	17	17q11.1	WD repeat a	protein-codir	WSB1	WD repeat a	O	SOCS box-co	20130804
1	TANC2	TANC2	1.37235	0.107002	-0.368791	-1.26535	26115	-	ROLSA|rols	HGNC:30212	17	17q23.3	tetratricopep	protein-codir	TANC2	tetratricopep	O	protein TANC	20130226
1	GPSM1	GPSM1	1.66963	0.859274	0.833497	-0.810355	26086	RP13-270L15	AGS3	HGNC:17858	9	9q34.3	G-protein sig	protein-codir	GPSM1	G-protein sig	O	G-protein sig	20130804
2	PLEKHG3	PLEKHG3	1.03525	-0.501505	-0.149444	0.533748	26030	-	ARHGEF43|K	HGNC:20364	14	14q23.3	pleckstrin ho	protein-codir	PLEKHG3	pleckstrin ho	O	PH domain-c	20130804
1	NELF	NSMF	1.22623	2.53367	2.50053	1.30744	26012	RP11-48C7.1	HH9|NELF	HGNC:29843	9	9q34.3	NMDA recep	protein-codir	NSMF	NMDA recep	O	nasal embryo	20130804
1	MDS2	MDS2	1.56885	-0.878716	-1.27389	-2.44757	259283	RP11-223J15	-	HGNC:29633	1	1p36	myelodyspla:	miscRNA	MDS2	myelodyspla:	O	-	20130226
1	DPCD	DPCD	1.00137	3.78656	3.32053	2.78519	25911	RP11-529I10.	-	HGNC:24542	10	10q24.32	deleted in pr	protein-codir	DPCD	deleted in pr	O	deleted in a r	20130804
1	METTL21B	METTL21B	1.05243	0.639899	0.509749	-0.412533	25895	-	FAM119B	HGNC:24936	12	12q14.1	methyltransf	protein-codir	METTL21B	methyltransf	O	family with s	20130804
1	DFNB31	DFNB31	1.74934	-0.660202	-1.20749	-2.40954	25861	RP11-9M16.1	CIP98|PDZD	HGNC:16361	9	9q32	deafness, aut	protein-codir	DFNB31	deafness, aut	O	CASK-interac	20130804
1	SNX33	SNX33	1.10937	0.146625	-0.196825	-0.962744	257364	-	SH3PX3|SH3	HGNC:28468	15	15q24.2	sorting nexin	protein-codir	SNX33	sorting nexin	O	SH3 and PX d	20130806
4	ALX3	ALX3	1.09905	-0.660046	-1.7591	-1.42533	257	-	FND1	HGNC:449|M	1	1p13.3	ALX homeob	protein-codir	ALX3	ALX homeob	O	aristaless-lik	20130522
1	C11orf35	C11orf35	1.82206	0.0817964	-0.789225	-1.74026	256329	-	-	HGNC:28561	11	11p15.5	chromosome	protein-codir	C11orf35	chromosome	O	uncharacteri:	20130226
1	TCP11L2	TCP11L2	1.42341	-1.10384	-1.71799	-2.52725	255394	-	-	HGNC:28627	12	12q23.3	t-complex 11	protein-codir	TCP11L2	t-complex 11	O	T-complex pr	20130804
4	MBLAC1	MBLAC1	1.06627	0.45471	-0.273337	0.792936	255374	-	-	HGNC:22180	7	7q22.1	metallo-beta	protein-codir	MBLAC1	metallo-beta	O	metallo-beta	20130226
1	GAA	GAA	1.02976	0.451491	0.149231	-0.578268	2548	-	LYAG	HGNC:4065|	17	17q25.2-q25	glucosidase,	protein-codir	GAA	glucosidase,	O	acid maltase	20130804
1	LOC254100	SSSCA1-AS1	1.82878	-1.86926	-1.04347	-2.87225	254100	-	-	HGNC:27384	11	11q13.1	SSSCA1 antis	miscRNA	SSSCA1-AS1	SSSCA1 antis	O	-	20130604
2	FUT7	FUT7	1.55661	-1.38196	-0.373448	0.174652	2529	-	FucT-VII	HGNC:4018|	9	9q34.3	fucosyltransf	protein-codir	FUT7	fucosyltransf	O	alpha-(1,3)-fu	20130804
2	SDF2L1	SDF2L1	1.53824	0.0612283	0.290703	1.59947	23753	UNQ1941/PF	-	HGNC:10676	22	22q11.21	stromal cell-	protein-codir	SDF2L1	stromal cell-	O	OTTHUMT00	20130804
4	FOS	FOS	1.41207	1.19016	-0.221912	-0.0239028	2353	-	AP-1|C-FOS|	HGNC:3796|	14	14q24.3	FBJ murine o	protein-codir	FOS	FBJ murine o	O	FBJ murine o	20130804
1	ABCB9	ABCB9	1.24281	1.95273	1.33198	0.709921	23457	-	EST122234|T	HGNC:50|MI	12	12q24	ATP-binding	protein-codir	ABCB9	ATP-binding	O	ABC transpor	20130804
1	SIRT4	SIRT4	1.42669	0.662419	0.34491	-0.764267	23409	-	SIR2L4	HGNC:14932	12	12q	sirtuin 4	protein-codir	SIRT4	sirtuin 4	O	NAD-depend	20130804
2	BOP1	BOP1	1.223	-0.124662	0.180708	1.09834	23246	-	-	HGNC:15519	8	8q24.3	block of proli	protein-codir	BOP1	block of proli	O	block of proli	20130804
2	RRP12	RRP12	1.16742	-0.478005	-0.0142313	0.689415	23223	RP11-452K12	KIAA0690	HGNC:29100	10	10q24.1	ribosomal RN	protein-codir	RRP12	ribosomal RN	O	RRP12-like pr	20130804
2	RRS1	RRS1	1.48536	-0.593718	0.0722326	0.891638	23212	-	-	HGNC:17083	8	8q13.1	RRS1 riboson	protein-codir	RRS1	RRS1 riboson	O	homolog of y	20130804
2	MLC1	MLC1	1.71619	-1.40605	-0.12153	0.310135	23209	RP3-355C18.	LVM|MLC|V	HGNC:17082	22	22q13.33	megalenceph	protein-codir	MLC1	megalenceph	O	membrane p	20130804
1	N4BP3	N4BP3	2.48224	3.4674	3.18402	0.985156	23138	-	LZTS4	HGNC:29852	5	5q35.3	NEDD4 bindi	protein-codir	N4BP3	NEDD4 bindi	O	NEDD4-bindi	20130804
1	KDM6B	KDM6B	1.16208	0.284853	-0.0753352	-0.877225	23135	-	JMJD3	HGNC:29012	17	17p13.1	lysine (K)-spe	protein-codir	KDM6B	lysine (K)-spe	O	jmjC domain-	20130804
2	RAP1GAP2	RAP1GAP2	1.0929	0.0385742	0.278024	1.13147	23108	-	GARNL4|RAF	HGNC:29176	17	17p13.3	RAP1 GTPase	protein-codir	RAP1GAP2	RAP1 GTPase	O	GTPase activ	20130804
1	ZNF292	ZNF292	1.06715	1.32715	0.98091	0.26	23036	-	Nbla00365|Z	HGNC:18410	6	6q14.3	zinc finger pr	protein-codir	ZNF292	zinc finger pr	O	16 zinc-finge	20130804
1	KDM4B	KDM4B	1.38262	1.66642	1.34215	0.283799	23030	-	JMJD2B|TDR	HGNC:29136	19	19p13.3	lysine (K)-spe	protein-codir	KDM4B	lysine (K)-spe	O	jmjC domain-	20130804
1	FOXJ1	FOXJ1	1.36388	2.96922	2.43765	1.60534	2302	-	FKHL13|HFH	HGNC:3816|	17	17q25.1	forkhead box	protein-codir	FOXJ1	forkhead box	O	fork head ho	20130804
1	GOLGA8A	GOLGA8A	1.0042	1.56289	1.4458	0.558694	23015	-	GM88	HGNC:31972	15	15q11.2	golgin A8 fan	protein-codir	GOLGA8A	golgin A8 fan	O	88 kDa Golgi	20130804
1	ALDOC	ALDOC	1.6284	4.57296	4.19054	2.94456	230	-	ALDC	HGNC:418|M	17	17cen-q12	aldolase C, fr	protein-codir	ALDOC	aldolase C, fr	O	aldolase 3|b	20130804
1	IGSF9B	IGSF9B	1.36863	2.58164	2.48426	1.21301	22997	UNQ1946	-	HGNC:32326	11	11q25	immunoglob	protein-codir	IGSF9B	immunoglob	O	immunoglob	20130613
2	CCT5	CCT5	1.12584	0.190129	0.597626	1.31597	22948	-	CCT-epsilon|	HGNC:1618|	5	5p15.2	chaperonin c	protein-codir	CCT5	chaperonin c	O	T-complex pr	20130804
1	ZBTB1	ZBTB1	1.08666	0.588743	0.39449	-0.497918	22890	-	ZNF909	HGNC:20259	14	14q23.3	zinc finger ar	protein-codir	ZBTB1	zinc finger ar	O	zinc finger ar	20130804
1	FGF11	FGF11	2.36163	6.63077	6.49047	4.26914	2256	-	FHF3	HGNC:3667|	17	17p13.1	fibroblast gro	protein-codir	FGF11	fibroblast gro	O	FGF-11|FHF-	20130803
1	C7orf41	C7orf41	1.32533	-0.929779	-1.43527	-2.25511	222166	-	Ells1	HGNC:25457	7	7p14.3	chromosome	protein-codir	C7orf41	chromosome	O	UPF0452 pro	20130804
1	ZNF498	ZSCAN25	1.0909	0.844334	0.53801	-0.246563	221785	-	ZNF498	HGNC:21961	7	7q22.1	zinc finger ar	protein-codir	ZSCAN25	zinc finger ar	O	zinc finger ar	20130709
1	FCGRT	FCGRT	1.12762	0.674459	0.461356	-0.453163	2217	-	FCRN|alpha-	HGNC:3621|	19	19q13.3	Fc fragment	protein-codir	FCGRT	Fc fragment	O	FcRn alpha cl	20130804
1	CPNE2	CPNE2	1.05947	2.36948	2.61132	1.55185	221184	-	COPN2|CPN	HGNC:2315|	16	16q13	copine II	protein-codir	CPNE2	copine II	O	copine-2	20130804
2	OAF	OAF	1.00699	-0.798964	0.111453	0.208027	220323	-	NS5ATP13TP	HGNC:28752	11	11q23.3	OAF homolog	protein-codir	OAF	OAF homolog	O	HCV NS5A-tr	20130226
2	ESRRA	ESRRA	1.02314	-0.0949044	0.438455	0.928235	2101	-	ERR1|ERRa|	HGNC:3471|	11	11q13	estrogen-rela	protein-codir	ESRRA	estrogen-rela	O	ERR-alpha|e	20130807
1	EPHX2	EPHX2	1.31964	-0.384046	-0.798541	-1.70369	2053	-	CEH|SEH	HGNC:3402|	8	8p21	epoxide hydr	protein-codir	EPHX2	epoxide hydr	O	bifunctional	20130804
1	EPHX1	EPHX1	1.65594	-0.290881	-0.961204	-1.94682	2052	-	EPHX|EPOX|	HGNC:3401|	1	1q42.1	epoxide hydr	protein-codir	EPHX1	epoxide hydr	O	epoxide hydr	20130804
1	ENO2	ENO2	1.38495	2.97038	2.72896	1.58543	2026	-	NSE	HGNC:3353|	12	12p13	enolase 2 (ga	protein-codir	ENO2	enolase 2 (ga	O	2-phospho-D	20130804
4	FAM209A	FAM209A	1.5262	-1.21701	-2.74321	-1.82786	200232	RP5-1153D9.	C20orf106|d	HGNC:16100	20	20q13.31	family with s	protein-codir	FAM209A	family with s	O	protein FAM	20130226
2	ALG14	ALG14	1.12414	0.100503	0.395299	1.22464	199857	-	-	HGNC:28287	1	1p21.3	ALG14, UDP-	protein-codir	ALG14	ALG14, UDP-	O	UDP-N-acety	20130804
1	THAP8	THAP8	1.32485	1.34375	0.87887	0.0188995	199745	-	-	HGNC:23191	19	19q13.12	THAP domair	protein-codir	THAP8	THAP domair	O	THAP domair	20130804
1	EGR1	EGR1	1.38748	1.16878	0.603916	-0.218699	1958	-	AT225|G0S3	HGNC:3238|	5	5q31.1	early growth	protein-codir	EGR1	early growth	O	EGR-1|early	20130804
1	EFNA4	EFNA4	1.07394	1.46567	1.14767	0.391728	1945	-	EFL4|EPLG4|	HGNC:3224|	1	1q21-q22	ephrin-A4	protein-codir	EFNA4	ephrin-A4	O	LERK-4|eph-	20130804
1	EFNA3	EFNA3	1.15252	2.19757	2.2475	1.09498	1944	-	EFL2|EPLG3|	HGNC:3223|	1	1q21-q22	ephrin-A3	protein-codir	EFNA3	ephrin-A3	O	EFL-2|EHK1 l	20130804
1	DUSP3	DUSP3	1.38268	2.47212	2.24424	1.08944	1845	-	VHR	HGNC:3069|	17	17q21	dual specifici	protein-codir	DUSP3	dual specifici	O	dual specifici	20130804
1	TSC22D3	TSC22D3	2.21598	0.145459	-0.604854	-2.07052	1831	RP13-364K2	DIP|DSIPI|GI	HGNC:3051|	X	Xq22.3	TSC22 domai	protein-codir	TSC22D3	TSC22 domai	O	DSIP-immun	20130804
2	NQO1	NQO1	1.32336	0.150226	0.405139	1.47359	1728	-	DHQU|DIA4|	HGNC:2874|	16	16q22.1	NAD(P)H deh	protein-codir	NQO1	NAD(P)H deh	O	DT-diaphora	20130804
1	FUT11	FUT11	1.25553	2.5925	2.37486	1.33697	170384	-	-	HGNC:19233	10	10q22.2	fucosyltransf	protein-codir	FUT11	fucosyltransf	O	alpha-(1,3)-fu	20130804
1	CFD	CFD	1.36223	2.19492	2.20331	0.841085	1675	-	ADIPSIN|ADI	HGNC:2771|	19	19p13.3	complement	protein-codir	CFD	complement	O	C3 convertas	20130804

1	PAQR7	PAQR7	1.55563	2.23251	1.64652	0.676881	164091	RP1-125I3.1	MPRA|PGLP	HGNC:23146	1	1p36.11	progestin an	protein-codir	PAQR7	progestin an	O	2310021M12	20130804
4	ZNF579	ZNF579	1.07691	1.2852	0.208291	0.475865	163033	-	-	HGNC:26646	19	19q13.42	zinc finger pr	protein-codir	ZNF579	zinc finger pr	O	-	20130804
1	OTUD7A	OTUD7A	2.01667	5.49777	5.34655	3.4811	161725	-	C15orf16|C1	HGNC:20718	15	15q13.3	OTU domain	protein-codir	OTUD7A	OTU domain	O	OTU domain-	20130804
2	CLECL1	CLECL1	1.71265	-1.98989	-2.55791	-0.845256	160365	-	DCAL-1|DCA	HGNC:24462	12	12p13.31	C-type lectin-	protein-codir	CLECL1	C-type lectin-	O	C-type lectin-	20130226
1	CLEC12A	CLEC12A	1.56297	2.65361	2.51292	1.09064	160364	-	CLL-1|CLL1|I	HGNC:31713	12	12p13.2	C-type lectin	protein-codir	CLEC12A	C-type lectin	O	C-type lectin	20130707
1	KIAA1958	KIAA1958	1.2346	2.17028	1.90778	0.935678	158405	RP11-276E15	-	HGNC:23427	9	9q32	KIAA1958	protein-codir	KIAA1958	KIAA1958	O	uncharacteri:	20130804
1	ADRBK2	ADRBK2	1.12106	2.36457	1.95209	1.24351	157	-	BARK2|GRK3	HGNC:290|M	22	22q12.1	adrenergic, b	protein-codir	ADRBK2	adrenergic, b	O	G-protein-co	20130804
1	LOC154761	LOC154761	1.01342	0.803176	0.540517	-0.210248	154761	-	-	Ensembl:ENS	7	7q35	family with s	pseudo	-	-	-	-	20130226
1	CYP1A1	CYP1A1	2.5509	5.84743	4.80962	3.29653	1543	-	AHH|AHRR|(	HGNC:2595|	15	15q24.1	cytochrome I	protein-codir	CYP1A1	cytochrome I	O	CYPIA1|aryl I	20130803
2	SAMD3	SAMD3	1.06575	-1.21348	-0.63967	-0.147728	154075	RP11-73O6.2	-	HGNC:21574	6	6q23.1	sterile alpha	protein-codir	SAMD3	sterile alpha	O	SAM domain	20130804
1	GPR155	GPR155	1.15296	-0.643671	-0.786261	-1.79663	151556	-	DEP.7|DEPD(	HGNC:22951	2	2q31.1	G protein-co	protein-codir	GPR155	G protein-co	O	G-protein co	20130804
2	CTSL1	CTSL	2.44809	-0.778835	-0.0950686	1.66926	1514	RP11-65B23.	CATL|CTSL1|	HGNC:2537|	9	9q21.33	cathepsin L	protein-codir	CTSL	cathepsin L	O	cathepsin L1	20130804
2	CTSH	CTSH	1.22874	0.653056	1.1422	1.8818	1512	-	ACC-4|ACC-5	HGNC:2535|	15	15q25.1	cathepsin H	protein-codir	CTSH	cathepsin H	O	N-benzoylarg	20130804
1	SIK1	SIK1	1.65728	3.98988	3.80549	2.3326	150094	-	MSK|SIK|SNI	HGNC:11142	21	21q22.3	salt-inducible	protein-codir	SIK1	salt-inducible	O	SIK-1|SNF1-li	20130804
1	C1orf51	C1orf51	1.72964	4.58336	4.17586	2.85372	148523	RP4-790G17.	-	HGNC:25200	1	1q21.2	chromosome	protein-codir	C1orf51	chromosome	O	uncharacteri:	20130804
1	KCTD11	KCTD11	1.09193	2.06285	1.80138	0.970924	147040	-	C17orf36|KC	HGNC:21302	17	17p13.1	potassium ch	protein-codir	KCTD11	potassium ch	O	BTB/POZ dor	20130804
1	PIK3R6	PIK3R6	1.9711	4.0532	3.50884	2.0821	146850	-	C17orf38|Hs	HGNC:27101	17	17p13.1	phosphoinos	protein-codir	PIK3R6	phosphoinos	O	PI3Kgamma a	20130803
1	FBXL16	FBXL16	1.09801	0.155157	0.318265	-0.77975	146330	-	C16orf22|Fb	HGNC:14150	16	16p13.3	F-box and le	protein-codir	FBXL16	F-box and le	O	F-box/LRR-re	20130804
1	LAYN	LAYN	1.45406	2.17699	1.67534	0.722928	143903	UNQ208/PR(	-	HGNC:29471	11	11q23.1	layilin	protein-codir	LAYN	layilin	O	-	20130804
2	CSF2RB	CSF2RB	1.24529	0.403106	0.928249	1.6484	1439	CITF22-45C1.	CD131|CDw1	HGNC:2436|	22	22q13.1	colony stimu	protein-codir	CSF2RB	colony stimu	O	GM-CSF/IL-3,	20130804
2	CSF2	CSF2	2.01632	0.750293	1.2694	2.76661	1437	-	GMCSF	HGNC:2434|	5	5q31.1	colony stimu	protein-codir	CSF2	colony stimu	O	CSF|granulo(	20130804
2	CSF1	CSF1	1.21307	0.332175	0.866836	1.54525	1435	RP11-195M1	CSF-1|MCSF	HGNC:2432|	1	1p13.3	colony stimu	protein-codir	CSF1	colony stimu	O	lanimostim|r	20130806
4	LINC00266-1	LINC00266-1	1.27441	-0.714253	-1.98866	-1.85199	140849	-	C20orf69|NC	HGNC:16202	20	20q13.33	long interger	miscRNA	LINC00266-1	long interger	O	-	20130430
1	KRT72	KRT72	1.02368	-1.86875	-1.61237	-2.63605	140807	-	K6IRS2|K6irs	HGNC:28932	12	12q13.13	keratin 72	protein-codir	KRT72	keratin 72	O	CK-72|K72|c	20130804
2	CRIP2	CRIP2	1.02933	2.67448	2.43768	3.46701	1397	-	CRIP|CRP2|E	HGNC:2361|	14	14q32.3	cysteine-rich	protein-codir	CRIP2	cysteine-rich	O	Cysteine-rich	20130804
2	IRAK1BP1	IRAK1BP1	1.15105	-1.19367	-1.09396	-0.0426244	134728	-	AIP70|SIMPL	HGNC:17368	6	6q14-q15	interleukin-1	protein-codir	IRAK1BP1	interleukin-1	O	ActA binding	20130804
2	SETD9	SETD9	1.18785	0.156381	0.813501	1.34423	133383	-	C5orf35	HGNC:28508	5	5q11.2	SET domain c	protein-codir	SETD9	SET domain c	O	SET domain-c	20130804
1	ADM	ADM	3.79975	6.99212	6.35421	3.19237	133	-	AM	HGNC:259|M	11	11p15.4	adrenomedu	protein-codir	ADM	adrenomedu	O	preproadren	20130728
4	FAM43A	FAM43A	1.11756	0.118286	-0.720465	0.397091	131583	PP7298	-	HGNC:26888	3	3q29	family with s	protein-codir	FAM43A	family with s	O	protein FAM	20130226
3	SGPP2	SGPP2	1.05314	-1.22024	-0.167098	-0.333077	130367	-	SPP2	HGNC:19953	2	2q36.1	sphingosine-	protein-codir	SGPP2	sphingosine-	O	SPPase2|hSP	20130416
1	GIPC3	GIPC3	1.17834	1.01241	0.564623	-0.165928	126326	-	C19orf64|DF	HGNC:18183	19	19p13.3	GIPC PDZ dor	protein-codir	GIPC3	GIPC PDZ dor	O	PDZ domain-	20130804
1	PCP2	PCP2	2.92962	1.30108	0.877777	-1.62854	126006	-	GPSM4	HGNC:30209	19	19p13.2	Purkinje cell	protein-codir	PCP2	Purkinje cell	O	Purkinje cell	20130804
2	CCR6	CCR6	1.17909	-1.35097	-0.987373	-0.17188	1235	-	BN-1|C-C CK	HGNC:1607|	6	6q27	chemokine ((	protein-codir	CCR6	chemokine ((	O	C-C chemoki	20130804
2	CCR1	CCR1	1.00633	1.64801	1.96097	2.65434	1230	-	CD191|CKR-1	HGNC:1602|	3	3p21	chemokine ((	protein-codir	CCR1	chemokine ((	O	C-C CKR-1|C-	20130804
1	ACOT4	ACOT4	1.02354	3.03529	2.69657	2.01175	122970	-	PTE-Ib|PTE1I	HGNC:19748	14	14q24.3	acyl-CoA thic	protein-codir	ACOT4	acyl-CoA thic	O	PTE-2b|acyl-	20130804
2	CKS2	CKS2	1.08946	0.575085	1.05278	1.66455	1164	RP11-8C21.2	CKSHS2	HGNC:2000|	9	9q22	CDC28 prote	protein-codir	CKS2	CDC28 prote	O	CDC28 prote	20130804
2	C5orf55	C5orf55	1.07063	-0.560314	-0.622235	0.448399	116349	-	-	HGNC:25175	5	5p15.33	chromosome	protein-codir	C5orf55	chromosome	O	uncharacteri:	20130804
1	TNFRSF13C	TNFRSF13C	1.2746	-0.349237	-1.07921	-1.62384	115650	CTA-250D10.	BAFF-R|BAFF	HGNC:17755	22	22q13.1-q13	tumor necro:	protein-codir	TNFRSF13C	tumor necro:	O	B cell-activat	20130804
1	OSBPL5	OSBPL5	1.21108	0.897763	0.444484	-0.313316	114879	-	OBPH1|ORP5	HGNC:16392	11	11p15.4	oxysterol bin	protein-codir	OSBPL5	oxysterol bin	O	ORP-5|OSBP	20130804
2	TMEM200A	TMEM200A	1.12254	0.68781	0.970691	1.81035	114801	HBE61	KIAA1913|TI	HGNC:21075	6	6q23.1	transmembra	protein-codir	TMEM200A	transmembra	O	two transme	20130226
1	LMTK3	LMTK3	1.26938	0.219897	0.0434708	-1.04948	114783	-	LMR3|TYKLN	HGNC:19295	19	19q13.33	lemur tyrosir	protein-codir	LMTK3	lemur tyrosir	O	serine/threo	20130804
1	SLC2A13	SLC2A13	1.52498	1.2559	0.805931	-0.269076	114134	-	HMIT	HGNC:15956	12	12q12	solute carrier	protein-codir	SLC2A13	solute carrier	O	H(+)-myo-inc	20130226
1	IZUMO4	IZUMO4	1.84611	2.01919	1.75076	0.173083	113177	UNQ831/PR(	C19orf36|IM	HGNC:26950	19	19p13.3	IZUMO famil	protein-codir	IZUMO4	IZUMO famil	O	izumo sperm	20130622
2	FDX1L	FDX1L	1.73821	-1.44042	-0.692542	0.297794	112812	-	FDX2	HGNC:30546	19	19p13.2	ferredoxin 1-	protein-codir	FDX1L	ferredoxin 1-	O	adrenodoxin-	20130804
2	IL17F	IL17F	2.03878	2.5258	4.14725	4.56458	112744	-	CANDF6|IL-1	HGNC:16404	6	6p12	interleukin 1	protein-codir	IL17F	interleukin 1	O	IL-24|cytokir	20130630
1	EGLN3	EGLN3	2.12738	2.19357	1.93691	0.0661949	112399	-	HIFP4H3|HIF	HGNC:14661	14	14q13.1	egl nine hom	protein-codir	EGLN3	egl nine hom	O	HIF prolyl hy	20130804
1	RNF24	RNF24	1.04137	0.68246	0.594301	-0.358909	11237	-	G1L	HGNC:13779	20	20p13	ring finger pr	protein-codir	RNF24	ring finger pr	O	RING finger p	20130804
2	TREX2	TREX2	1.09381	-0.377953	0.196891	0.715856	11219	-	-	HGNC:12270	X	Xq28	three prime r	protein-codir	TREX2	three prime r	O	3'-5' exonucl	20130804
1	HHLA3	HHLA3	1.03863	-0.020145	0.165594	-0.873035	11147	RP11-180O5.	-	HGNC:4906|	1	1p31.1	HERV-H LTR-a	protein-codir	HHLA3	HERV-H LTR-a	O	HERV-H LTR-a	20130804
1	UPK1A	UPK1A	2.38391	3.9747	3.1531	1.59079	11045	-	TSPAN21|UP	HGNC:12577	19	19q13.13	uroplakin 1A	protein-codir	UPK1A	uroplakin 1A	O	tetraspanin-2	20130806
2	SLC27A4	SLC27A4	1.55257	-1.12368	-0.481578	0.428889	10999	RP11-339B21	ACSVL4|FATI	HGNC:10998	9	9q34.11	solute carrier	protein-codir	SLC27A4	solute carrier	O	long-chain fa	20130804
2	EBNA1BP2	EBNA1BP2	1.1633	0.0229924	0.456947	1.18629	10969	RP5-1034F7.	EBP2|NOBP|	HGNC:15531	1	1p35-p33	EBNA1 bindir	protein-codir	EBNA1BP2	EBNA1 bindir	O	EBNA1-bindi	20130804
1	PNRC1	PNRC1	1.18243	-0.129264	-0.475556	-1.31169	10957	RP11-63L7.5	B4-2|PNAS-1	HGNC:17278	6	6q15	proline-rich r	protein-codir	PNRC1	proline-rich r	O	proline rich 2	20130804
4	SLC26A1	SLC26A1	1.06379	-1.61755	-2.68134	-1.63432	10861	-	EDM4|SAT-1	HGNC:10993	4	4p16.3	solute carrier	protein-codir	SLC26A1	solute carrier	O	solute carrier	20130804
1	PPP1R13L	PPP1R13L	1.20336	2.04634	1.94846	0.842979	10848	-	IASPP|NKIP1	HGNC:18838	19	19q13.32	protein phos	protein-codir	PPP1R13L	protein phos	O	NFkB interac	20130806
1	NOXA1	NOXA1	2.04288	0.903993	0.202333	-1.13889	10811	-	NY-CO-31|SD	HGNC:10668	9	9q34.3	NADPH oxida	protein-codir	NOXA1	NADPH oxida	O	NCF2-like pro	20130804
2	HSPH1	HSPH1	1.36209	-0.543553	0.00031475	0.818536	10808	RP11-173P16	HSP105|HSP	HGNC:16969	13	13q12.3	heat shock 1(	protein-codir	HSPH1	heat shock 1(	O	antigen NY-C	20130804
2	RPP40	RPP40	1.46006	-1.01082	-0.60993	0.449236	10799	RP11-428J1.3	RNASEP1|bA	HGNC:20992	6	6p25.1	ribonuclease	protein-codir	RPP40	ribonuclease	O	RNase P sub	20130804
2	WDR4	WDR4	1.17468	-0.0849116	0.056245	1.08977	10785	-	TRM82|TRM	HGNC:12756	21	21q22.3	WD repeat d	protein-codir	WDR4	WD repeat d	O	TRM82 tRNA	20130804
1	TXNIP	TXNIP	1.15854	0.401359	-0.0429285	-0.75718	10628	RP11-315I20.	EST01027|HI	HGNC:16952	1	1q21.1	thioredoxin i	protein-codir	TXNIP	thioredoxin i	O	thioredoxin b	20130804
1	DPYSL4	DPYSL4	2.3269	6.42718	6.16453	4.10028	10570	RP11-140A1(	CRMP3|DRP-	HGNC:3016|	10	10q26	dihydropyrim	protein-codir	DPYSL4	dihydropyrim	O	CRMP-3|ULII	20130804
2	SSSCA1	SSSCA1	1.06036	-0.508188	-0.0079995	0.552175	10534	-	p27	HGNC:11328	11	11q13.1	Sjogren synd	protein-codir	SSSCA1	Sjogren synd	O	Sjoegren syn	20130804
2	HYOU1	HYOU1	1.04249	-0.786708	-0.472247	0.25578	10525	-	GRP-170|Gr	HGNC:16931	11	11q23.1-q23	hypoxia up-r	protein-codir	HYOU1	hypoxia up-r	O	150 kDa oxyg	20130804
1	FBLN5	FBLN5	1.09327	0.559589	0.0691702	-0.533685	10516	UNQ184/PR(	ADCL2|ARCL	HGNC:3602|	14	14q32.1	fibulin 5	protein-codir	FBLN5	fibulin 5	O	development	20130804
2	MYBBP1A	MYBBP1A	1.01927	-0.654292	-0.163265	0.364973	10514	-	P160|PAP2	HGNC:7546|	17	17p13.3	MYB binding	protein-codir	MYBBP1A	MYB binding	O	myb-binding	20130804
2	SNAPC5	SNAPC5	1.00199	-0.705576	-0.491891	0.296412	10302	-	SNAP19	HGNC:15484	15	15q22.31	small nuclear	protein-codir	SNAPC5	small nuclear	O	SNAPc 19 kD	20130804
2	ZMPSTE24	ZMPSTE24	1.02864	-0.948409	-0.62278	0.0802272	10269	-	FACE-1|FACE	HGNC:12877	1	1p34	zinc metallop	protein-codir	ZMPSTE24	zinc metallop	O	CAAX prenyl	20130804
2	TRIB1	TRIB1	1.11415	-1.92018	-2.28967	-1.17552	10221	-	C8FW|GIG2|	HGNC:16891	8	8q24.13	tribbles hom	protein-codir	TRIB1	tribbles hom	O	G-protein-co	20130804
2	RCL1	RCL1	1.17613	-1.01768	-0.729206	0.158448	10171	RP11-125K1(	RNAC|RPCL1	HGNC:17687	9	9p24.1-p23	RNA termina	protein-codir	RCL1	RNA termina	O	2310040A02	20130804
4	EBI3	EBI3	1.52686	1.29906	-0.227804	0.23878	10148	-	IL-27B|IL27B	HGNC:3129|	19	19p13.3	Epstein-Barr	protein-codir	EBI3	Epstein-Barr	O	EBV-induced	20130804
1	FAM13A	FAM13A	1.59095	1.81087	1.56625	0.219923	10144	-	ARHGAP48|F	HGNC:19367	4	4q22.1	family with s	protein-codir	FAM13A	family with s	O	FAM13A1_v2	20130804
2	KIF20A	KIF20A	1.03611	1.9417	2.3894	2.97781	10112	-	MKLP2|RAB(	HGNC:9787|	5	5q31	kinesin famil	protein-codir	KIF20A	kinesin famil	O	GG10_2|RAE	20130804
3	SEC24B-AS1	SEC24B-AS1	1.17388	-0.780438	0.393441	-0.274054	100533182	-	1/2-SBSRNA4	HGNC:44003	4	4q25	SEC24B antis	miscRNA	SEC24B-AS1	SEC24B antis	O	-	20130717
2	RAB11B-AS1	RAB11B-AS1	1.00164	-0.277593	0.319975	0.724048	100507567	-	-	HGNC:44178	19	-	RAB11B antis	miscRNA	RAB11B-AS1	RAB11B antis	O	-	20130217
3	LOC1005071	LINC01012	1.33248	-1.00968	0.322798	-1.00078	100507173	-	-	HGNC:48986	6	-	long interger	miscRNA	LINC01012	long interger	O	-	20130727
1	MIR210HG	MIR210HG	1.63069	7.67108	7.49679	6.04039	100506211	-	-	HGNC:39524	11	11p15.5	MIR210 host	miscRNA	MIR210HG	MIR210 host	O	-	20130217
4	LOC1002893	LOC1002893	2.14171	-0.913993	-3.04892	-0.907209	100289361	-	-	Ensembl:ENS	3	3q23	uncharacteri:	miscRNA	-	-	-	-	20130216
1	LOC1002891	LOC1002891	1.64998	1.46996	0.697497	-0.180019	100289187	-	-	-	7	7q22.1	transmembra	protein-codir	-	-	-	-	20130216
1	LOC1002871	LOC1002871	1.17975	2.94804	2.33659	1.76829	100287177	-	-	Ensembl:ENS	19	19q13.32	uncharacteri:	protein-codir	-	-	-	uncharacteri:	20130430
2	TROAP	TROAP	1.03302	1.34013	1.9936	2.37315	10024	-	TASTIN	HGNC:12327	12	12q13.12	trophinin ass	protein-codir	TROAP	trophinin ass	O	tastin|trophi	20130804
2	NDUFB2-AS1	NDUFB2-AS1	2.50909	-2.22612	-0.0471179	0.282974	100134713	-	-	HGNC:40396	7	7q34	NDUFB2 anti	miscRNA	NDUFB2-AS1	NDUFB2 anti	O	-	20130430
1	LOC1001314	LINC00893	1.44778	-0.497695	-1.00631	-1.94547	100131434	-	-	HGNC:44265	X	Xq28	long interger	miscRNA	LINC00893	long interger	O	-	20130520
1	LOC1001304	LOC1001304	1.37385	-0.500428	-1.04231	-1.87428	100130476	-	-	-	6	6q23.3	uncharacteri:	miscRNA	-	-	-	-	20130217
1	LOC1001280	FAM229A	1.39099	0.120748	0.737621	-0.653369	100128071	-	-	HGNC:44652	1	1p35.1	family with s	protein-codir	FAM229A	family with s	O	protein FAM	20130316
2	LOC1000096	ZBTB11-AS1	1.44913	-1.52233	-0.395343	-0.0732029	100009676	-	-	HGNC:48573	3	3q12.3	ZBTB11 antis	miscRNA	ZBTB11-AS1	ZBTB11 antis	O	-	20130520
