# Supplementary material for: A GMCSF and IL7 fusion cytokine leads to functional thymic-dependent T-cell regeneration in age-associated immune deficiency
Source: Clin Transl Immunology. 2015 May 8;4(5):e37–. doi: 10.1038/cti.2015.8 (PMC4478872; doi:10.1038/cti.2015.8)
Supplement: Supplementary Table 1 Legend [file cti20158x4.doc]

**Supplementary Table 1. Genes with maximal difference in expression between GIFT7, IL7 or IL7+GMCSF treatment groups**

Genes were ranked by the maximum difference (MaxDiff) between the three treatment responses (IL7, GMCSF+IL7, and GIFT7). We then selected the 560 genes where MaxDiff was at least 1 in a log2 scale, which represents at least a two fold differential response. Treatment responses were estimated as the ratio between the expression level for a treatment condition to the expression level for the media sample.
